# Ablation of Cbl-b and c-Cbl in dendritic cells causes spontaneous liver cirrhosis via altering multiple properties of CD103^+^ cDC1s

**DOI:** 10.1038/s41420-022-00953-2

**Published:** 2022-03-30

**Authors:** Fei Xu, Chen Liu, Yongli Dong, Wenyan Wu, Jie Xu, Yunqiu Yan, Yu Shao, Chuangli Hao, Yi Yang, Jinping Zhang

**Affiliations:** 1 Institutes of Biology and Medical Sciences, Soochow University, Suzhou, People’s Republic of China; 2 Department of Respiratory Medicine, Children’s Hospital of Soochow University, Suzhou, People’s Republic of China

**Keywords:** Chronic inflammation, Immune cell death

## Abstract

The Casitas B-lineage lymphoma (Cbl) family proteins are E3 ubiquitin ligases implicated in the regulation of various immune cells. However, their function in dendritic cells (DCs) remains unclear. To investigate the role of Cbl family members in DCs, we created dendritic cell double-deficient Casitas B lymphoma-b (Cbl-b) and Casitas B lineage lymphoma (c-Cbl) mice by crossing Cbl-b^−/−^ mice with c-Cbl^flox/flox^ CD11c-Cre^+^ mice. We found that specific deletion of Cbl-b and c-Cbl in CD11c^+^ cells, predominantly in DCs, led to liver fibrosis, cirrhosis, and accumulation of systemic conventional Type I DCs (cDC1s) due to enhanced cell proliferation and decreased cell apoptosis. In addition to a change in DC number, double knockout (dKO) cDC1s exhibited a partially activated status as indicated by high basal expression levels of certain cytokines and possessed an enhanced capacity to prime T cells. After adoptive transfer, dKO cDC1s could drive liver fibrosis too. In further experiments, we demonstrated that Cbl-b and c-Cbl could target signal transducer and activator of transcription 5 (STAT5), a transcriptional repressor for the pro-apoptotic protein Bim, to promote ubiquitination-mediated degradation and cell apoptosis in cDC1s. Further extensive experiments revealed that Cbl-b mediated K27-linked ubiquitination of lysine 164 of STAT5a while c-Cbl induced K29-linked ubiquitination of lysine 696 of STAT5a and K27-linked ubiquitination of lysine 140 and 694 of STAT5b. Thus, our findings indicate a functional redundancy of Cbl-b and c-Cbl in cDC homeostasis and maturation.

## Introduction

Dendritic cells (DCs) are a highly heterogeneous group of cells that are classified into at least two subsets, namely plasmacytoid (pDCs) and conventional (classical) DCs (cDCs). In mice, CD11c represents a relatively specific DC marker, with cDCs and pDCs being defined as CD11c^high^ MHC-II^+^ and CD11c^int^ PDCA-1^+^ B220^+^ cells, respectively [1]. Based on their functional ontogeny, cDCs can be further subdivided into two subsets, cDC1s and cDC2s. This classification is further supported by the subsequent identification of transcription factor profiles required for the development of each cDC subset [2]. In lymphoid tissues, cDC1s and cDC2s are defined as CD8^+^ CD205^+^ and CD8^−^CD11b^high^, respectively, which are analogous to CD103^+^ CD11b^−^ and CD103^−^ CD11b^+^ DCs in non-lymphoid tissues, respectively [3].

DCs function as key players in immune system. Firstly, they can either directly mediate innate immunity by secreting anti-viral interferons and defensins or enhance innate immunity by influencing a broad range of other cell types, such as NK cells [4], NKT cells [5], and macrophages [6], via cytokines or cell-cell contact. Secondly, as the most potent antigen-presenting cells for T cells, they link innate and adaptive immune responses and play an important role in the maintenance of the immunological balance. In addition to their maturation and/or activation status, the total number of DCs has also been shown to be critical for the regulation of immune system [7–9]. Thus, to ensure the proper function of immune system, the homeostasis, maturation, and total number of DCs must be tightly regulated. Currently, there remain large gaps in our understanding of the mechanisms underlying the regulation of these processes in DCs.

The mammalian Casitas B-Lymphoma (Cbl) protein family, which consists of three members, c-Cbl, Cbl-b, and Cbl-c, act as critical negative regulators in various signaling pathways [10–14]. So far, a few studies have explored the role of Cbl family in DCs. It has been reported that c-Cbl-deficient (c-Cbl^−/−^) bone-marrow-derived DCs (BMDCs) produce higher levels of both basal and activation-induced proinflammatory cytokines, likely due to a failure of processing of p105 to p50, which functions as homodimers to suppress NF-κB response [14]. Likewise, Cbl-b-deficient (Cbl-b^−/−^) BMDCs produce higher levels of proinflammatory cytokines upon TLR engagement [15]. Whether the Cbl family regulates other aspects of DCs and how members of the Cbl family cooperate in DCs have remained unclear so far.

Here we found mice with deficiency in Cbl-b and c-Cbl in DCs developed severe liver fibrosis characterized by an accumulation of CD103^+^ cDC1s in the liver. Moreover, we found that cellular activation and proliferation of dKO DCs were increased while cell apoptosis was decreased, which we speculated directly drive liver fibrosis. And this speculation was confirmed by the occurrence of liver fibrosis after the adoptive transfer of dKO cDCs to wild type (WT) recipient mice. Mechanistically, we found that Cbl-b and c-Cbl influenced mitochondrial-dependent apoptosis of CD103^+^ cDC1s via negative regulation of the expression of STAT5, a transcriptional repressor for the pro-apoptotic molecule Bim, which negatively regulates DC apoptosis [16]. Taken together, our data suggest that Cbl-b and c-Cbl exhibit functional redundancy in cDC1 homeostasis and maturation.

## Results

### Cbl-b and c-Cbl double-deficient dendritic cell mice develop liver cirrhosis

**Fig. 1:**
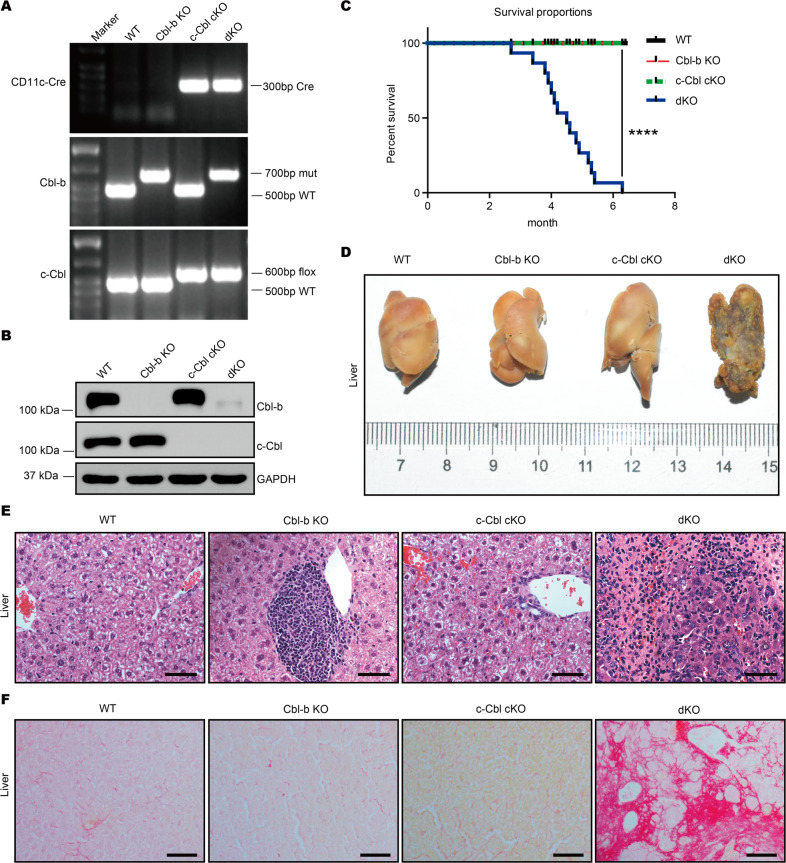
Cbl-b and c-Cbl deficiency in DCs results to liver cirrhosis and untimely death of mice. **A** Genomic DNA was extracted and PCR was performed to detect levels of Cbl-b and c-Cbl in mice. **B** Bone marrow cells were cultured with 10 ng/ml GM-CSF for 7 days followed by magnetic bead isolation of CD11c-positive cells. Protein levels of Cbl-b and c-Cbl in CD11c^+^ cells were quantified by Western blot. **C** Survival curves of four groups of mice (*n* = 17 per group). **D** Livers of mice of all four genotypes were dissected for morphologic examination (*n* = 3 per group). **E** Livers were removed and fixed with 10% formalin. Paraffin sections were stained with hematoxylin and eosin (*n* = 3 per group); scale bar, 50 μm. **F** Livers were dissected and fixed with 10% formalin. Paraffin sections were stained with Sirius red for 1 h (*n* = 3 per group); scale bar, 100 μm. ****p < 0.0001 by log-rank tests. *p* < *0.05* was considered statistically significant.

To investigate whether and how Cbl-b and c-Cbl influence DC homeostasis and function, we crossed Cbl-b^−/−^ mice with c-Cbl^flox/flox^ CD11c-Cre^+^ mice to obtain four groups of mice: Cbl-b^+/+^ c-Cbl^flox/flox^ CD11c-Cre^−^ (WT), Cbl-b^−/−^ c-Cbl^flox/flox^ CD11c-Cre^−^ (Cbl-b KO), Cbl-b^+/+^ c-Cbl^flox/flox^ CD11c-Cre^+^ (c-Cbl conditional knockout or cKO), and Cbl-b^−/−^ c-Cbl^flox/flox^ CD11c-Cre^+^ mice (dKO) (Fig. 1A). Deletion of c-Cbl mediated by CD11c-Cre has been shown to predominantly occur in DCs [17], and deletion of both Cbl-b and c-Cbl in CD11c^+^ cells at the protein level was confirmed by Western blotting (Fig. 1B). Data shown in Fig. 1A, B confirmed the successful generation of Cbl-b KO, c-Cbl cKO, and dKO mice.

To our surprise, as early as three months of age, some dKO mice started to exhibit significant signs of illness including notable weight loss, a substantial reduction of motor activity, disheveled coats, and skin jaundice, and all dKO mice died by seven months of age (Fig. 1C). In contrast, the other three groups of mice were generally healthy throughout, which implied that Cbl-b and c-Cbl may function in a complementary manner in DCs. To explore the reason for the early death of dKO mice, we dissected and examined several key organs including the kidney, liver, lung, pancreas, and salivary glands. Morphological examination revealed nodular atrophy in the liver of 4-month-old dKO mice (Fig. 1D). Histology showed that the normal hepatic architecture was replaced by regenerative nodules bounded by fibrous septa in the livers of dKO mice and furthermore revealed an infiltration of inflammatory cells (Fig. 1E). Moreover, Sirius red staining revealed an abundance of collagen deposition in the livers of dKO mice (Fig. 1F). We observed no major histological abnormalities in salivary glands, pancreases, lungs, or kidneys stained with hematoxylin and eosin (H&E) (Fig. S1). These data suggested dKO mice spontaneously developed liver fibrosis and cirrhosis.

### dKO cDCs accumulate in large numbers in the liver and spleen

**Fig. 2:**
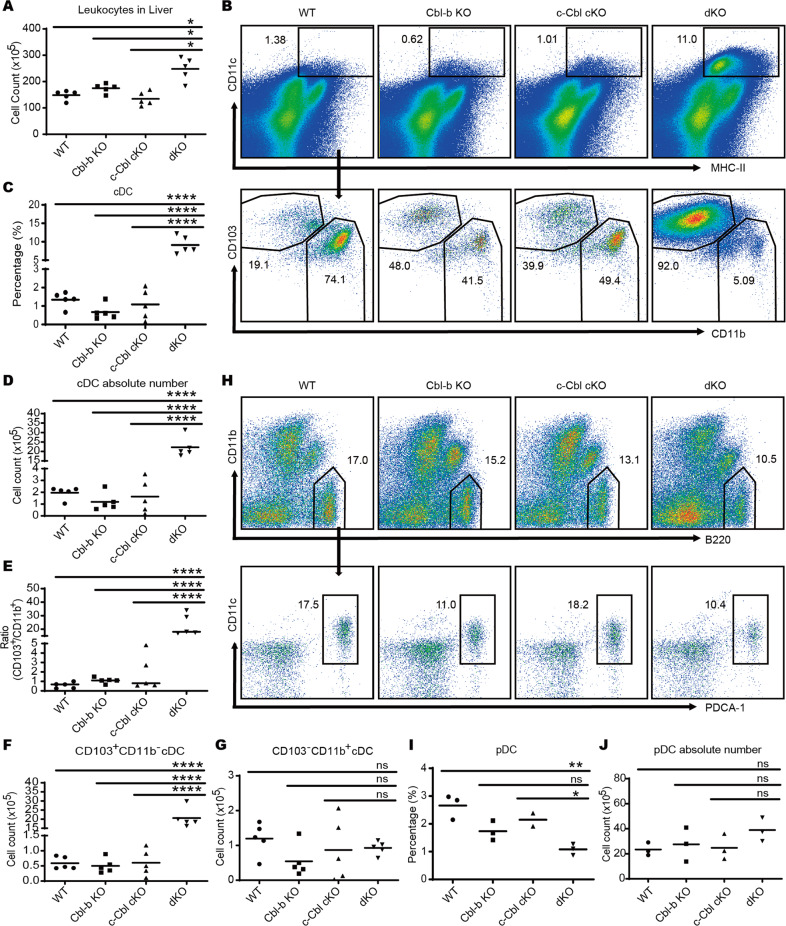
DC deficiency of Cbl-b and c-Cbl leads to hepatic cDC1 cell accumulation. **A** Absolute number of leukocytes in the liver. *n* = 5 per group. **B** Liver leukocytes were isolated and cDC subsets were analyzed by flow cytometry after staining for MHC-II, CD11c, CD11b, and CD103. *n* = 5 per group. The percentage (**C**) and absolute number (**D**) of cDCs was higher in the liver of dKO mice than in the other three groups. *n* = 5 per group. **E** The ratio of CD103^+^CD11b^−^/CD103^−^CD11b^+^ cDCs was elevated in the liver of dKO mice. *n* = 5 per group. The absolute number of CD103^+^CD11b^−^ cDC1s (**F**) and CD103^−^CD11b^+^ cDC2s (**G**) in the liver. *n* = 5 per group. **H** Liver leukocytes were isolated and subsets of pDCs were analyzed by flow cytometry following staining with B220, CD11c, CD11b, and PDCA-1. *n* = 3 per group. The percentage (**I**) and absolute number (**J**) of pDCs in the liver. *n* = 3 per group. *p < 0.05, **p < 0.01, ****p < 0.0001, ns, no significance, One-Way ANOVA comparisons. *p* < 0.05 was considered statistically significant.

As only dKO mice developed liver fibrosis and cirrhosis, we speculated that certain functional aspects of dKO DCs might be altered relative to other three genotypes which enabled either direct or indirect mediation of liver impairment. To verify this, we analyzed DC compartments in the liver. Consistent with the results of the H&E staining, we found a much larger number of infiltrating leukocytes in the livers of dKO mice compared to the other three genotypes (Fig. 2A). The percentage and absolute number of CD11c^high^ MHC-II^+^ cDCs were more than 10-fold higher in the dKO liver (Fig. 2B–D), than in WT, Cbl-c cKO, or Cbl-b KO mice. Further analysis of cDC subsets in the liver showed that the ratio of cDC1s (CD103^+^CD11b^−^) to cDC2s (CD103^−^CD11b^+^) was higher in the liver of dKO mice than in the other groups (Fig. 2E). Surprisingly, the absolute number of cDC1s was nearly 40-fold higher in dKO livers than in WT livers (Fig. 2F) while the absolute number of cDC2s showed no significant difference between the four groups of mice (Fig. 2G). The absolute pDC numbers were similar between the four groups of mice although the percentage was decreased significantly due to the increase in cDC absolute number (Fig. 2H–J). These data indicated an accumulation of cDC1s in the dKO mice which contributed to the accumulation of cDCs in the liver. Inspection of other organs including spleen (Fig. S2) and bone marrow (Fig. S3) revealed a similar elevation of cDC1s/cDC2s, indicating Cbl-b and c-Cbl may affect the homeostasis of systemic cDC1s.

### Impaired apoptosis and hyper-proliferation of dKO cDC1s contribute to the accumulation of cDC1s in mice

**Fig. 3:**
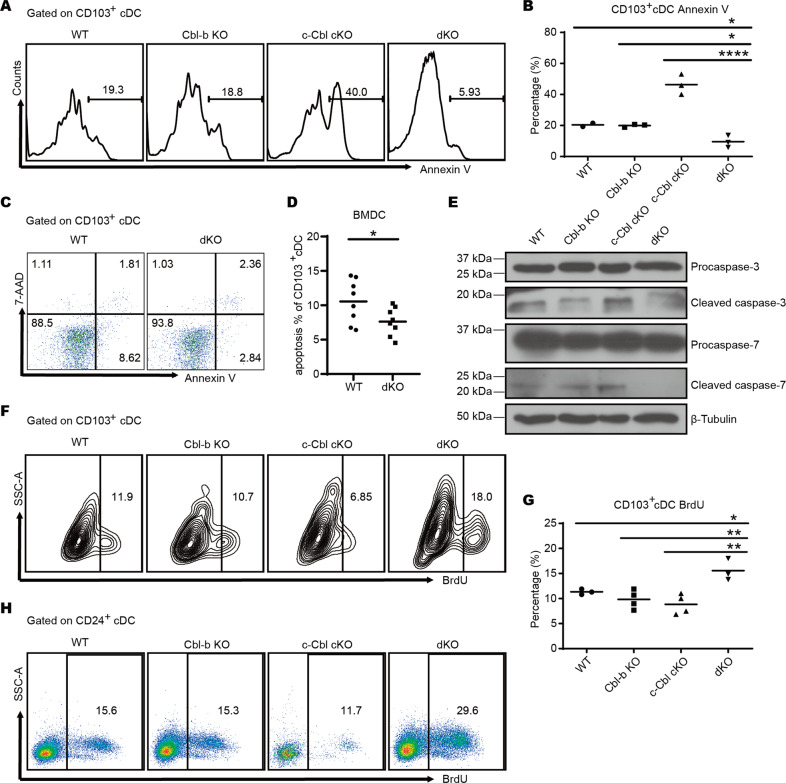
Ablation of Cbl-b and c-Cbl decreases the apoptosis and increases the proliferation of cDC1s. **A** Leukocytes isolated from livers were stained with surface markers, subjected to Annexin V staining, and assessed with flow cytometry. *n* = 3 per group. **B** The percentage of Annexin V^+^ CD103^+^ cDC1s in liver. **C**, **D** Bone marrow cells of WT and dKO mice were cultured with 20 ng/ml Flt3-L for 7 days and 20 ng/ml Flt3-L plus 2 ng/ml GM-CSF for another 2 days. The percentage of apoptotic cells was determined by flow cytometry. *n* = 8 per group. **E** CD103^+^ cDCs (MHC-II^+^ CD11c^+^) differentiated in (**C**) were sorted and the protein expression of caspase-3 and caspase-7 was measured by Western blot. **F** Mice were injected with 1 mg BrdU 24 h and 6 h prior to sacrifice, respectively. Proliferation of liver cDCs was quantified using flow cytometry. *n* = 4 per group. **G** The percentage of BrdU^+^ CD103^+^ cDC1s in the liver. **H** Bone marrow cells of WT and dKO mice were cultured as in (**C**) followed by 10 µM BrdU incubation for 6 h and quantification via flow cytometry. *p < 0.05, **p < 0.01, ****p < 0.0001, ns, no significance, One-Way ANOVA comparisons for **B** and **G**, unpaired Student’s *t* test for (**D**). *p* < 0.05 was considered statistically significant.

To investigate the reasons for the accumulation of cDC1s in Cbl-b and c-Cbl dKO mice, we initially assessed levels of apoptosis. As expected, apoptotic CD103^+^ cDC1s in the livers of dKO mice was significantly reduced compared to WT mice (Fig. 3A, B). Next, we utilized Flt3-L and GM-CSF to differentiated bone marrow cells, by which develop a high proportion of cDC1s [18], and assessed apoptosis in vitro via Annexin V staining. Consistent with our in vivo results, the proportion of apoptotic cells was significantly decreased in dKO CD103^+^ cDC1s compared with WT cells (Fig. 3C, D). We further confirmed this result by Western blotting analysis of the cleavage of two effector caspases of apoptosis, caspase-3 and caspase-7. Consistently, caspase-3 and caspase-7 cleavage were found to be much lower in protein extracts from dKO CD103^+^ cDC1s than in WT extracts, with no change in levels of the uncleaved versions of these molecules (Fig. 3E). These data indicated that Cbl-b and c-Cbl deficiency inhibited cDC1 apoptosis which in turn might contribute to an accumulation of these cells.

We also analyzed cell proliferation and development. We found that the percentage of BrdU^+^ proliferative cells both in dKO liver and BM-derived cDC1s was increased compared to WT (Fig. 3F–H), indicating a deficiency of both Cbl-b and c-Cbl enhanced cDC1 proliferation. However, the percentages of cDC precursors/progenitors, including macrophage DC progenitors (MDP) (Fig. S4A, B), common DC progenitors (CDP) (Fig. S4C, D), pro-DCs (Fig. S4E, F), and pre-DCs (Fig. S4G, H) which are intermediate precursors of cDCs that lost their potential to differentiate into pDCs, were similar across the four genotypes of mice, indicating that Cbl-b and c-Cbl did not affect DC development.

### dKO cDC1s contribute to liver fibrosis and cirrhosis

**Fig. 4:**
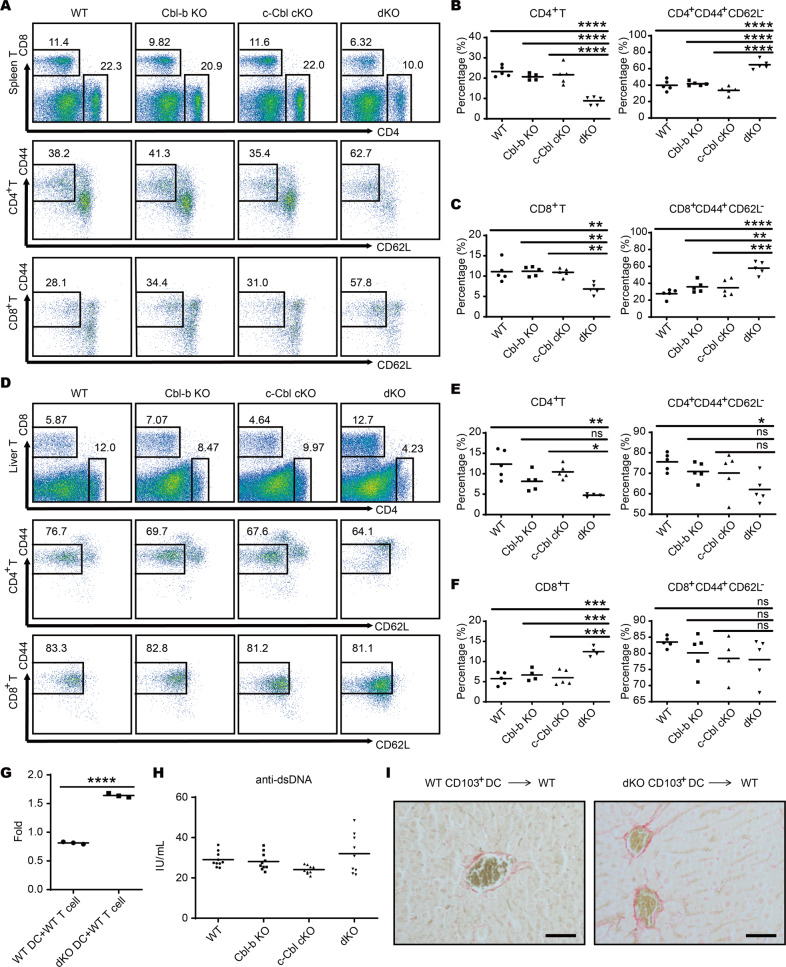
Cbl-b and c-Cbl inhibit the T cell priming ability of cDC1s. **A** The surface expression of CD44 and CD62L on CD4 and CD8 T cells in the spleen detected by flow cytometry. *n* = 5 per group. **B** The percentage of CD4^+^ T cells (left) and activated (CD44^+^CD62L^−^) CD4^+^ T cells (right) in the spleen. **C** The percentage of CD8^+^ T cells (left) and activated (CD44^+^CD62L^−^) CD8^+^ T cells (right) in the spleen. **D** The surface expression of CD44 and CD62L on CD4 and CD8 T cells in the liver detected by flow cytometry. *n* = 5 per group. **E** The percentage of CD4^+^ T cells (left) and activated (CD44^+^CD62L^−^) CD4^+^ T cells (right) in the liver. **F** The percentage of CD8^+^ T cells (left) and activated (CD44^+^CD62L^−^) CD8^+^ T cells (right) in the liver. **G** BM-derived cDC1s of WT and dKO mice loaded with VSV antigen were co-cultured with T cells isolated from WT VSV-immunized mice, and the proliferation of T cells was measured by Cell Counting Kit 8. *n* = 3 per group. **H** Serum of mice aged 5–6 months was collected and anti-dsDNA antibodies were quantified using ELISA (*n* = 10 per group). **I** Hepatic CD103^+^ cDCs purified from WT or dKO mice were adoptively transferred into WT recipient mice respectively and liver Sirius red staining was conducted; scale bar, 100 μm. Data are combined from three independent experiments. *p < 0.05, **p < 0.01, ***p < 0.001, ****p < 0.0001, ns, no significance based on One-Way ANOVA comparisons for **B**, **C**, **E**, **F** and **H** and unpaired Student’s *t* test for (**G**). *p* < 0.05 was considered statistically significant.

Next, we wanted to identify the mechanisms underlying liver fibrosis and cirrhosis in dKO mice. We speculated that dKO cDC1s may exhibit a higher potency for T cell priming, which could lead to T cell hyperactivation and the breakdown of immune tolerance. To test this hypothesis, we evaluated T cell activation via flow cytometry measurement of CD44^+^ and CD62L^−^ cells, two markers for T cell activation. As expected, the percentages of CD4^+^CD44^+^CD62L^−^ and CD8^+^CD44^+^CD62L^−^ T cells were increased in spleens of dKO mice compared with the other three groups (Fig. 4A–C). However, T cell activation in liver was not significantly altered (Fig. 4D–F). To investigate the direct effects of dKO cDC1s on T cell activation, we loaded BM-derived CD103^+^ cDC1s with VSV antigen and subsequently co-cultured them with T cells isolated from WT VSV-immunized mice. Consistent with our in vivo results, T cells that were co-cultured with dKO CD103^+^ cDC1s exhibited higher proliferation than cells co-cultured with WT CD103^+^ cDC1s (Fig. 4G). Conversely, we did not observe significant changes in the expression of activation markers CD80 and CD86 in liver B cells (Fig. S5A–F) or the production of autoantibodies (Fig. 4H). Taken together, these data suggested that DC deficiency of both Cbl-b and c-Cbl only partially disrupted immune quiescence.

The participation of hepatic DCs in liver inflammation and fibrosis via several mechanisms has been suggested by several studies using fibrogenic models induced by thioacetamide [19], carbon tetrachloride [20] or bile duct ligation [21]. To explore whether dKO CD103^+^ cDC1s could directly drive liver fibrosis, we conducted an adaptive transfer of hepatic CD103^+^ cDC1s purified from WT or dKO mice into WT recipient mice, respectively. This revealed an incomplete hepatocyte barrier around the blood vessels of recipient mice transferred with dKO CD103^+^ hepatic cDC1s, indicating early symptoms of liver fibrosis (Fig. 4I). These data suggested that dKO CD103^+^ cDC1s could directly contribute to liver fibrosis and cirrhosis.

### dKO CD103 + cDC produces more proinflammation cytokines

Hepatic fibrosis and cirrhosis typically follows chronic inflammation [20, 22]. To figure out whether pro-inflammation cytokines participate in Cbl-b and c-Cbl deleted DCs induced liver cirrhosis, we detected the expressions of certain cytokines involved in liver cirrhosis. qPCR results showed that ILs (including IL-1β, IL-4, IL-10, IL-6, IL-12, and IL-17A) were markedly up-regulated in dKO BM-derived CD103^+^ cDC but not TNF-α, TGF-β, and IFN-γ compared with WT mice (Fig. S6A–I), indicating the participating of both pro-inflammation and anti-inflammation cytokines in liver cirrhosis but the changes of pro-inflammation cytokines in dKO mice were more vigorous than anti-inflammation cytokines which might participate in the impairment of liver function.

To elucidate the functional roles of cytokines IL-1, IL-6, IL-12, and IL-17A, we crossed IL-1R^−/−^, IL-6^−/−^, IL-12^−/−^ and IL-17A^−/−^ mice with our dKO mice to generate mice lacking expression of Cbl-b, c-Cbl both proteins with ILs mentioned above and the survival time of these mice were recorded. Excitingly, IL-1R, IL-6, or IL-17A, but not IL12 deletion in dKO mice respectively partly prolonged the survival time of dKO mice (Fig. S6J) which means that production of pro-inflammation cytokines is responsible for the nosogenesis, at least partly, of liver cirrhosis.

### Cbl-b and c-Cbl promote mitochondria-dependent apoptosis via downregulation of STAT5 and subsequent depression of Bim

**Fig. 5:**
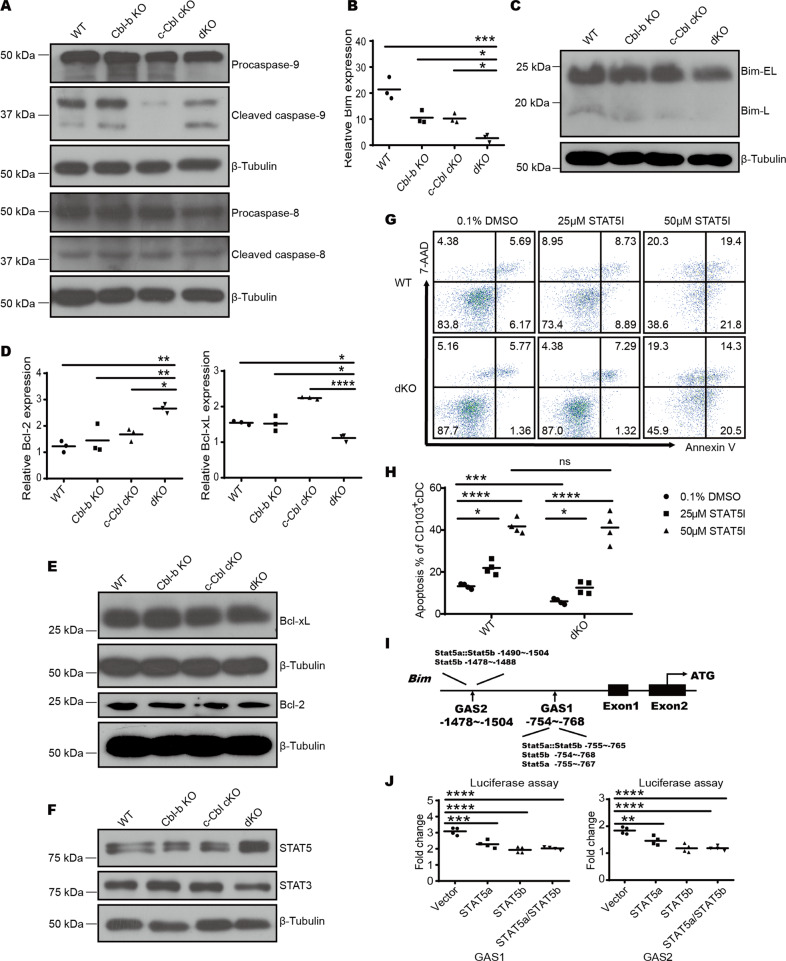
Cbl-b and c-Cbl regulate mitochondria-dependent apoptosis of CD103^+^ cDCs via the STAT5-Bim signaling pathway. **A** CD103^+^ cDCs (MHC-II^+^CD11c^+^) differentiated using 20 ng/mL Flt3-L and 2 ng/mL GM-CSF were sorted and protein levels of caspase-8 and caspase-9 were measured by Western blot. **B** Quantitative RT-PCR analysis of Bim mRNA levels in BM-derived CD103^+^ cDCs. *n* = 3 per group. **C** Western blot analysis of Bim expression in BM-derived CD103^+^ cDCs. **D** Quantitative RT-PCR analysis of Bcl-2 and Bcl-xL mRNA levels in BM-derived CD103^+^ cDCs. *n* = 3 per group. **E** Western blot analysis of Bcl-2 and Bcl-xL expression levels in BM-derived CD103^+^ cDCs. **F** Western blot analysis of STAT5 and STAT3 expression levels in BM-derived CD103^+^ cDCs. **G** STAT5-IN-1 was added to CD103^+^ cDC cultures at the indicated concentration for 9 days after which apoptosis levels were analyzed by flow cytometry. *n* = 4 per group. **H** The percentage of Annexin V^+^ 7AAD^−^ CD103^+^ cDCs treated with the indicated concentration of the STAT5 inhibitor. **I** Schematic of the Bim gene. Vertical boxes indicate exons and the location of conserved GAS sequences is shown. **J** Luciferase assays of STAT5 binding to the putative GAS sites. *n* = 4 per group. *p < 0.05, **p < 0.01, ***p < 0.001, ****p < 0.0001, ns, no significance based on One-Way ANOVA comparisons. *p* < 0.05 was considered statistically significant.

Apoptosis is mainly triggered by two pathways, namely the exogenous pathway initiated by the death receptor and the endogenous mitochondria-dependent pathway, where initiator caspase-8 and caspase-9 are cleaved and activated [23]. To investigate how Cbl-b and c-Cbl can influence apoptosis in cDC1s, we evaluated the expression of caspase-8 and caspase-9 cleavage in BM-derived CD103^+^ cDC1s induced with Flt3-L and GM-CSF by Western blotting. Caspase-9, but not caspase-8, exhibited significantly reduced cleavage in dKO CD103^+^ cDCs (Fig. 5A) with no change in their uncleaved forms, indicating Cbl-b and c-Cbl are involved in a mitochondria-dependent apoptosis of CD103^+^ cDC1s.

In the endogenous apoptosis pathway, the integrity of the mitochondrial membrane and apoptosis are determined by the balance between pro- and anti-apoptotic Bcl-2 family members [24]. Hence, we quantified the expression of Bcl-2 family members at both protein and mRNA levels. Both protein and mRNA levels of Bim were lower in dKO CD103^+^ cDC1s (Fig. 5B, C) while the expression levels of other anti-apoptotic members, including Bcl-2 and Bcl-xL, were similar among four genotypes (Fig. 5D, E). Given that previously published reports suggested that Bim could control DC apoptosis [8], these data suggested that Cbl-b and c-Cbl promoted cDC1 apoptosis by upregulation of Bim.

Considering that Bim mRNA levels were lower in dKO CD103^+^ cDC1s than in WT CD103^+^ cDC1s, we hypothesized that Cbl-b and c-Cbl may influence Bim expression at transcriptional level and aimed to identify potentially relevant transcriptional factors. A previously published ChIP-Seq study revealed that STAT3 and STAT5 compete for binding to the Bcl2l11 promotor, which encodes Bim, to promote and inhibit Bcl2l11 transcription and cell apoptosis in cDCs [16]. In agreement with this data, we found that STAT5 protein levels were significantly increased while STAT3 levels were decreased slightly in dKO CD103^+^ cDCs compared to the other three genotype groups (Fig. 5F), indicating Cbl-b and c-Cbl downregulated the expression of STAT5. To verify the inhibitory role of STAT5 in apoptosis of cDC1s, we applied a specific STAT5 inhibitor to our BM-derived CD103^+^ cDC culture system and quantified apoptosis by flow cytometry. Treatment with the STAT5 inhibitor increased the percentage of apoptotic WT CD103^+^ cDCs in a dose-dependent manner, with differences in apoptosis levels between WT and dKO CD103^+^ cDCs leveling off at 50 µM (Fig. 5G, H). These data suggested that STAT5 indeed participated in cDC1 apoptosis under the control of Cbl-b and c-Cbl.

To confirm the binding of STAT5 to the Bcl2l11 promoter, we used JASPAR (http://jaspar.genereg.net/) to predict the binding sequences (GAS) [25] and selected five sites with the highest score for a binding assay. The selected sites were distributed across two segments, GAS1 and GAS2 (Fig. 5I). Firefly luciferase reporter plasmids that containing one of the two segments were co-transfected with STAT5a- or STAT5b-overexpressing plasmids or both, and a control plasmid expressing Renilla luciferase into HEK293T cells. The activity of firefly luciferase relative to Renilla luciferase was reduced in the presence of predicted binding sites and STAT5a/b, indicating STAT5 could bind to Bcl2l11 promoter (Fig. 5J), which subsequently led to the repression of gene expression and further inhibition of DC apoptosis.

### Cbl-b and c-Cbl target STAT5 for ubiquitination at multiple lysine sites

**Fig. 6:**
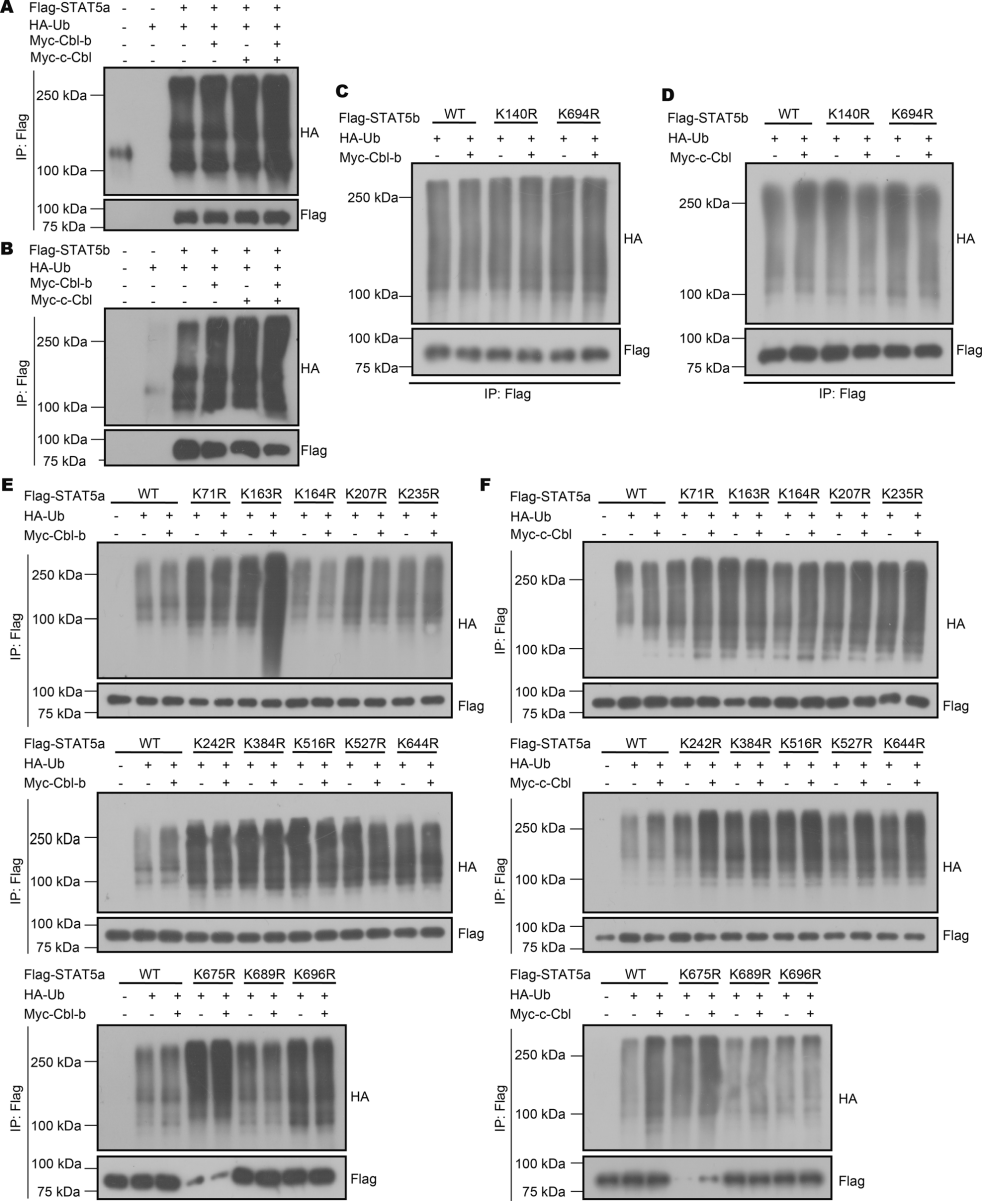
Cbl-b and c-Cbl ubiquitinate different lysine sites of STAT5. The ubiquitination of STAT5a (**A**) and STAT5b (**B**) by Cbl-b and c-Cbl in HEK293T cells. **C** Immunoprecipitation analysis of ubiquitination of STAT5b by Cbl-b in HEK293T cells transfected with Flag-STAT5b (WT or mutant). **D** Immunoprecipitation analysis of linear ubiquitination of STAT5b by c-Cbl in HEK293T cells transfected with Flag-STAT5b (WT or mutant). **E** Immunoprecipitation analysis of linear ubiquitination of STAT5a by Cbl-b in HEK293T cells transfected with Flag-STAT5a (WT or mutant). **F** Immunoprecipitation analysis of linear ubiquitination of STAT5a by c-Cbl in HEK293T cells transfected with Flag-STAT5a (WT or mutant). Data are pooled from three independent experiments.

As members of the Cbl family are E3 ubiquitin ligases, we speculated that Cbl-b and c-Cbl might directly regulate STAT5 via ubiquitination. To verify this hypothesis, we co-transfected overexpression plasmids for STAT5a, STAT5b, Cbl-b, and c-Cbl into HEK293T cell lines. Cbl-b and c-Cbl facilitated STAT5 ubiquitination (Fig. 6A, B), indicating that the increased expression of STAT5 in dKO CD103^+^ cDCs may result from the deficiency of Cbl-b and c-Cbl.

Based on the results above, we then continued to investigate potential ubiquitination types and sites of both STAT5a and STAT5b mediated by Cbl-b and c-Cbl. Using the Protein Lysine Modification Database, we identified thirteen potential ubiquitinated lysine (Lys) residues on STAT5a, including Lys71, 163, 164, 207, 235, 242, 384, 516, 527, 644, 675, 689, and 696, and two potential ubiquitinated lysine (Lys) residues on STAT5b, including Lys140 and 694. We then mutated each potential ubiquitinated lysine (K) residue to arginine (R) and then co-transfected the mutant STAT5a or b-overexpressing plasmids into HEK293T cells with Cbl-b or c-Cbl-overexpressing plasmids. Immunoprecipitation assays showed that c-Cbl mediated both K140 and K694 ubiquitination on STAT5b, as K140 and K694 mutation reduced c-Cbl mediated ubiquitination (Fig. 6C). Conversely, Cbl-b did not appear to mediate STAT5b ubiquitination (Fig. 6D). In addition, both Cbl-b and c-Cbl mediated STAT5a ubiquitination, but acted on different sites. Cbl-b preferentially ubiquitinated the K164 (Fig. 6E) while c-Cbl ubiquitinated the K696 residue of STAT5a (Fig. 6F). These results indicated different ubiquitination sites of Cbl-b and c-Cbl on STAT5.

**Fig. 7:**
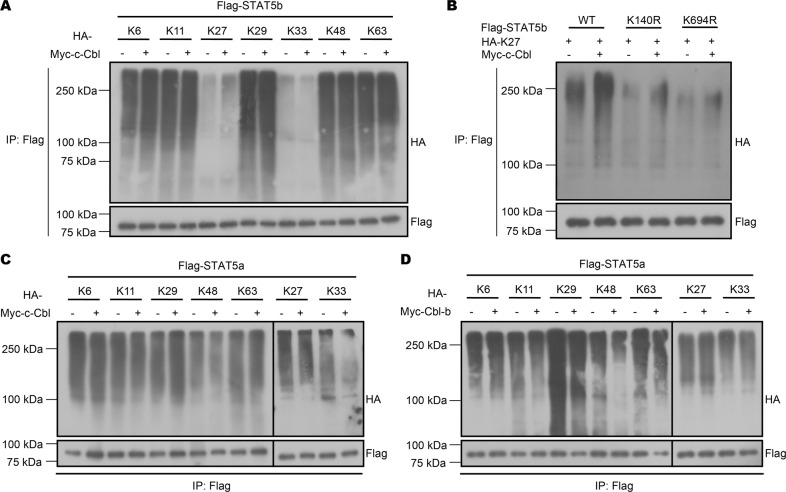
Cbl-b and c-Cbl mediate K27- and K29-linked ubiquitination of STAT5a/b. **A** Immunoprecipitation analysis of ubiquitination types of STAT5b by c-Cbl in HEK293T cells co-transfected with Flag-STAT5b, Myc-c-Cbl, and different types of HA-Ub. **B** Immunoprecipitation analysis of K27-linked ubiquitination of STAT5b (WT or K140R/K694R) by c-Cbl in HEK293T cells. **C** Immunoprecipitation analysis of ubiquitination types of STAT5a by c-Cbl in HEK293T cells co-transfected with Flag-STAT5a, Myc-c-Cbl, and different types of HA-Ub. **D** Immunoprecipitation analysis of ubiquitination types of STAT5a by Cbl-b in HEK293T cells co-transfected with Flag-STAT5a, Myc-Cbl-b. and different types of HA-Ub. Data are pooled from three independent experiments.

We next employed different types of ubiquitin, including K6, K11, K27, K29, K33, K48, and K63, each of which harbored only one corresponding lysine on ubiquitin. We found that c-Cbl mediated K27-linked K140 and K694 ubiquitination of STAT5b, as mutation of K140 and K694 prevented K27-linked poly-ubiquitination of STAT5b (Fig. 7A, B). Consistently, we found that the type of ubiquitination differed between c-Cbl and Cbl-b, with c-Cbl mediating K29-linked ubiquitination (Fig. 7C) of STAT5a and Cbl-b mediating K27-linked ubiquitination (Fig. 7D). In conclusion, our results demonstrated that Cbl-b and c-Cbl may synergistically influence STAT5 modification via distinct ubiquitination types and sites.

## Discussion

Here, we found that simultaneous deletion of c-Cbl and Cbl-b in CD11c^+^ cells led to an accumulation of cDC1s in lymphoid and non-lymphoid tissues via increased proliferation and reduced apoptosis. As the percentage of cDC1s in Cbl-b or c-Cbl single knockout mice did not change, we hypothesized that Cbl-b and c-Cbl have overlapping functions in regulating cDC1 cell proliferation and apoptosis. Basal expression levels of cytokines such as IL-6 and IL-12 remained similar in Cbl-b or c-Cbl single knockout BMDCs with WT BMDC, but was higher in dKO cells (Fig. S6B, C), indicating a synergistic or partially redundant role of Cbl-b and c-Cbl in regulation of DC activation.

DC maturation and activation state is a critical determinant for the outcome of the interaction between T cells and DCs [26–28]. Here we observed an enhanced T cell activation in dKO mice. One explanation for this could be that dKO DC activation was enhanced. However, emerging evidence shows that DC apoptosis/survival also constitutes a key factor for T cell fate during T-DC interactions [29, 30]. Any defects in DC apoptosis, whether extrinsic or intrinsic, can lead to enhanced T cell activation, and in more serious cases the breakdown of T and (or) B cell tolerance [7, 8, 31]. Therefore, decreased apoptosis of dKO cDC1s may be another contributor to enhanced T cell activation in dKO mice. Chen et al. reported that Bim^−/−^ DCs induced more robust T-cell activation both in vitro and in vivo, and even induced autoantibody production after adoptive transfer [8]. However, possibly because the decrease in Bim expression in dKO cDC1s was rather mild, we found that T cell quiescence in dKO mice was partially destroyed, and B cell activation and autoantibody production in serum remained unchanged, indicating that deficiency of both Cbl-b and c-Cbl in DCs only partially disrupted immune quiescence.

Liver fibrosis results from chronic damage to the liver and is a characteristic of most types of chronic liver diseases including autoimmune hepatitis, alcohol-induced liver degeneration, hepatitis C infection, and non-alcohol-induced steatohepatitis. The roles of immune cells including macrophages [32], T [33], B [34], NKT, and NK cells [35] in liver fibrosis and cirrhosis have been well established. However, to the best of our knowledge, there are only few published studies showing the possible involvement of DCs in fibrogenesis [19–21]. Here, we found cDC1s accumulated in livers of dKO mice that developed liver fibrosis and cirrhosis. However, using different mouse models of liver fibrosis, Connolly et al. [19] and Bleier et al. [21] previously found a significant accumulation of CD11b^+^ cDC2s.

Liver fibrosis in dKO mice was autoimmune-unrelated as dKO mice did not manifest autoimmune disorders, which was also confirmed by data using Rag1^−/−^ dKO mice model [36]. Although macrophages and NK cells are involved in liver fibrosis, we did not observe an increase in the numbers of liver infiltrating NK cells and macrophages [36]. Adoptive transfer result indicated that dKO cDC1s could directly initiate or drive liver fibrosis, possibly by creating an inflammatory environment due to their massive hepatic accumulation and elevated basal expression of inflammatory cytokines. Reducing the levels of inflammatory cytokines in dKO mice by crossing these mice with mice deficient in certain cytokine prolonged their lifespan (Fig. S6J), indicating that inflammatory cytokines indeed contributed to the promotion of liver fibrosis. Thus, our data suggested that the liver inflammation in dKO mice was positively correlated with CD103^+^ cDC1s and provides direct evidence for the fact that inflammatory cDC1s may contribute to the development of liver fibrosis.

Eddy et al. found a significant reduction in CD103^+^ DCs, but not CD11b^+^ DCs, in lungs of STAT5-deficient mice compared with WT mice. However, the underlying mechanisms for this reduction were unknown [37]. Wan et al. found that GM-CSF-induced STAT5 activation could reverse IL-21-induced apoptosis of cDCs by competing with STAT3 for binding of Bim [16]. Here, we demonstrated that STAT5 promoted CD103^+^ DC survival by negatively regulating Bim expression. To the best of our knowledge, our study is the first to investigate the ubiquitination sites and types of STAT5 regulated by Cbl-b and c-Cbl, elucidating the relationship of DCs apoptosis with its immune actions and the mechanisms of DCs in liver functional regulation maybe helpful for liver diseases therapy.

## Materials and methods

### Mice

Mice were C57BL/6 background. c-Cbl^flox/flox^ and Cbl-b^−/−^ mice were kindly gifted by Dr. Hua Gu (Montreal Clinic Research Institute, Montreal, Quebec, Canada) [12]. CD11c-Cre mice were gifted by Prof. Boris Reizis [38]. CD11c-Cre mice were crossed with c-Cbl^flox/flox^ to generate c-Cbl^flox/flox^ CD11c-Cre^+^ mice. Subsequently, the progeny was crossed with Cbl-b^−/−^ mice to generate Cbl-b^−/−^ c-Cbl^flox/flox^ CD11c-Cre^+^ (dKO) mice. All mice were housed under specific pathogen-free conditions at the Soochow University animal facility. 6–8 weeks old mice were used unless indicated with no sex-selective preference. All animal experiments were performed following the institutional guidelines and approved by the Institutional Animal Use and Care Committee of Soochow University.

### Cell culture and VSV amplification

Human embryonic kidney (HEK) 293T and Vero cell lines were purchased from the American Type Culture Collection (ATCC) and cultured in DMEM medium (HyClone, Logan, Utah) containing 10% fetal bovine serum (Gibco, Hong Kong), 100 U/mL penicillin and 100 µg/mL streptomycin (Gibco, Hong Kong). After Vero cells reached 80% confluence, 20 µL VSV virus were added to 10 mL DMEM medium containing 5% fetal bovine serum. The supernatant was collected 4–5 days post infection and ultra-centrifugated for VSV virus concentration.

### H&E staining and hepatic Sirius red staining

Tissues were harvested, fixed, embedded in paraffin, and cut into 8 µM sections. For H&E staining, hepatic slides were stained with hematoxylin solution (Solarbio, Beijing, China) for 8 min and eosin solution (WeiAo, Shanghai, China) for 40 s. For Sirius red staining, slides were stained with Sirius red (Sigma-Aldrich, USA) for 60 min and rinsed with hydrochloric acid (0.01 M) for 1 min.

### Isolation of liver DCs

Mouse livers were cut into fine pieces and digested with 0.1 mg/mL collagenase IV (Sigma-Aldrich, USA) and 20 µg/mL DNase I (TaKaRa, Dalian, China) in RPMI-1640 medium for 30 min at 37 °C. The cells were then washed twice with ice-cold PBS and resuspended in 10 mL Mouse 1 × Lymphocyte Separation Medium (DAKEWE, China). Lymphocytes were separated according to the manufacturer’s protocol.

### Cell staining and flow cytometry

Cells were stained with fluorescence-coupled antibodies listed below diluted in PBS for 20 min on ice and subsequently washed with PBS. Analysis was carried out on a BD Cyan (BD Biosciences, USA) while cell sorting was conducted on a FACS Aria III (BD Biosciences, USA). FITC-anti-B220 (RA3-6B2) (#103206), APC/Cy7-anti-CD11c (N418) (#117324), FITC-anti-MHC-II (AF6-120.1) (#116406), PE-anti-CD11b (M1/70) (#101208), APC-anti-CD103 (2E7) (#121414), APC-anti-CD24 (clone M1/69) (#101814), PE/Cy7-anti-CD172α (P84) (#144008), APC-anti-PDCA-1 (927) (#127016), PE/Cy7-anti-CD80 (16-10A1) (#104734), PE-anti-CD86 (GL-1) (#105008), FITC-anti-CD4 (GK1.5) (#100406), APC-anti-CD8 (53-6.7) (#100712), PE-anti-CD44 (IM7) (#103024), and APC/Cy7-anti-CD62L (MEL-14) (#104428) were purchased from BioLegend (San Diego, USA). PE/Cy7-anti-CD11b (M1/70) (#552850) and PE-anti-BrdU (3D4) (#556029) were purchased from BD Pharmingen (USA). For BrdU incorporation analysis in vivo, mice were intraperitoneally injected with 1 mg BrdU in PBS at 12 h and 4 h before sacrifice. Cells were stained with corresponding antibodies against surface markers and BrdU incorporation was measured using an anti-BrdU Kit according to the manufacturer’s protocols (BD Pharmingen^TM^ BrdU Flow Kit) (#557891). For BrdU incorporation analysis in vitro, cells were incubated in 10 µM BrdU for 6 h and subsequently analyzed by flow cytometry. For apoptosis analysis, cells were first stained with corresponding antibodies against surface markers and subsequently stained with Annexin V and 7-AAD according to the manufacturer’s protocols (BD, Franklin Lakes, NJ, USA).

### BMDC preparation and cell culture

Bone marrow cells were harvested from mouse femurs and tibias. Erythrocytes were removed using a hypotonic solution. Cells were cultured at a density of 10^6^ cells/mL in RPMI-1640 medium (HyClone, Logan, Utah) containing 10% fetal bovine serum, 100 U/mL penicillin, 100 µg/mL streptomycin, and 20 ng/mL Flt3-L (Peprotech) for 9 d. Half of the medium was exchanged every 3 d. At day 7, 2 ng/mL GM-CSF (Peprotech) were added. At day 9, cells were harvested for analysis or CD103^+^ cDC isolation. For STAT5 inhibitor treatment, different concentrations of the STAT5 inhibitor MCE (STAT5-IN-1) were added to the culture system for 9 d. The inhibitor was replenished with media exchanges. For western blot analysis of Cbl-b and c-Cbl expression in CD11c-positive DCs, bone marrow cells were cultured at a density of 10^6^ cells/mL in RPMI-1640 medium containing 10% fetal bovine serum, 100 U/mL penicillin, 100 µg/mL streptomycin, and 10 ng/mL GM-CSF (Peprotech) for 7 d, with half of the medium being replaced every 3 days, followed by isolation using CD11c-positive magnetic beads.

### Adoptive transfer mouse model

Two-week-old Cbl-b^+/+^ c-Cbl^flox/flox^ CD11c-Cre^-^ mice in a C57BL/6 background were irradiated and injected intravenously with flow cytometry-sorted single cell suspensions of WT or dKO hepatic CD103^+^ cDCs (2 × 10^5^ cells). Injections were carried out 8 times, once every 2 weeks. After 18 weeks, mice were sacrificed to assess pathologic liver changes via Sirius red staining.

### In vitro T cell activation assay

WT mice received intramuscular injection of 100 µL VSV virus at a titer of 10^−7^ TCID_50_. After 7 days, virus-infected WT mice were sacrificed to prepare a splenic single cell suspension. T cells were sorted using immuno-magnetic beads and counted. CD103^+^ cDCs differentiated from the bone marrow of WT or dKO mice were sorted by flow cytometry and incubated with VSV at 37 °C for 24 h. After incubation, cells were centrifuged at 1000–1200 rpm at 4 °C for 10 min and washed twice with serum-containing medium to remove residual virus. The number of cells was adjusted to co-culture 5 × 10^4^ CD103^+^ cDCs of WT or dKO mice with 5 × 10^5^ WT T cells in each well of a 96-well plate at a total volume of 100 µL. After 4 d of co-culture, 10 µL CCK8 solution were added to each well and the OD450 nm was measured every hour for 4 h.

### Western blotting, immunoprecipitation, and ubiquitination assays

Cells were lysed in NP-40 lysis buffer (50 mM Tris, pH7.4, 150 mM NaCl, 1% NP-40, sodium pyrophosphate, β-glycerophosphate, sodium orthovanadate, sodium fluoride, EDTA, leupeptin) containing 1 mM PMSF (phenylmethanesulfonyl fluoride) and immunoblotting was conducted following standard procedures. For immunoprecipitation and ubiquitination assays, cell lysates were incubating with corresponding antibodies at 4 °C overnight followed by incubation with Protein G agarose beads (Roche Diagnostics GmbH, Mannheim, Germany) for another 4–6 h at 4 °C. Immunoprecipitates were washed four times with NP-40 wash buffer and boiled in 30 µL loading buffer for 10 min for Western blot analysis. The following antibodies were used in our study: anti-Cbl-b (D3C12) (#9498), anti-c-Cbl (D4E10) (#8447), anti-caspase-3 (D3R6Y) (#14220), anti-caspase-7 (D2Q3L) (#12827), anti-caspase-8 (1C12) (#9746), anti-caspase-9 (C9) (#9508), anti-cleaved caspase-3 (Asp175) (#9661), anti-cleaved caspase-7 (D198) (#9491), anti-cleaved caspase-8 (clone D5B2) (#8592), anti-cleaved caspase-9 (D8I9E) (#20750), anti-Bim (C34C5) (#2933), anti-STAT3 (D3Z2G) (#12640), anti-STAT5 (D206Y) (#94205), anti-Bcl-2 (D17C4) (#3498), anti-Bcl-xL (54H6) (##2764), anti-Myc (9B11) (#2276) and anti-HA (C29F4) (#5017), all purchased from Cell Signaling Technology (Danvers, MA, USA). Anti-Flag and anti-β-actin antibodies were purchased from Affinity Biosciences (USA). Anti-β-tubulin antibody was purchased from MesGen biotechnology (China). HRP-conjugated goat anti-rabbit and donkey anti-mouse antibodies were used as secondary antibodies.

### Real-time PCR

Total RNA was extracted from sorted CD103^+^ cDCs using the RNAiso Plus reagent (TaKaRa, Dalian, China). First-strand complementary DNAs (cDNAs) were synthesized using oligo(dT)s and the Reverse Transcriptase M-MLV Kit (TaKaRa, Dalian, China). Quantitative RT-PCR was performed on a 7900H sequence detection system (Applied Biosystems, USA). Quantification of all target genes was carried out by normalizing to the control gene β-actin. The sequences of primers used in Real-time PCR are listed in Table S1. All primers were synthesized by GENEWIZ (Suzhou, China).

### Plasmids and reagents

Sequences coding for Cbl-b, c-Cbl, STAT3, STAT5a, or STAT5b were amplified from C57BL/6J mouse thymus cDNA and cloned into pcDNA3.1+ basic vector between the KpnI and XhoI sites (Cbl-b, c-Cbl, and STAT3) or pcDNA3.1- basic vector between KpnI and EcoRI sites (STAT5a and STAT5b). The sequences of primers used for cloning are shown in Table S2. Thirteen STAT5a (K71R, K163R, K164R, K207R, K235R, K242R, K384R, K516R, K527R, K644R, K675R, K689R, and K696R) and two STAT5b (K140R and K694R) mutations were generated using the PrimeSTAR^®^ GXL DNA Polymerase (TaKaRa, Dalian, China) and FastDigest DpnI (Thermo Fisher Scientific, Lithuania) according to the manufacturer’s instructions. Primers used for STAT5 mutation are shown in Table S3.

### Luciferase reporter assays

For luciferase reporter assays, sequences containing the predicted STAT5 binding sites in the promoter region of Bim were cloned into the PGL3-basic vector between the sites for KpnI and BglII. HEK293T cells were transiently transfected with PGL3, pcDNA3.1-STAT5a and/or pcDNA3.1-STAT5b and the Renilla luciferase TK vector (phRL-TK) used for normalization. Cells were harvested after 48 h and both firefly and Renilla luciferase activity were measured using a dual luciferase reporter assay system (Promega, Madison, USA) according to the manufacturer’s instructions. Primers used for the construction of plasmids containing GAS1 and GAS2 are listed in Table S2.

### Statistical analysis

Biological replicates were performed for all experiments as indicated. Prism 6 (GraphPad) was used for statistical analysis. To assess the statistical significance of different treatment groups, we used One-Way ANOVA comparisons or unpaired two-tailed Student’s *t* tests in two different treatments. For survival curve analysis, log-rank tests were performed. *indicated *p* < 0.05; **p < 0.01; ***p < 0.001, ****p < 0.0001, ns indicates not significant. *p* values less than 0.05 were considered statistically significant. Data represent three independent experiments unless indicated.

## Supplementary information

Supplemental Material

Original Data File

Original Data File

Original Data File

## Data Availability

All data generated or analyzed during this study are included in this published article and its supplementary information files.
