# Supplementary material for: Ablation of Cbl-b and c-Cbl in dendritic cells causes spontaneous liver cirrhosis via altering multiple properties of CD103+ cDC1s
Source: Cell Death Discov. 2022 Mar 30;8:142. doi: 10.1038/s41420-022-00953-2 (PMC8967913; doi:10.1038/s41420-022-00953-2)
Supplement: Supplementary file 1 — Supplemental Material [file 41420_2022_953_MOESM1_ESM.docx]

**Supplementary information**


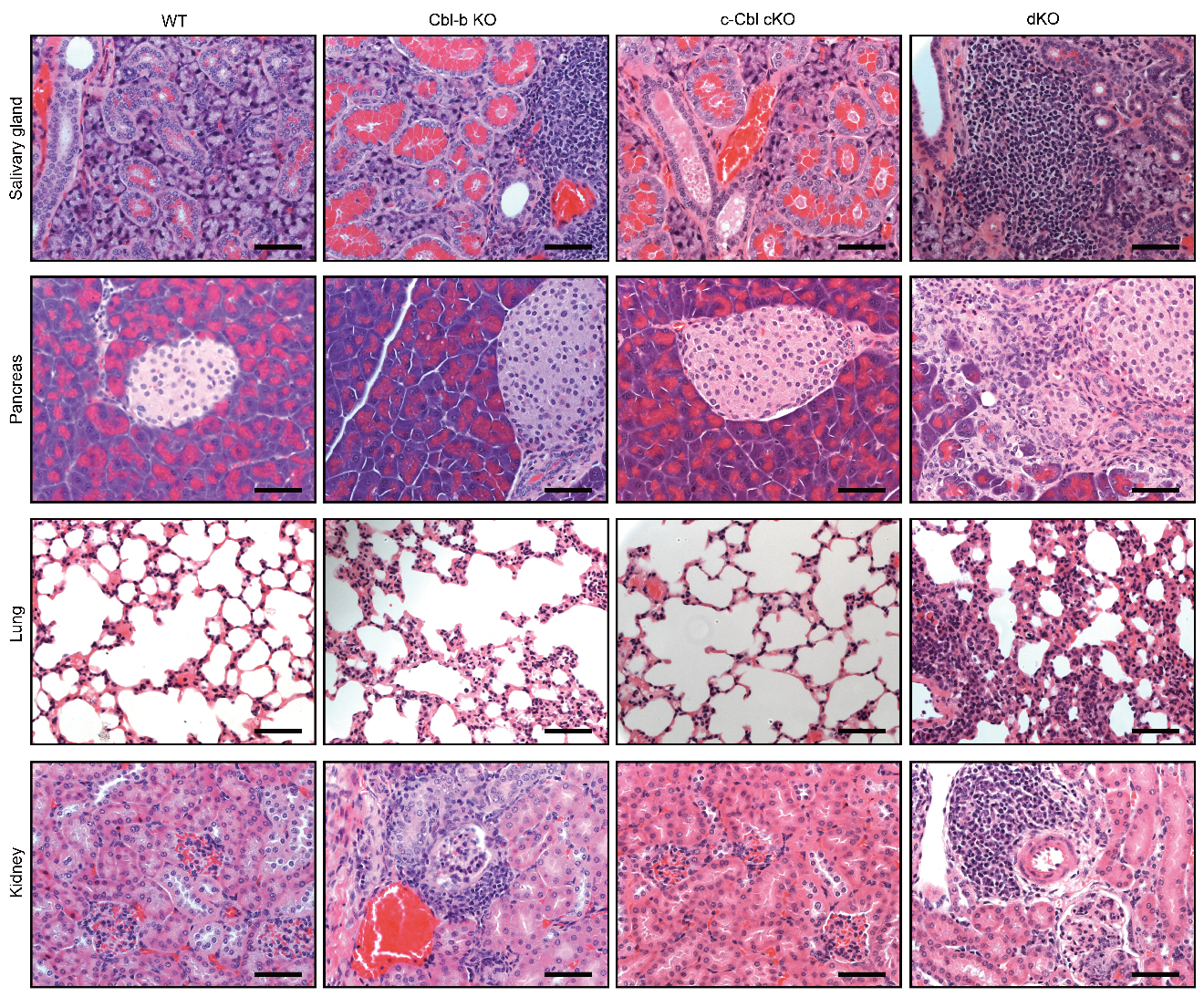


**Supplementary Figure. 1 Hematoxylin and eosin (H&E) stained sections of multiple organs of four kinds of mice**. Indicated tissues were removed and fixed with 10% formalin, and paraffin sections were stained with hematoxylin and eosin (n=3 per group); scale bars 50 μm. Data represent three independent experiments.

**
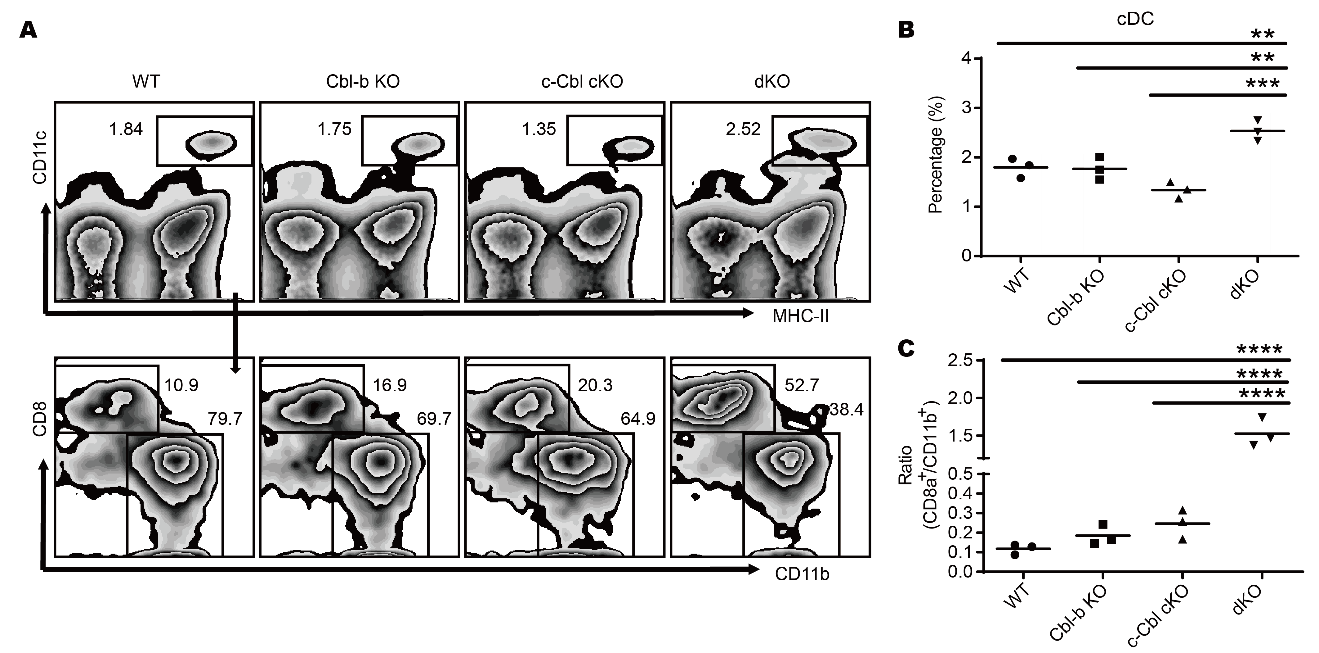
**

**Supplementary Figure. 2 cDC1s were accumulated in dKO spleen.** **(A)** Splenic cDCs and subsets were analyzed by flow cytometry. n=3 per group. **(B-C)** The percentage of cDCs **(B)** and the ratio of cDC1s/cDC2s **(C)** in spleen. Data are pooled from three independent experiments. ***p*<0.01, ****p*<0.001, *****p*<0.0001 based on One-Way ANOVA comparisons **(B and C)**. *p*<0.05 was considered statistically significant.


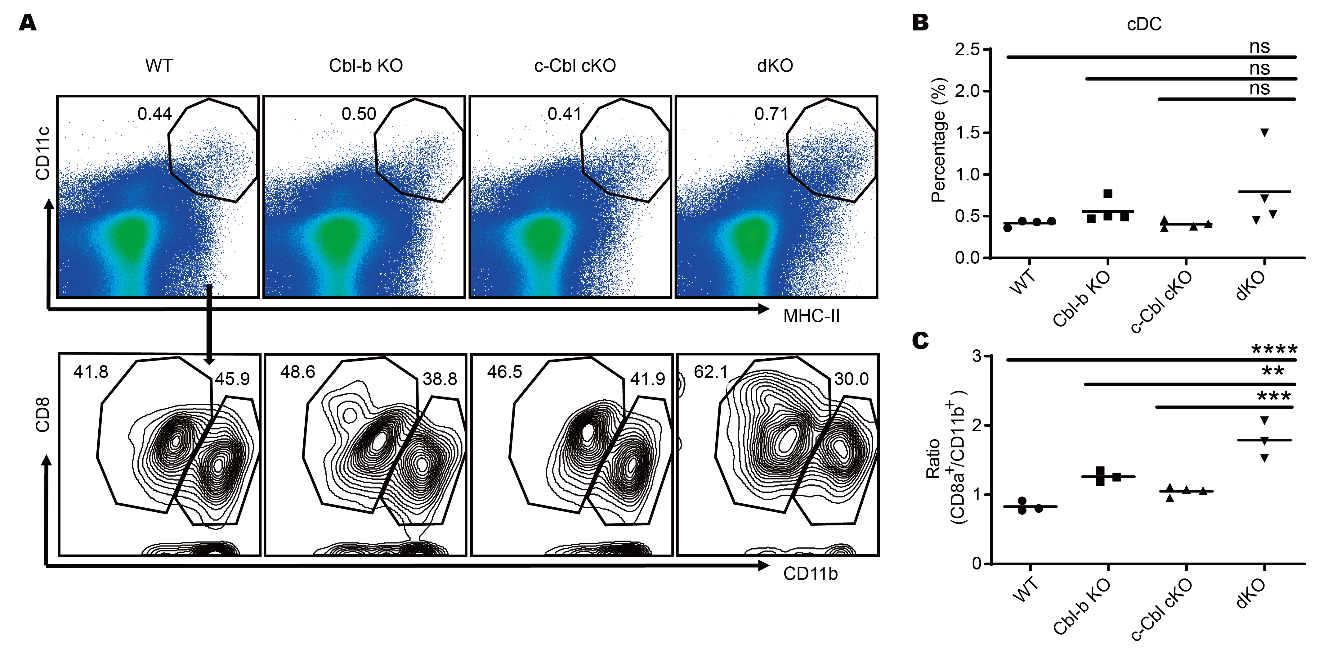


**Supplementary Figure. 3** **cDC1s were accumulated in dKO bone marrow. (A)** Flow cytometry analysis of bone marrow cDCs. n=4 per group. **(B-C)** The percentage of cDCs **(B)** and ratio of cDC1s/cDC2s **(C)** in bone marrow. ***p*<0.01, ****p*<0.001, *****p*<0.0001, ns, no significance based on One-Way ANOVA comparisons **(B and C)**. *p*<0.05 was considered statistically significant.

**
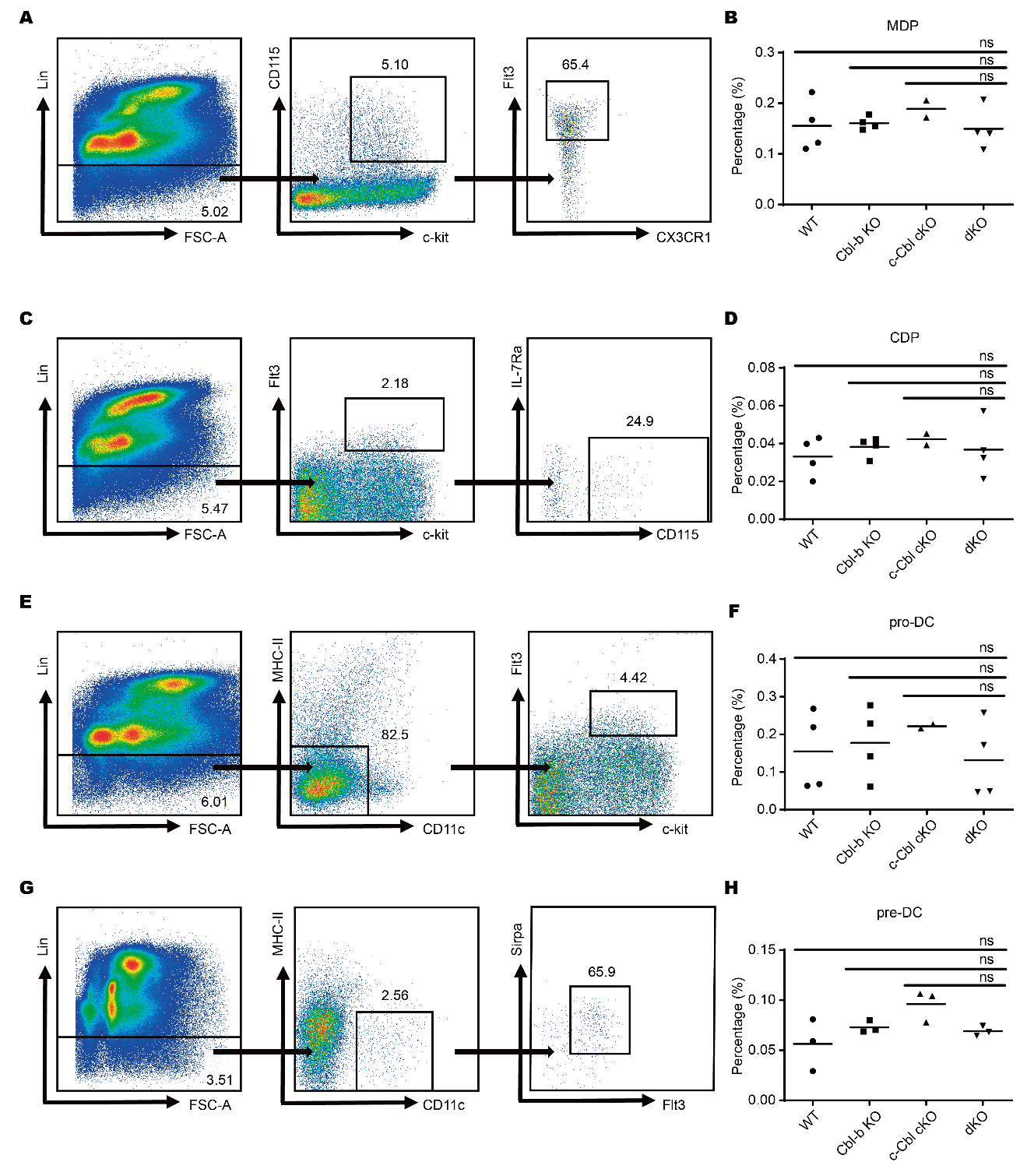
**

**Supplementary Figure. 4 Ablation of Cbl-b and c-Cbl in DCs did not influence the development of DCs. (A)** Flow cytometry analysis of bone marrow MDP (Lin^-^CD115^+^c-kit^+^Flt3^+^CX3CR1^-^) in bone marrow. **(B)** Percentage statistical analysis of bone marrow MDP. n=4 per group. **(C)** Flow cytometry analysis of the CDP (Lin^-^c-kit^+^Flt3^+^CD115^+^IL-7Ra^-^) in bone marrow. **(D)** Percentage statistical analysis of bone marrow CDP. n=4 per group. **(E)** Flow cytometry analysis of bone marrow pro-DC (Lin^-^MHC-II^-^CD11c^-^c-kit^+^Flt3^+^). **(F)** Percentage statistical analysis of bone marrow pro-DC. n=4 per group. **(G)** Flow cytometry analysis of bone marrow pre-DC (Lin^-^MHC-II^-^CD11c^+^Sirpa^+^Flt3^+^) in bone marrow. **(H)** Percentage statistical analysis of bone marrow pre-DC. n=3 per group. ns, no significance based on One-Way ANOVA comparisons **(B, D, F and H)**. *p*<0.05 was considered statistically significant.

**
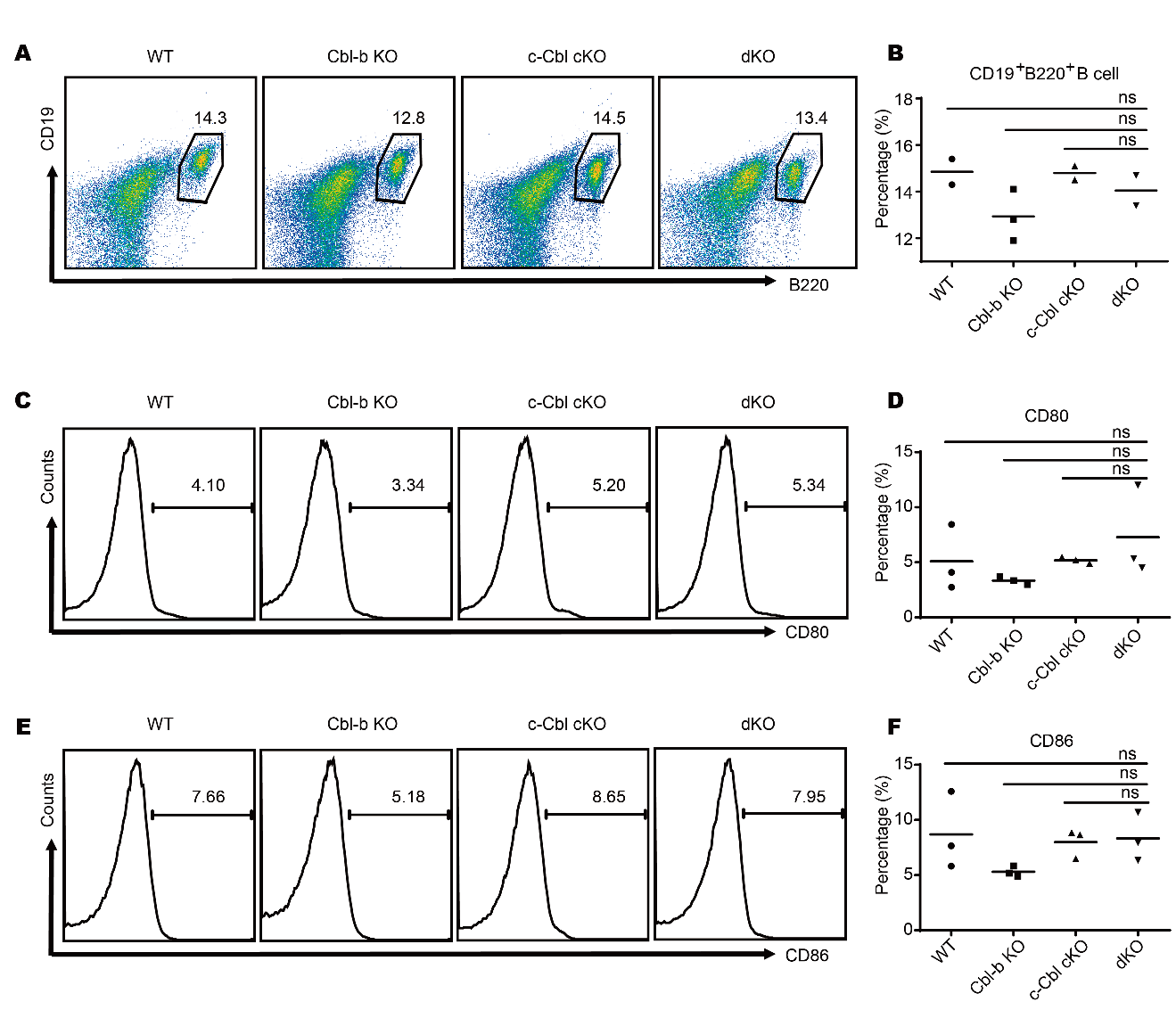
**

**Supplementary Figure. 5** **Ablation of Cbl-b and c-Cbl in DCs did not influence the development of B cells.** **(A-B)** The percentage of B cells in liver analyzed by Flow cytometry. n=3 per group. **(C, E)** The surface expression of CD80 **(C)** and CD86 **(E)** on B cells in liver detected by flow cytometry. n=3 per group. **(D, F)** The percentage of activated (CD80^+^/CD86^+^) B cells in liver detected in **(C, E)**. ns, no significance based on One-Way ANOVA comparisons **(B, D and F)**. *p*<0.05 was considered statistically significant.

**
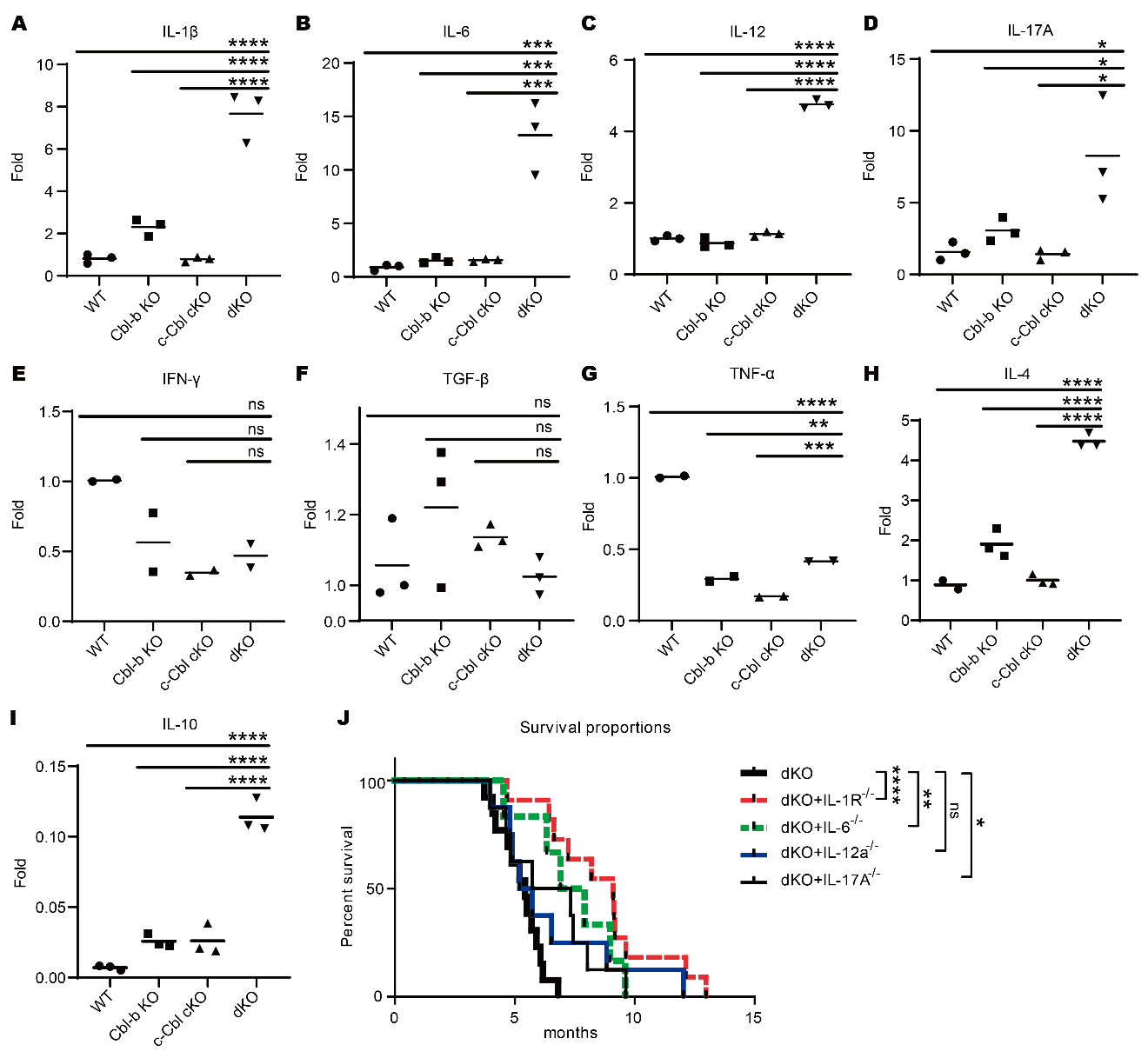
**

**Supplementary Figure. 6** **Proinflammation cytokines participated in ablation of Cbl-b/ c-Cbl-induced premature death. (A-G)** Real time PCR analysis of cytokines including IL-1β **(A)**, IL-6 **(B)**, IL-12 **(C)**, IL-17A **(D)**, IFN-γ **(E)**, TGF-β **(F)**, TNF-α **(G)**, IL-4 **(H)** and IL-10 **(I)** expression in bone marrow derived CD103^+^ cDCs. n=3 per group. **(J)** Survival curves of tKO (triple knockout) mice (n≧6 per group). **p*<0.05, ***p*<0.01, ****p*<0.001, *****p*<0.0001, ns, no significance based on One-Way ANOVA comparisons **(A-I)**. **p*<0.05, ***p*<0.01, *****p*<0.0001, ns, no significance based on log-rank test **(J)**. *p*<0.05 was considered statistically significant.

**Supplementary Table 1.** **Primers for qPCR.**

| **Gene** | **Direction** | **Sequence 5’- to - 3’** |
| --- | --- | --- |
| IL-1β | Forward | GAAGAAGTGCCCATCCTCTG |
|  | Reverse | AGCTCATATGGGTCCGACAG |
| IL-4 | Forward | GGTCTCAACCCCCAGCTAGT |
|  | Reverse | GCCGATGATCTCTCTCAAGTGAT |
| IL-6 | Forward | CCGGAGAGGAGACTTCACAG |
|  | Reverse | CAGAATTGCCATTGCACAAC |
| IL-10 | Forward | CAAGGCAGTGGAGCAGGTGAA |
|  | Reverse | CGGAGAGAGGTACAAACGAGGTT |
| IL-12 | Forward | AACCAGACCCGCCCAAGAAC |
|  | Reverse | GATCCTGAGCTTGCACGCAGA |
| IL-17A | Forward | GAGCTTCATCTGTGTCTCTG |
|  | Reverse | TCTTCATTGCGGTGGAGA |
| TNF-α | Forward | CCACACCGTCAGCCGATTTG |
|  | Reverse | CACCCATTCCCTTCACAGAGC |
| TGF-β | Forward | GGATACCAACTATTGCTTCAGTCC |
|  | Reverse | AGGCTCCAAATATAGGGGCAGGGTC |
| IFN-γ | Forward | GCCACGGCACAGTCATTGAAA |
|  | Reverse | TTTCGCCTTGCTGTTGCTGA |
| Bim | Forward | CTTCCATACGACAGTCTCAG |
|  | Reverse | CTCCATACCAGACGGAAGAT |
| Bcl-2 | Forward | TGTGGATGACTGAGTACCTG |
|  | Reverse | GGTTTGTCGACCTCACTTGT |
| Bcl-xL | Forward | GTAAACTGGGGTCGCATTGT |
|  | Reverse | TGGATCCAAGGCTCTAGGTG |
| β-actin | Forward | AACAGTCCGCCTAGAAGCAC |
|  | Reverse | CGTTGACATCCGTAAAGACC |

|  |
| --- |
|  |

**Supplementary Table 2. Primers for plasmid constructs.**

| **Gene** | **Direction** | **Sequence 5’- to - 3’** |
| --- | --- | --- |
| Cbl-b | Forward | CGGGGTACCGCCACCATGGCAAATTCTATGAATGGCA |
|  | Reverse | CCGCTCGAGCTACAGATCCTCTTCTGAGATGAGTTTTTGTTCTAGATTCAGACGTGGGGAGA |
| c-Cbl | Forward | CGGGGTACCGCCACCATGGCCGGCAACGTGAAGAA |
|  | Reverse | CCGCTCGAGCTACAGATCCTCTTCTGAGATGAGTTTTGTTCGGTGGCTACGTGAGCAGGA |
| STAT3 | Forward | CGGGGTACCGCCACCATGGCTCAGTGGAACCAGCT |
|  | Reverse | CCGCTCGAGTCACTTGTCATCGTCGTCCTTGTAATCCATGGGGGAGGTAGCACACT |
| STAT5a | Forward | CCGGAATTCGCCACCATGGCGGGCTGGATTCAGGC |
|  | Reverse | CGGGGTACCTCACTTGTCATCGTCGTCCTTGTAATCGGACAGGGAGCTTCTAGCGG |
| STAT5b | Forward | CCGGAATTCGCCACCATGGCTATGTGGATACAGGC |
|  | Reverse | CGGGGTACCTCACTTGTCATCGTCGTCCTTGTAATCTGACTGTGCGTGAGGGATC |
| GAS1 | Forward | GAAGAGGGGTGAGCATCTTG |
|  | Reverse | CAGTTGGAAGCCTCAGAAGG |
| GAS2 | Forward | GGGTCGGTACTGGCATCTAA |
|  | Reverse | GCTCGGCGTTAATCACTTTC |

**Supplementary Table 3. Primers for mutant STAT5 constructs.**

| **Gene** | **Direction** | **Sequence 5’- to - 3’** |
| --- | --- | --- |
| STAT5a K71R | Forward | TGGTGCTCCGCCTGCTTCTGCAGCTCC |
|  | Reverse | GGAGCTGCAGAAGCAGGCGGAGCACCA |
| STAT5a K163R | Forward | GTCTGCTGCAGCTTCCTCAGCTCGTTCTCCG |
|  | Reverse | CGGAGAACGAGCTGAGGAAGCTGCAGCAGAC |
| STAT5a K164R | Forward | CTGCTGCAGCCTCTTCAGCTCGTTCTCCGT |
|  | Reverse | ACGGAGAACGAGCTGAAGAGGCTGCAGCAG |
| STAT5a K207R | Forward | GGGACACTTGCCTCTGCTGGAGGGCC |
|  | Reverse | GGCCCTCCAGCAGAGGCAAGTGTCCC |
| STAT5a K235R | Forward | GCAGCTGCAGGGTCCTCTGGTGCTTCTCA |
|  | Reverse | TGAGAAGCACCAGAGGACCCTGCAGCTGC |
| STAT5a K242R | Forward | ATGGTCTGCTGCCTCCGCAGCAGCTGC |
|  | Reverse | GCAGCTGCTGCGGAGGCAGCAGACCAT |
| STAT5a K384R | Forward | CGGGTGTTCTCATTCCTGAGCAGGGACTTGG |
|  | Reverse | CCAAGTCCCTGCTCAGGAATGAGAACACCCG |
| STAT5a K516R | Forward | CTGTACTTCAGCCCTGAATTTCATGTTGAGCGCTTCACA |
|  | Reverse | TGTGAAGCGCTCAACATGAAATTCAGGGCTGAAGTACAG |
| STAT5a K527R | Forward | CACGAGGTTCTCTCTGGTCAAGCCCCGGT |
|  | Reverse | ACCGGGGCTTGACCAGAGAGAACCTCGTG |
| STAT5a K644R | Forward | GTCGTGAATGGCCTCAGATTCCAGAGGTTTCGGTCC |
|  | Reverse | GGACCGAAACCTCTGGAATCTGAGGCCATTCACGAC |
| STAT5a K675R | Forward | GACCTCGTCCCTGGGTCGGTCTGGG |
|  | Reverse | CCCAGACCGACCCAGGGACGAGGTC |
| STAT5a K689R | Forward | CCGTCAACTGCTCTCGCAAGTACAGGAGTGTAATA |
|  | Reverse | TATTACACTCCTGTACTTGCGAGAGCAGTTGACGG |
| STAT5a K696R | Forward | TTGATCTGTGGCCTCACGTATCCGTCAACTGCTTT |
|  | Reverse | AAAGCAGTTGACGGATACGTGAGGCCACAGATCAA |
| STAT5b K140R | Forward | CTGAAGGTGCCTCTGGGACATGGCGTCAGC |
|  | Reverse | GCTGACGCCATGTCCCAGAGGCACCTTCAG |
| STAT5b K694R | Forward | GTCAGCTGCTCTCGCAGTTGCGGGCTCACA |
|  | Reverse | TGTGAGCCCGCAACTGCGAGAGCAGCTGAC |
