# Supplementary material for: Ablation of Cbl-b and c-Cbl in dendritic cells causes spontaneous liver cirrhosis via altering multiple properties of CD103+ cDC1s
Source: Cell Death Discov. 2022 Mar 30;8:142. doi: 10.1038/s41420-022-00953-2 (PMC8967913; doi:10.1038/s41420-022-00953-2)
Supplement: Supplementary file 2 — Original Data File [file 41420_2022_953_MOESM2_ESM.docx]

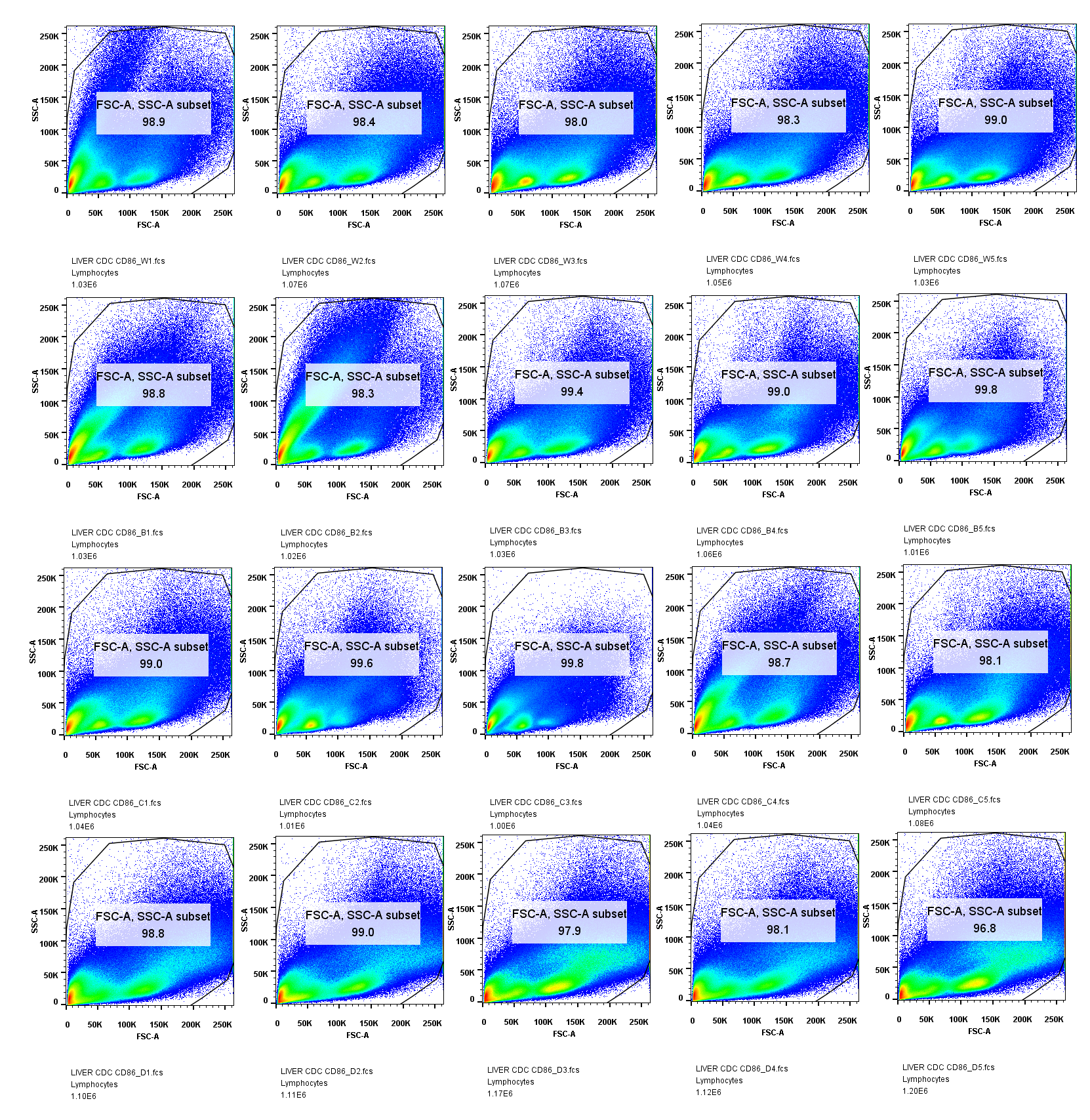
**Figure 2B**


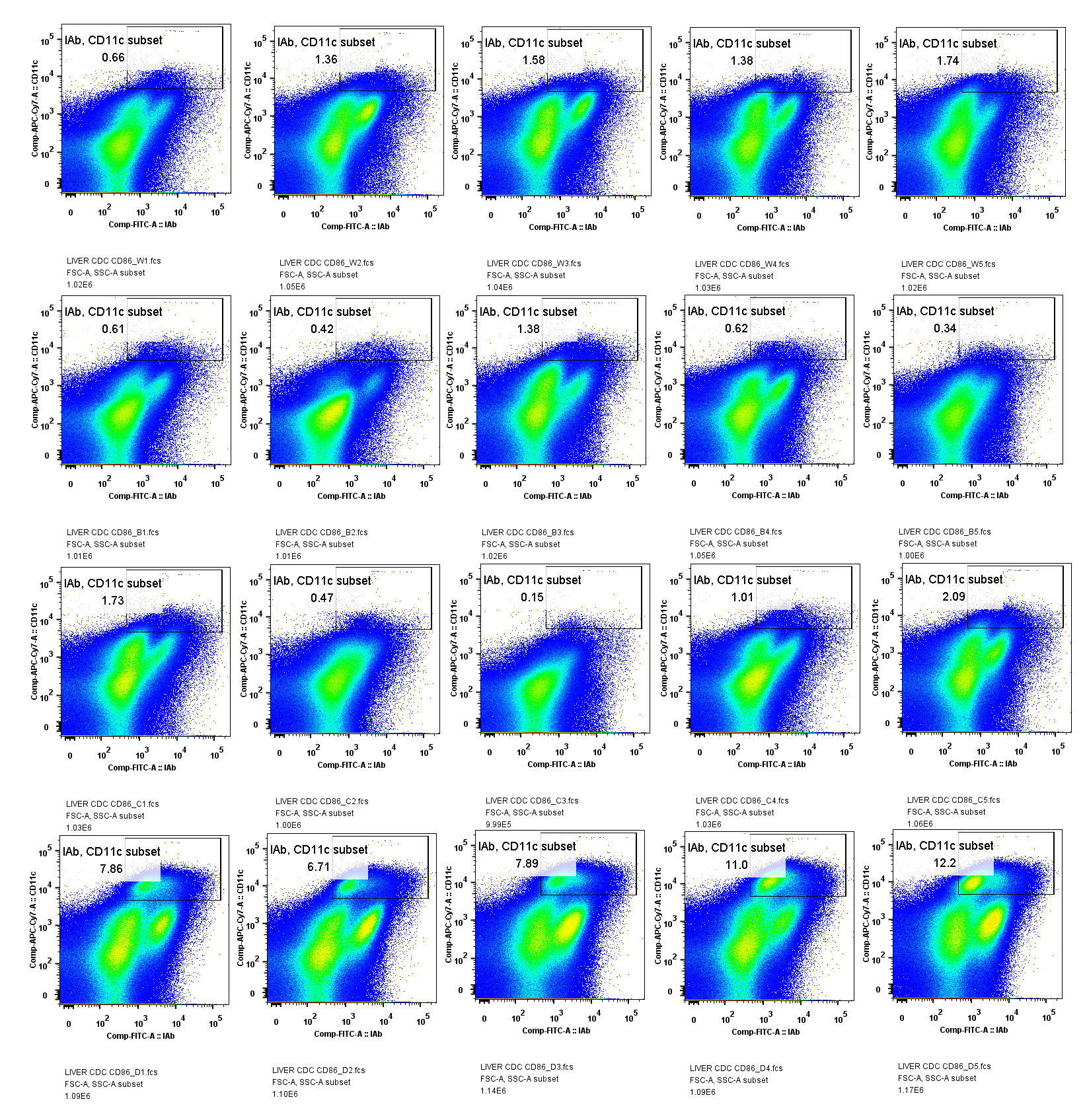
**Figure 2B**


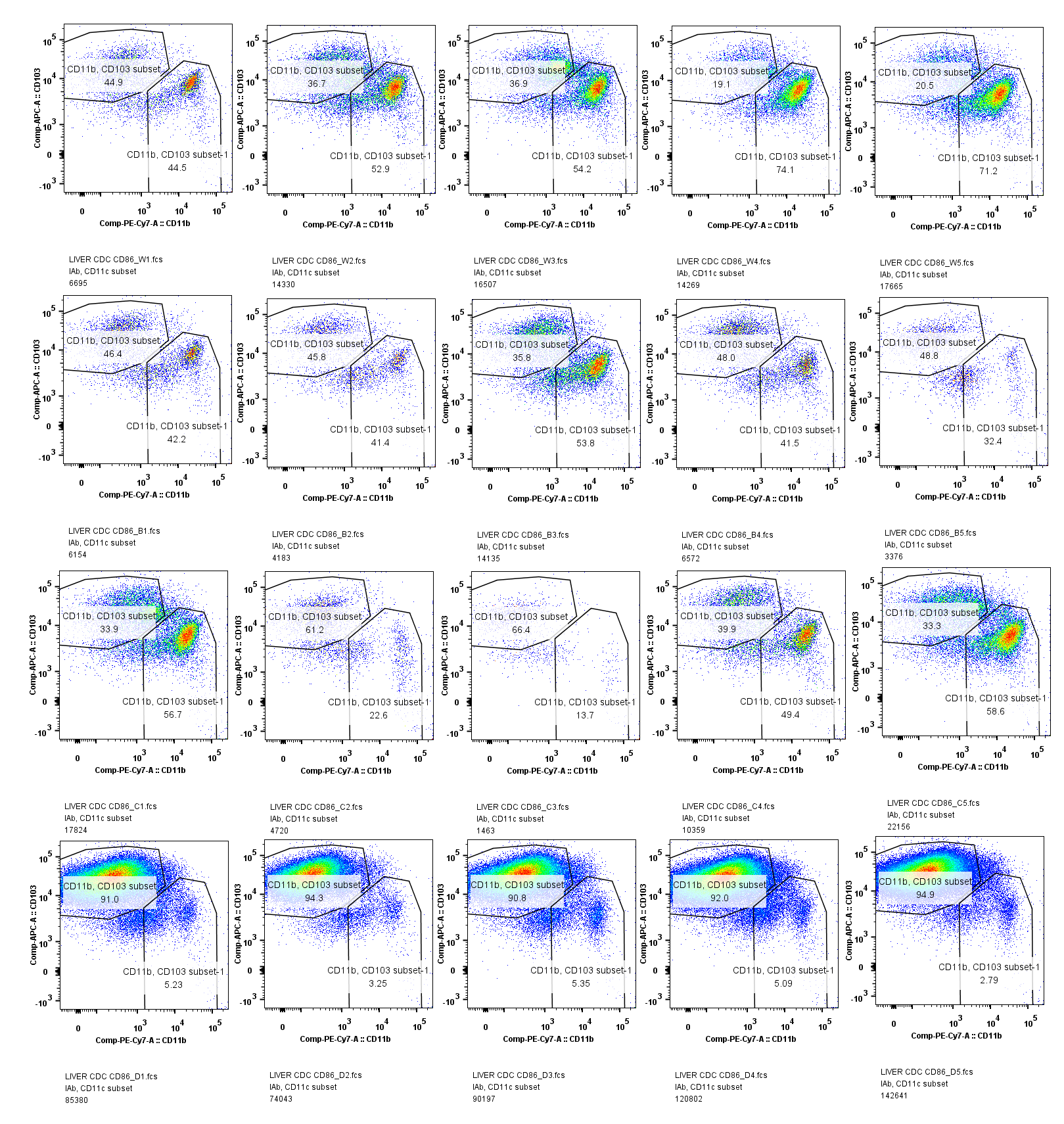
**Figure 2B**


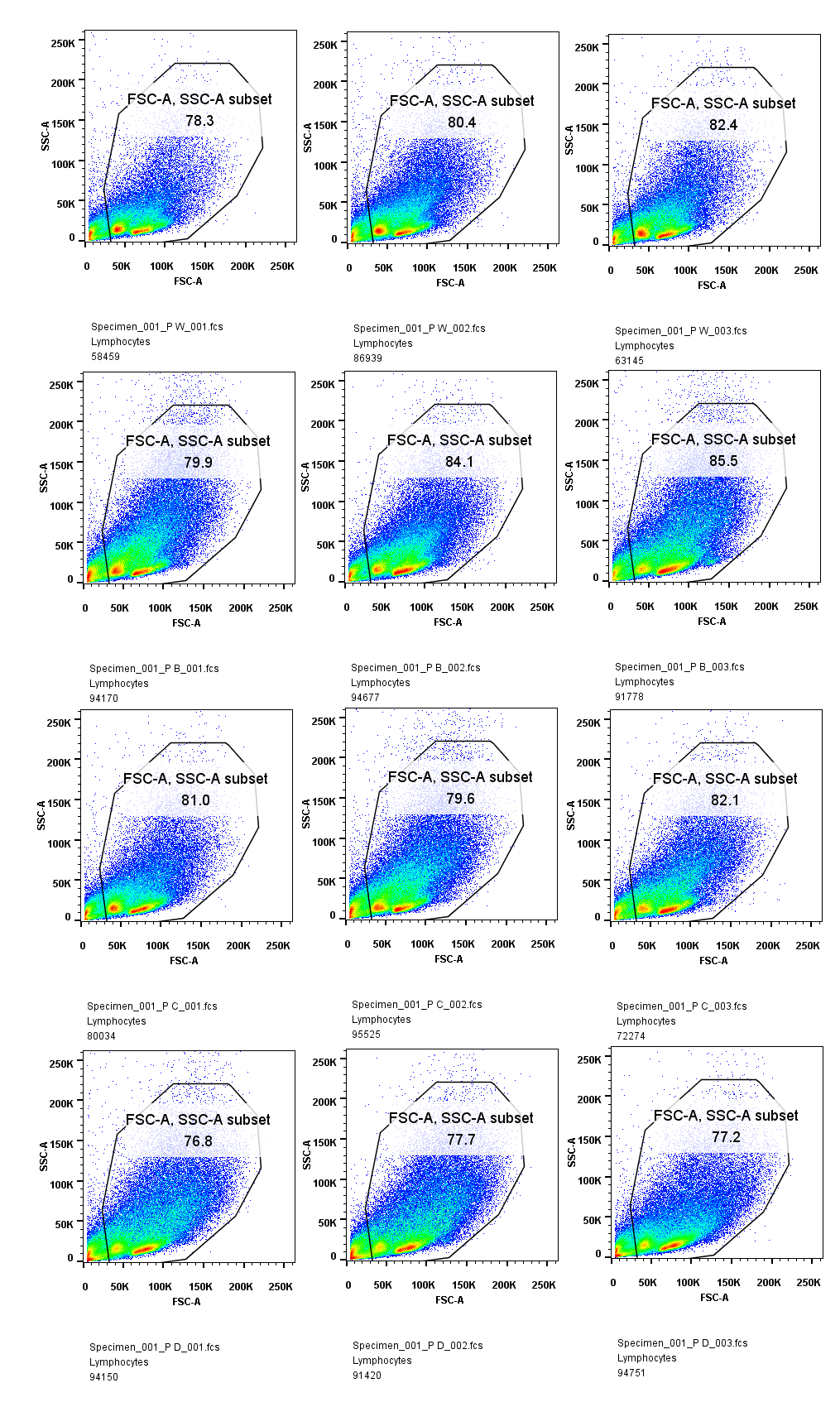
**Figure 2H**


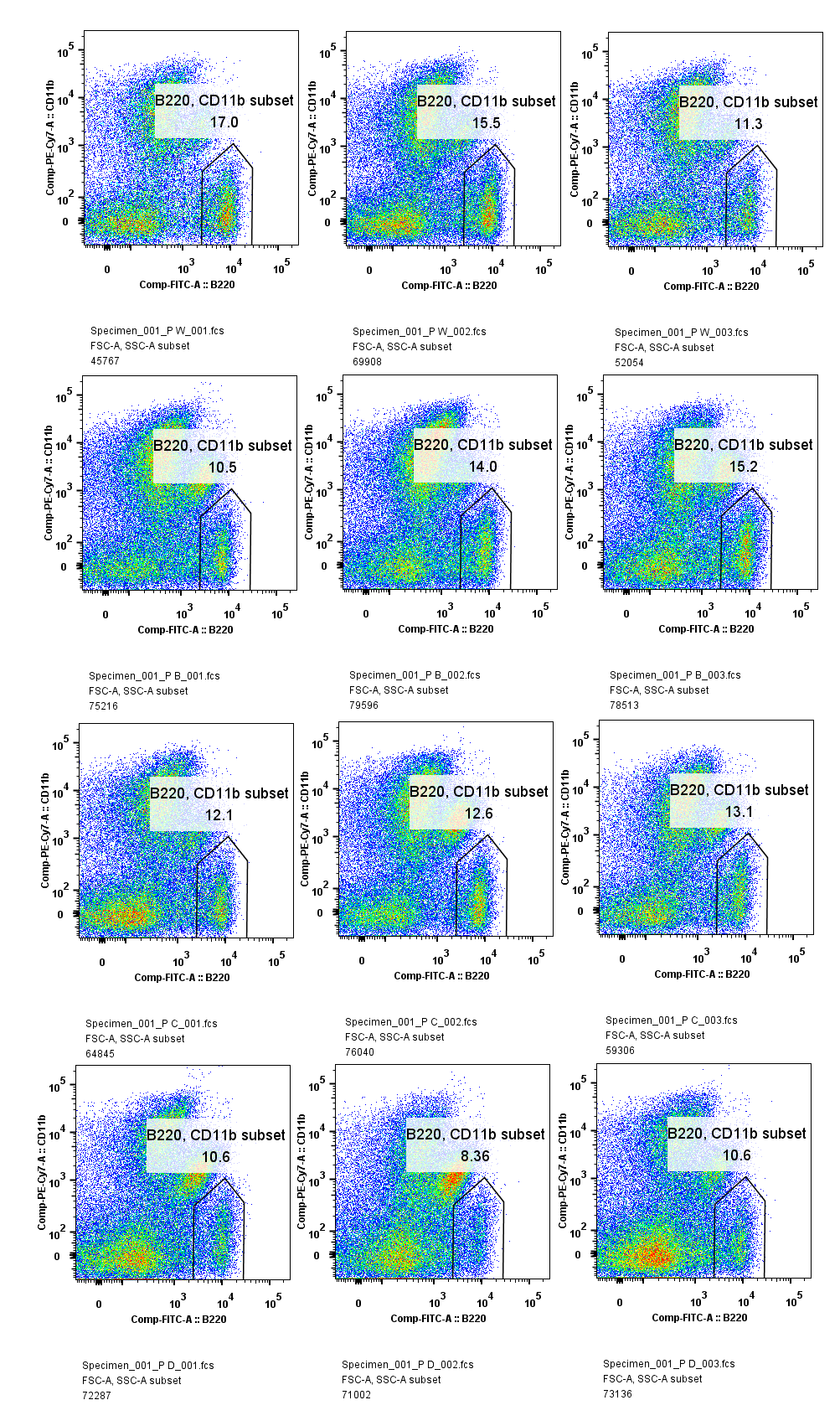
**Figure 2H**

**Figure 2**
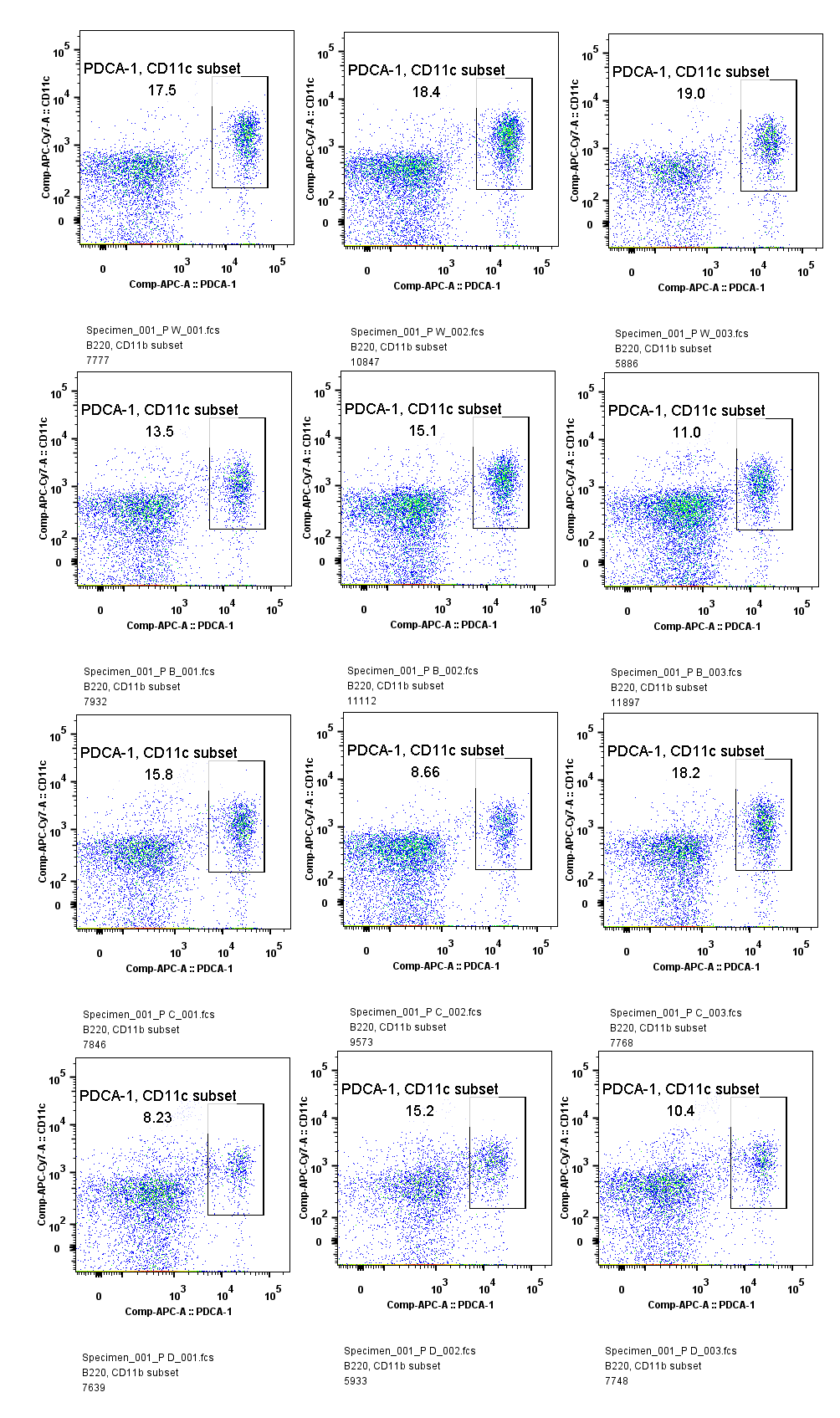
**H**


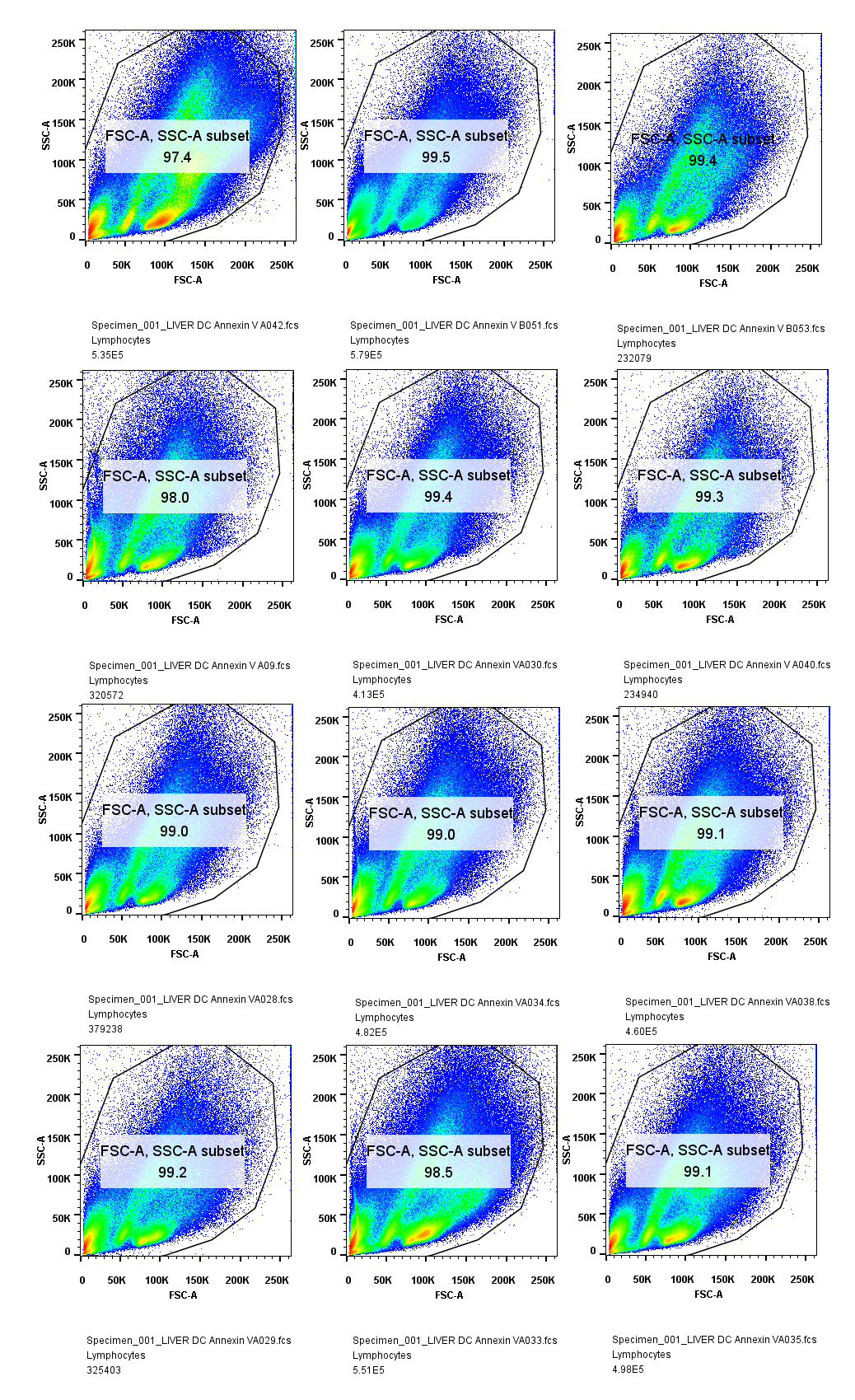
**Figure 3A**


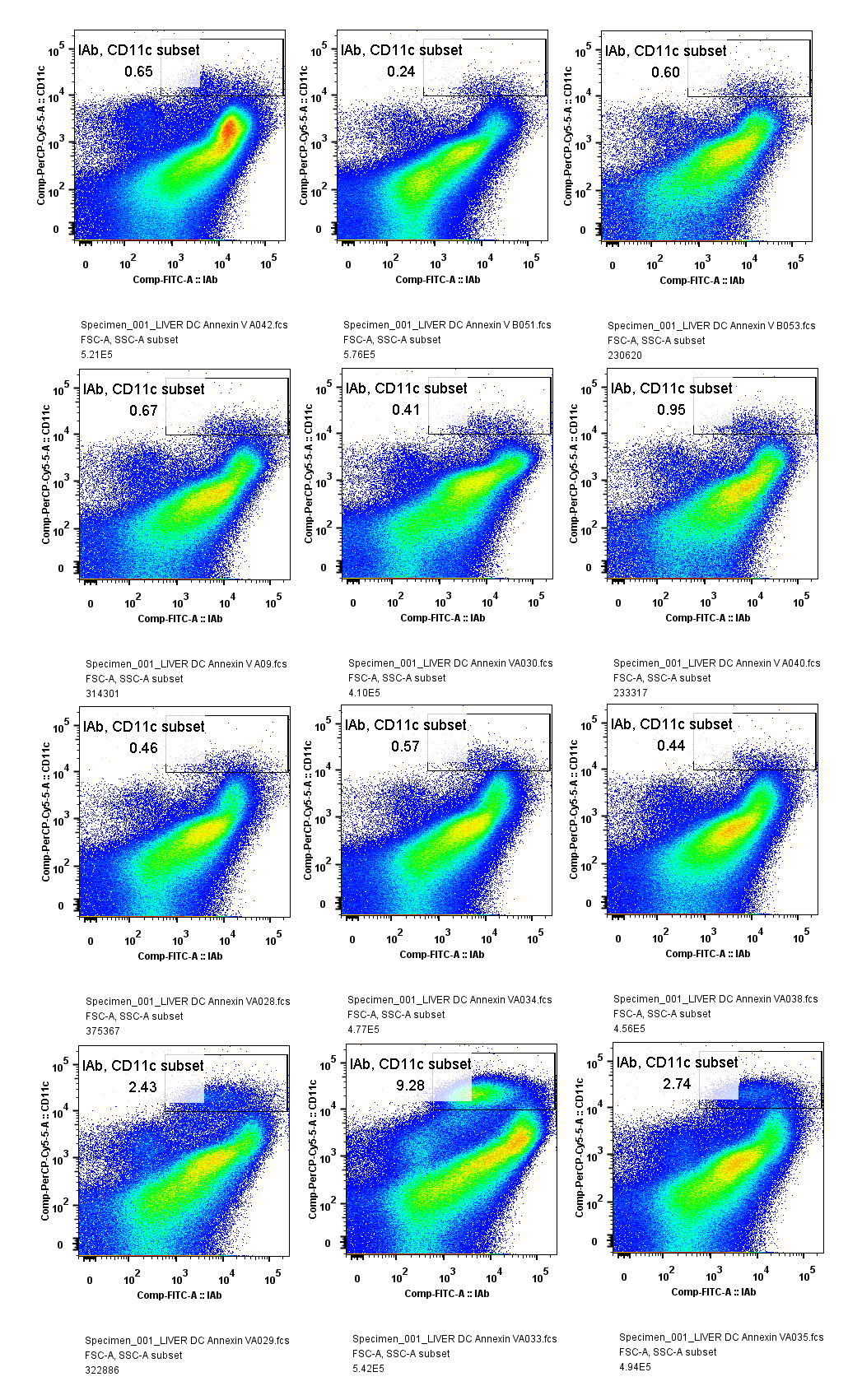
**Figure 3A**


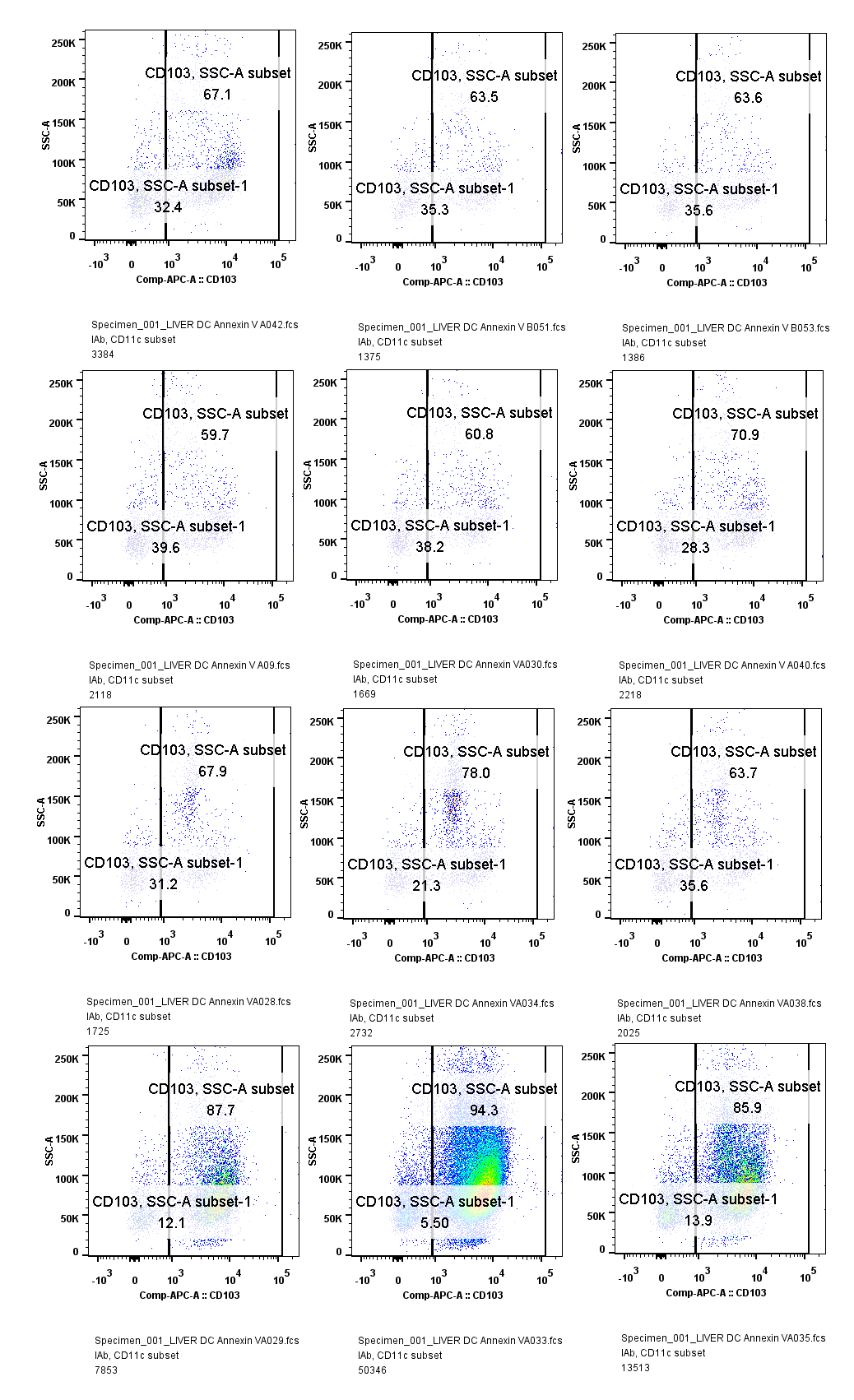
**Figure 3A**


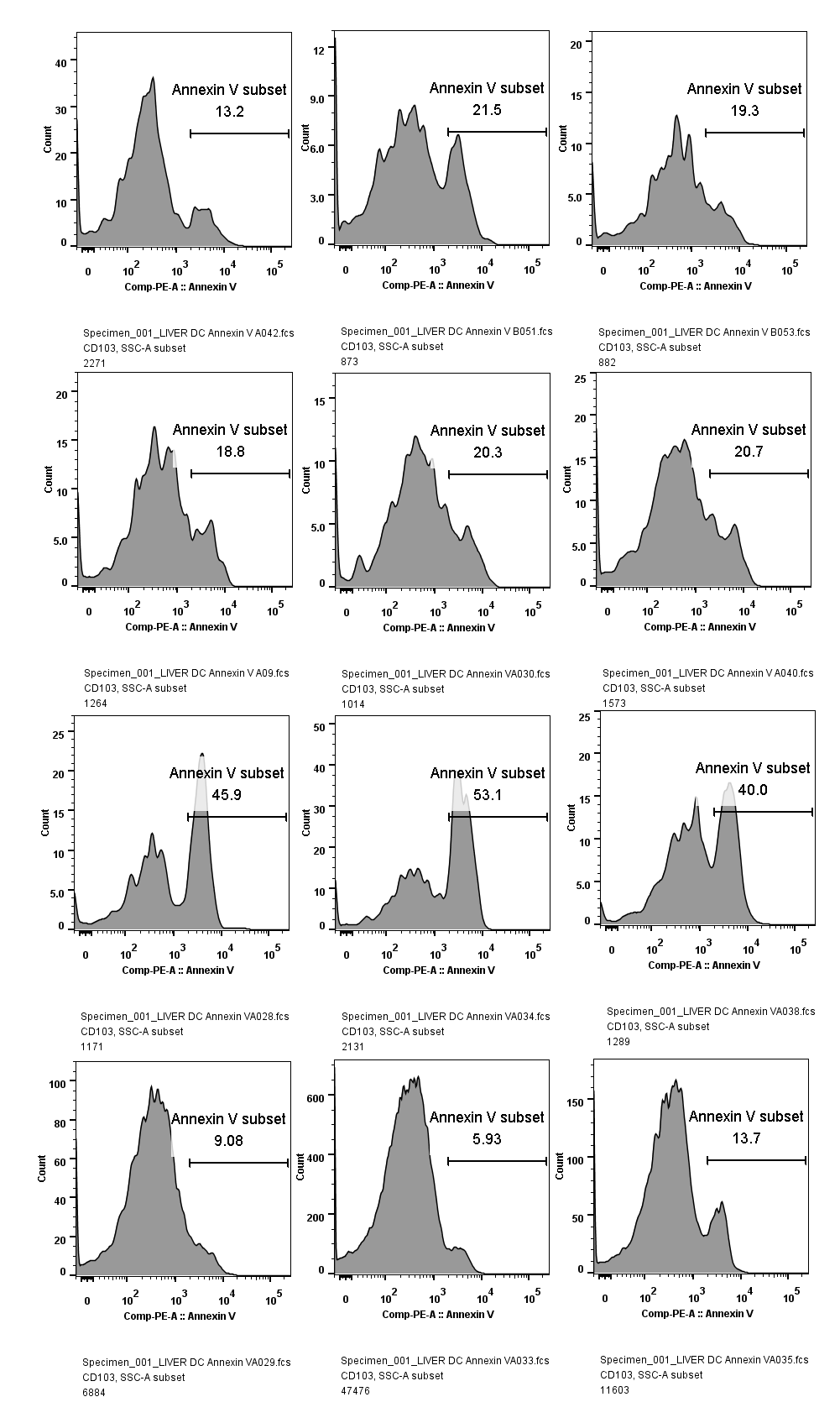
**Figure 3A**

**Figure 3A**

**WT A042 B051 B052**

**Cbl-b KO A09 A030 A040**

**c-Cbl cKO A028 A034 A038**

**dKO A029 A033 A035**

**Figure 3C**


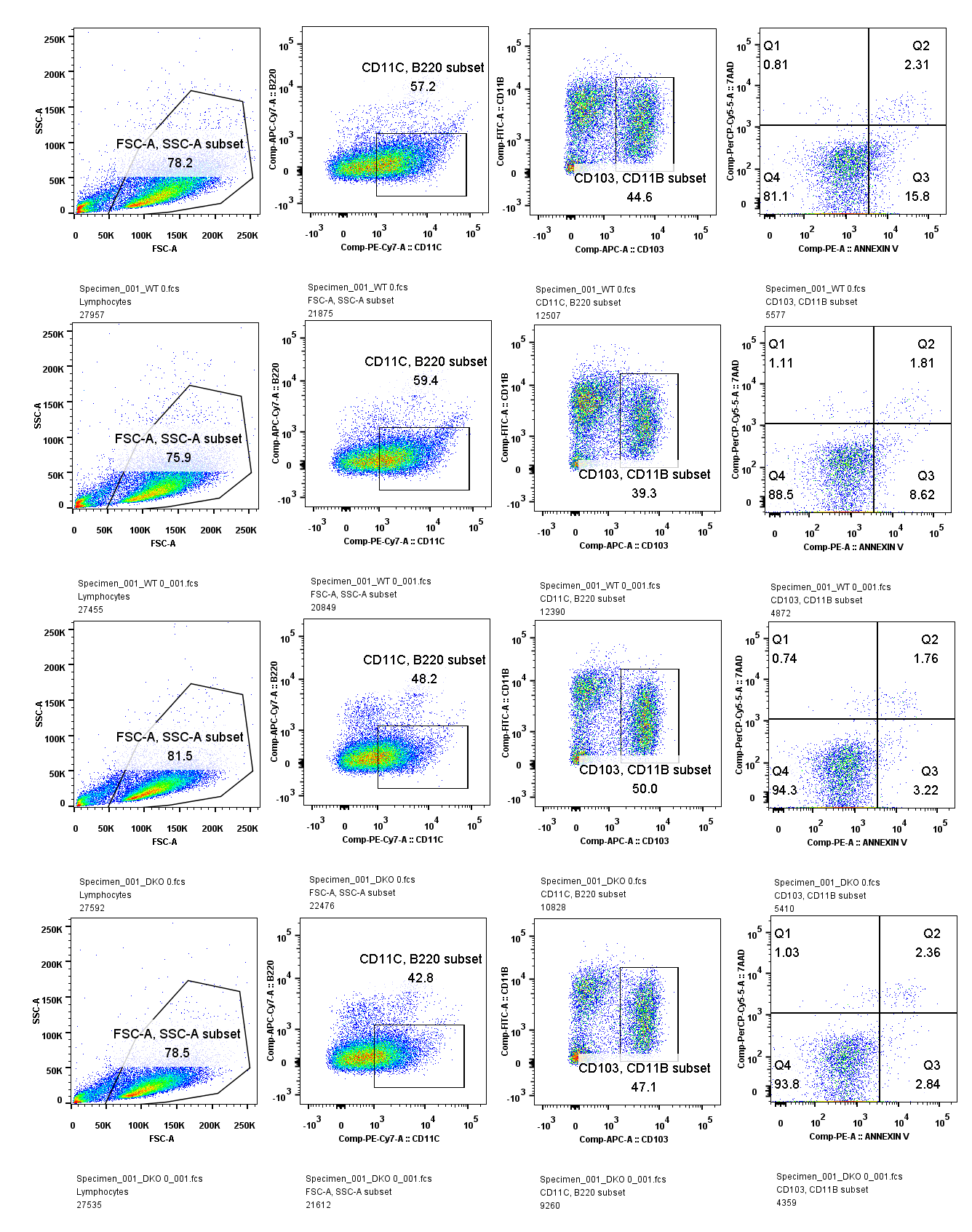


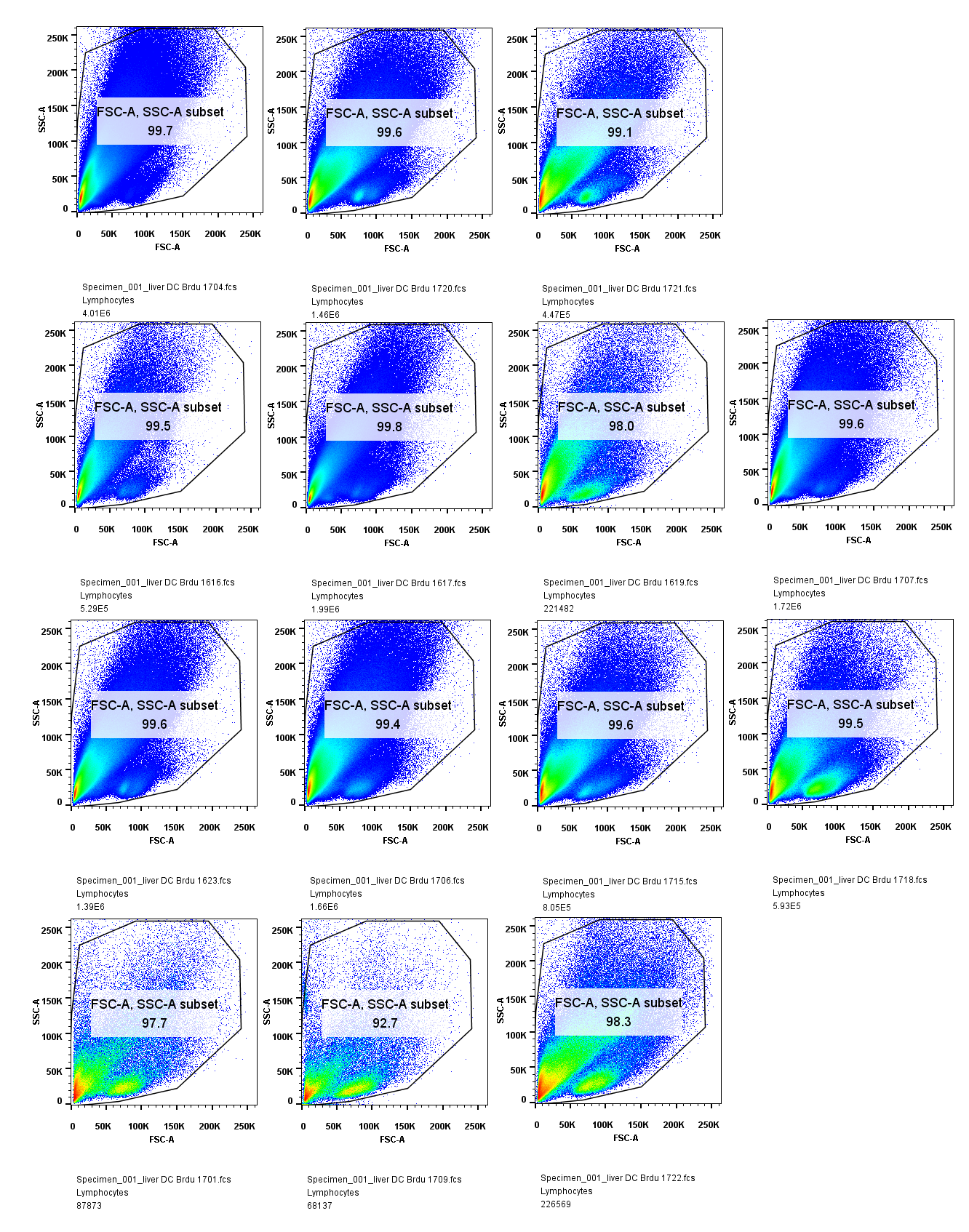
**Figure 3F**


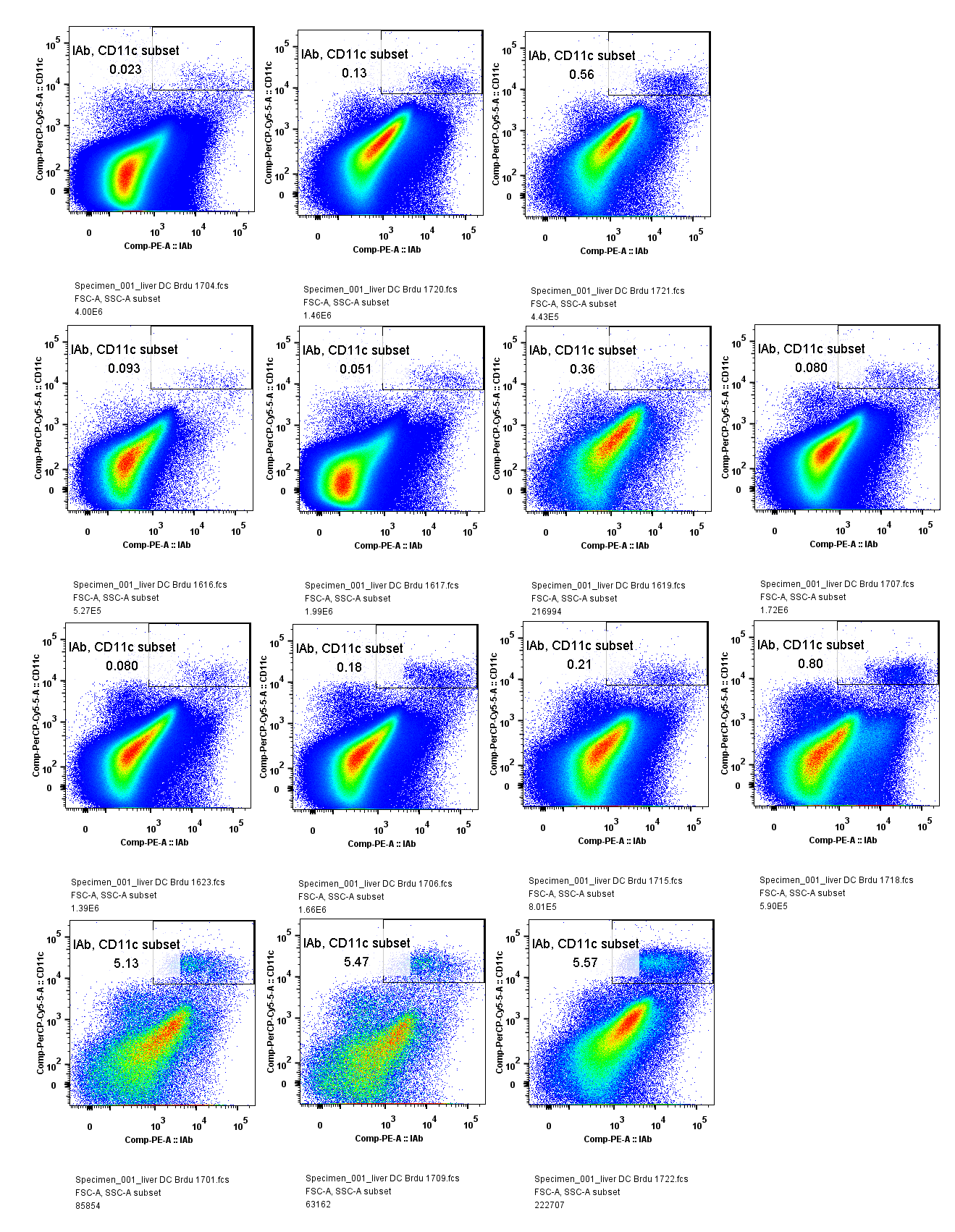
**Figure 3F**


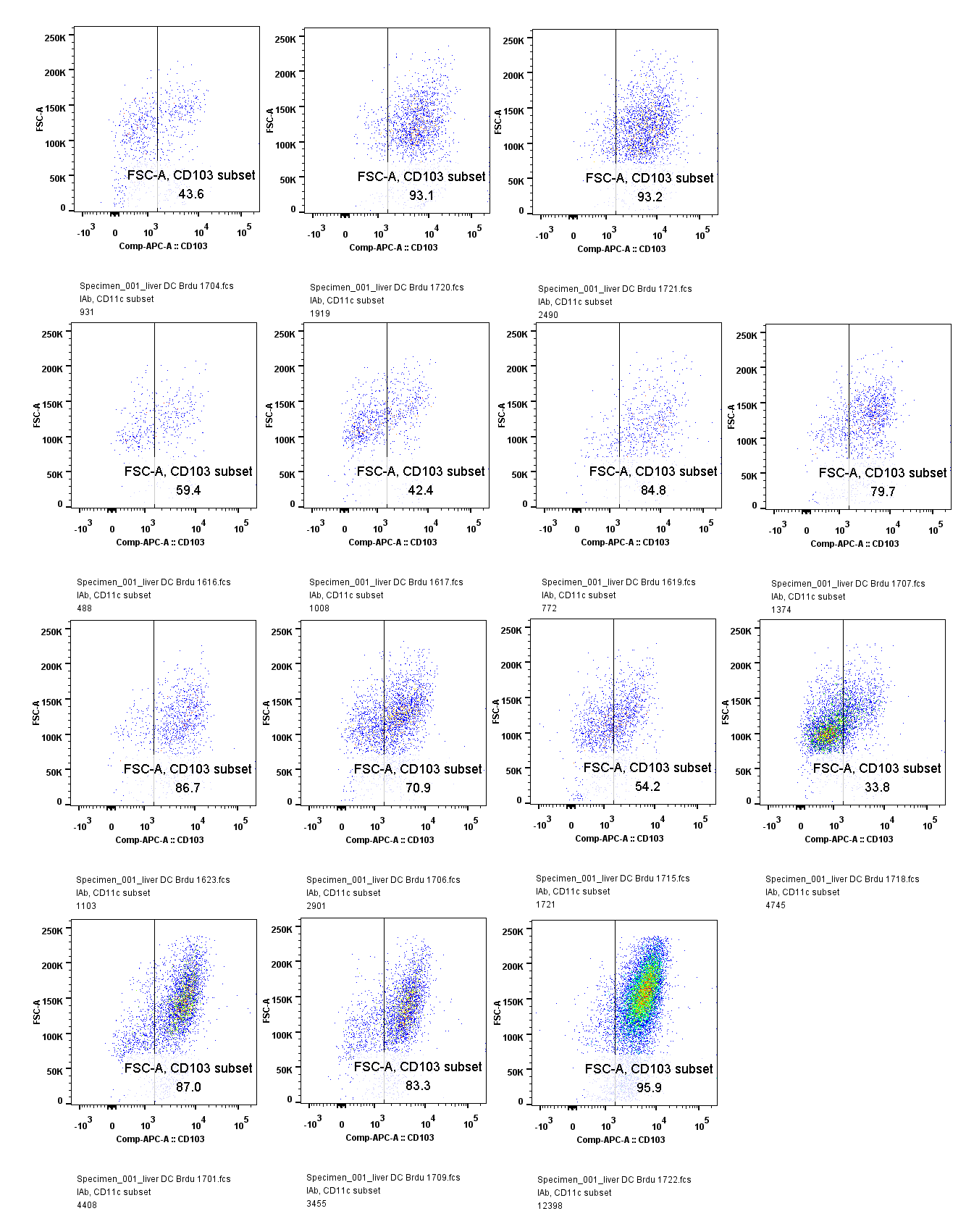
**Figure 3F**

**
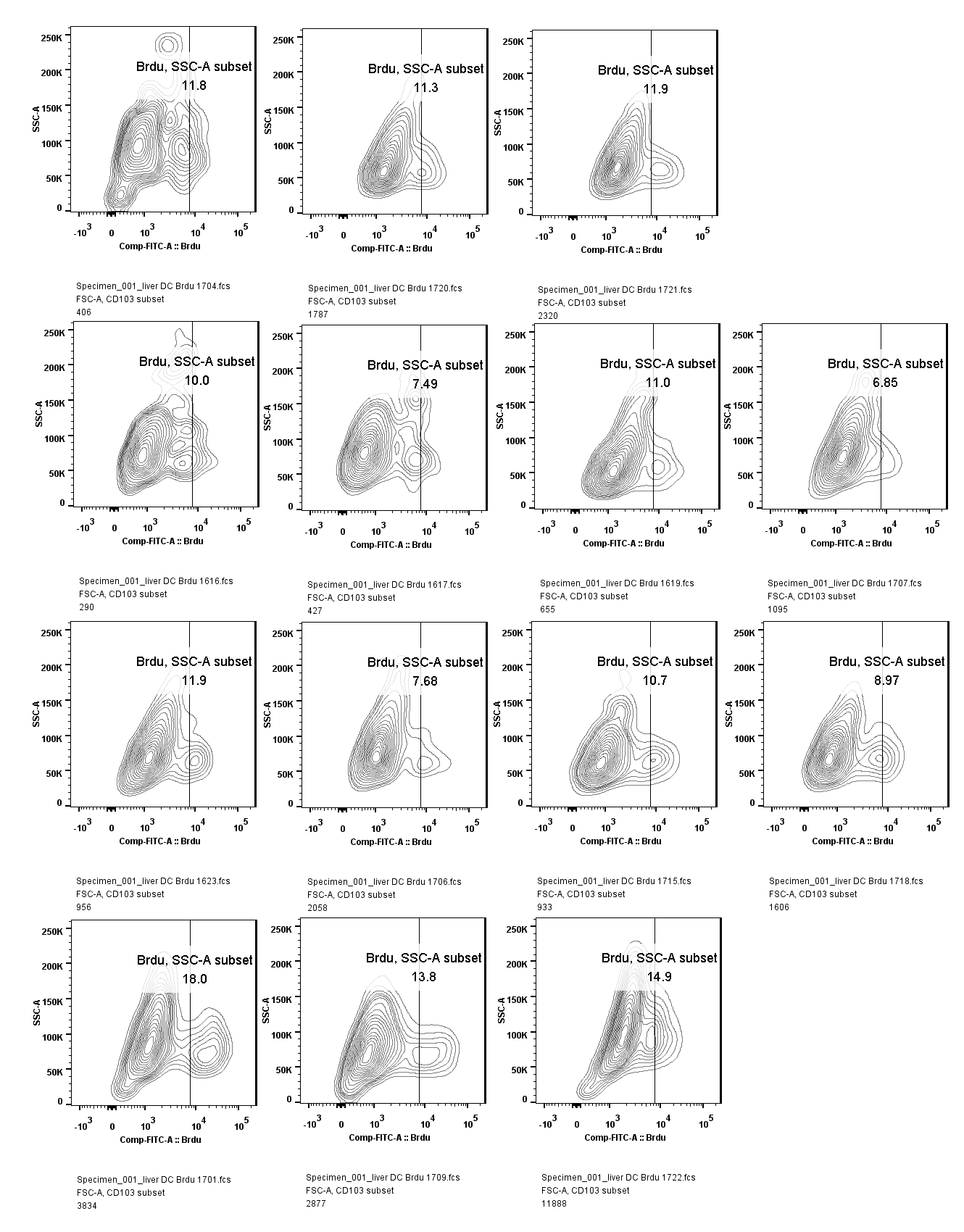
Figure 3F**

**WT 1704 1720 1721**

**Cbl-b KO 1623 1706 1715 1718**

**c-Cbl cKO 1616 1617 1619 1707**

**dKO 1701 1709 1722**


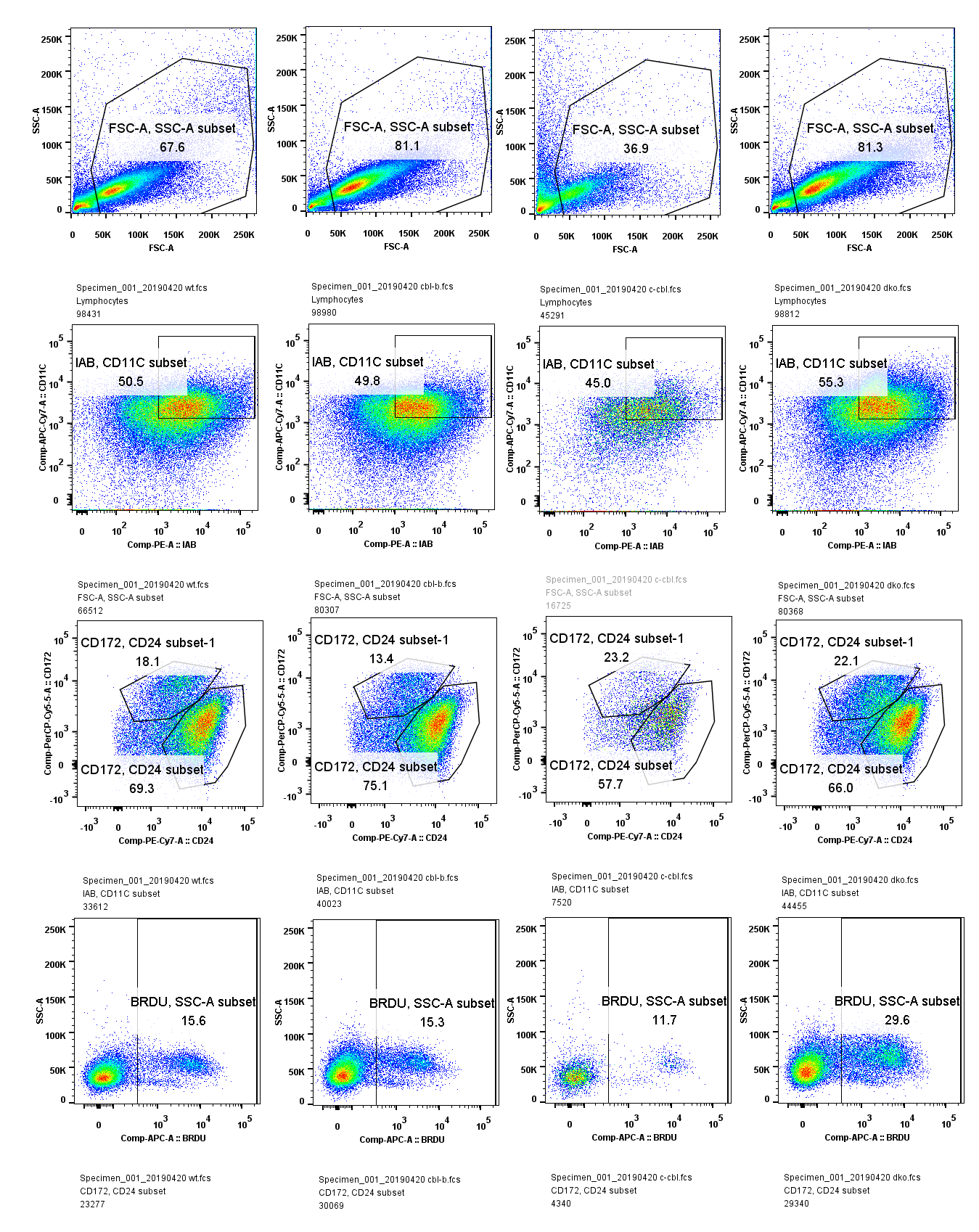
**Figure 3H**


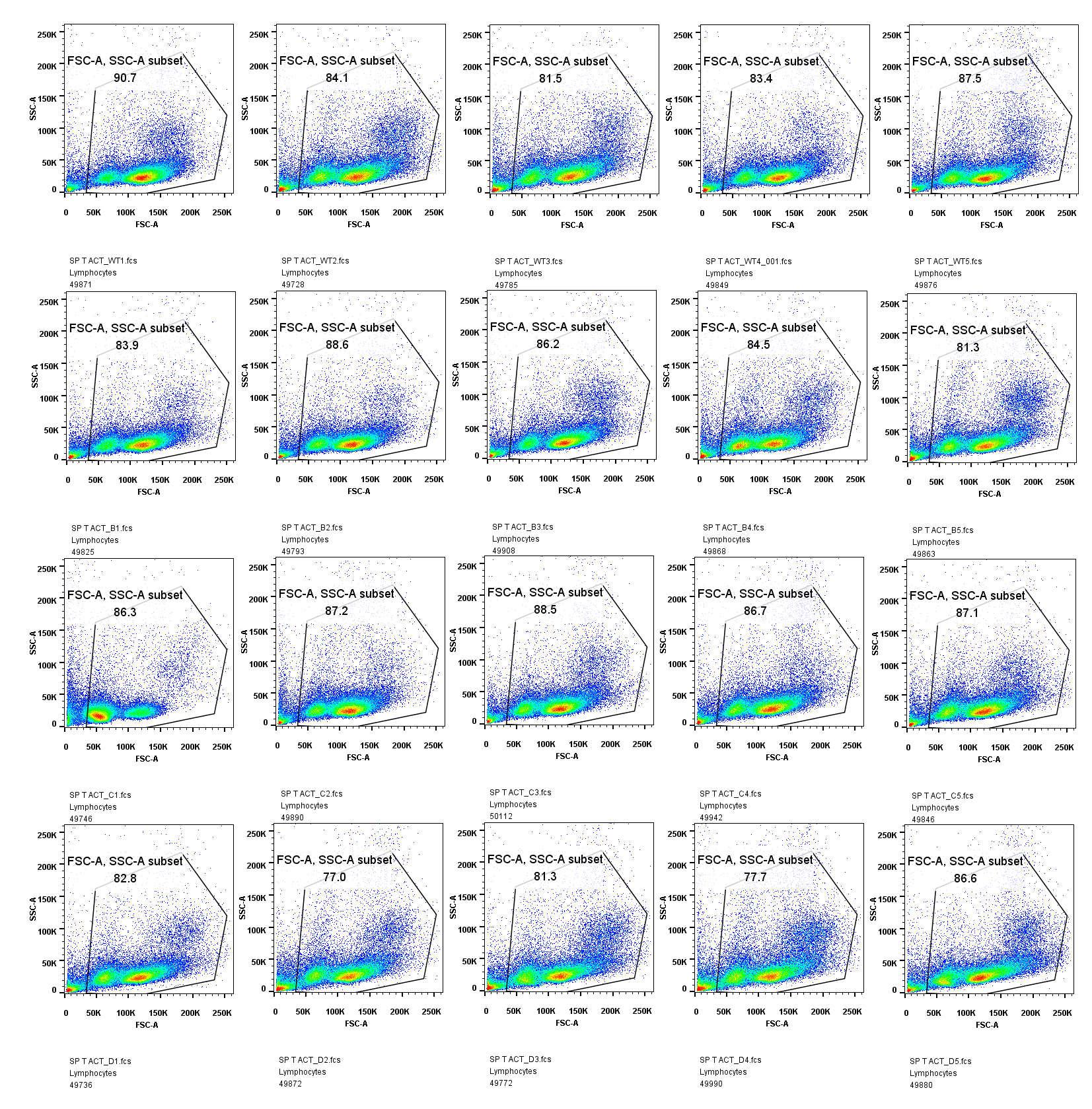
**Figure 4A**


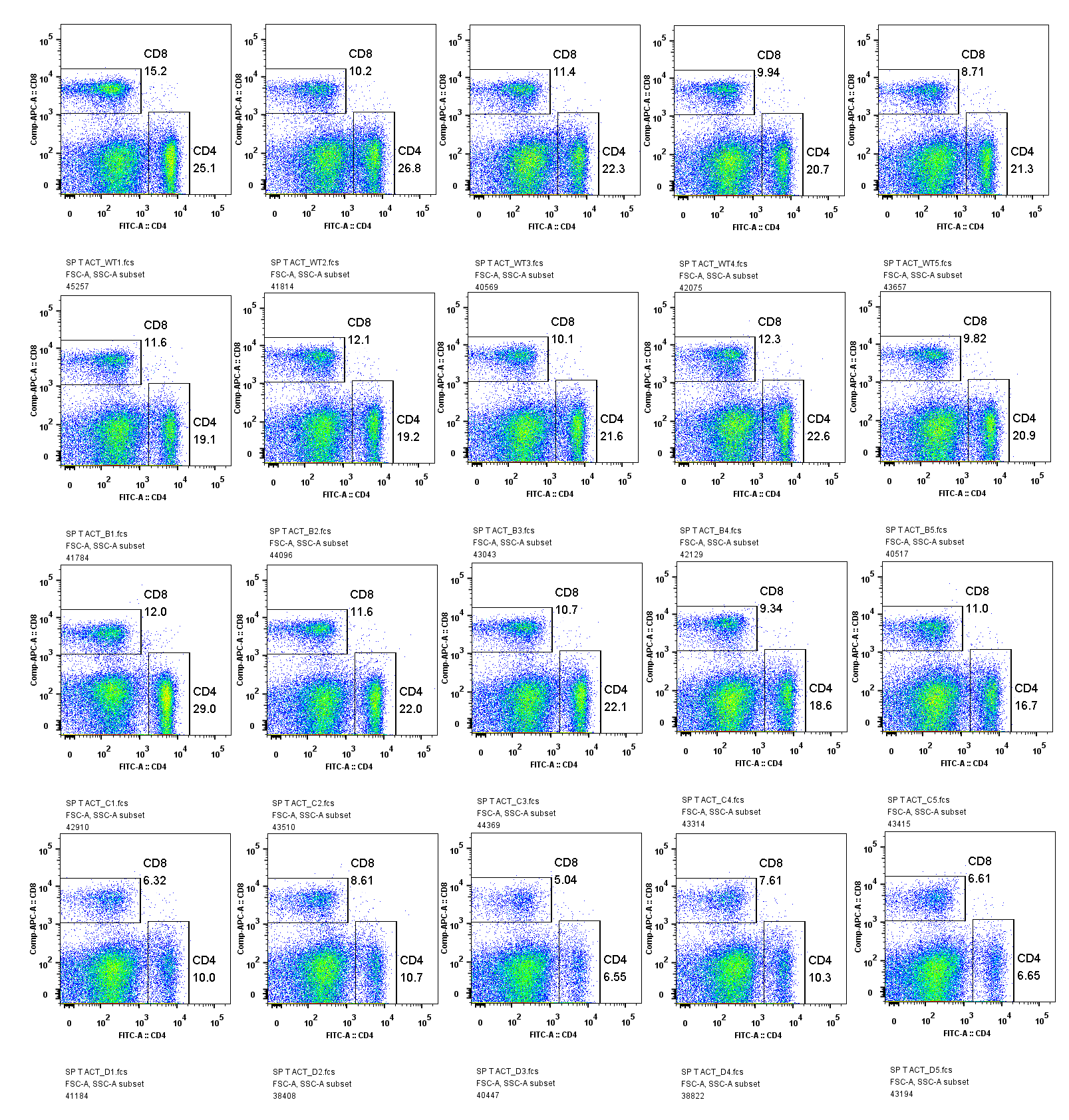
**Figure 4A**

**Figure 4A**


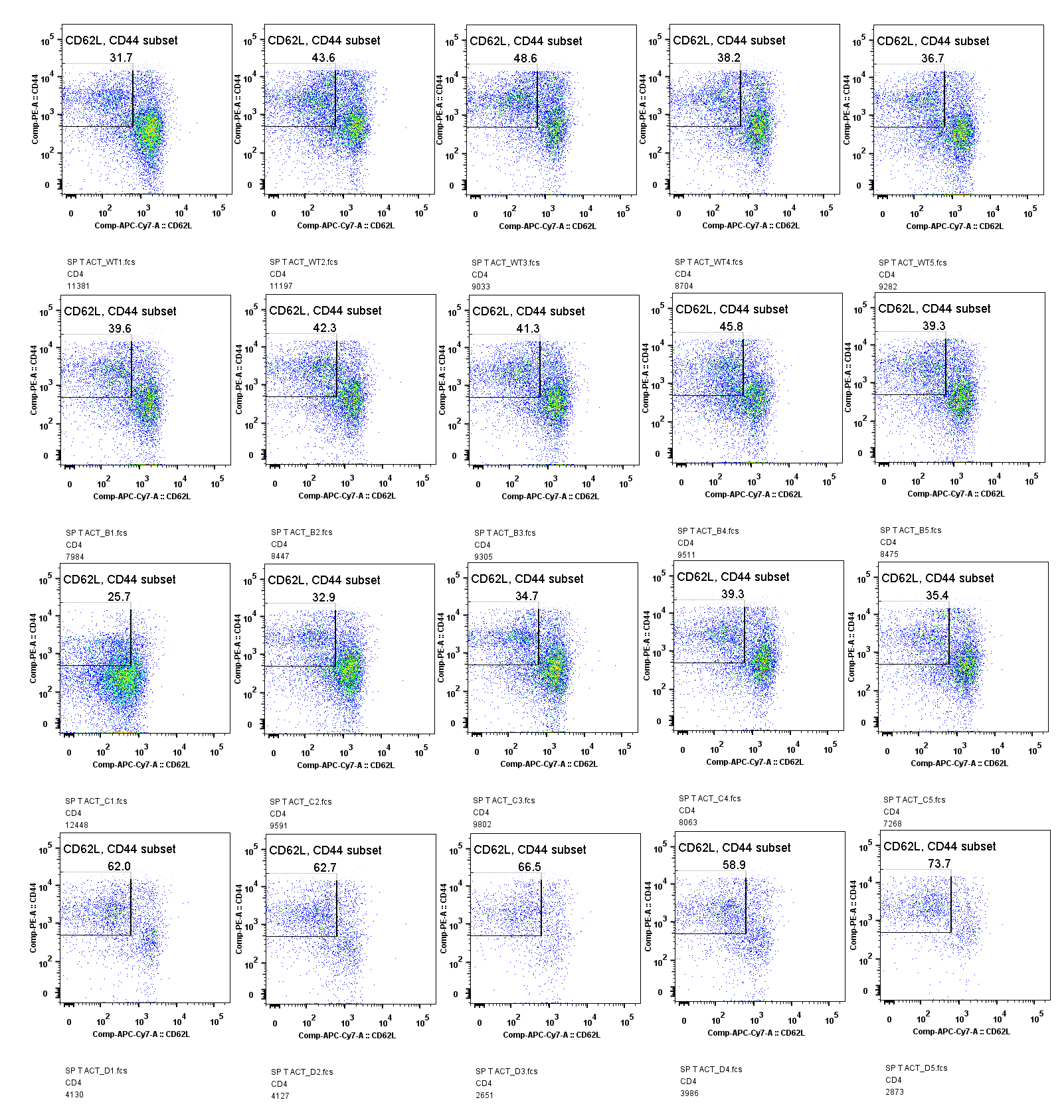


**Figure 4A**


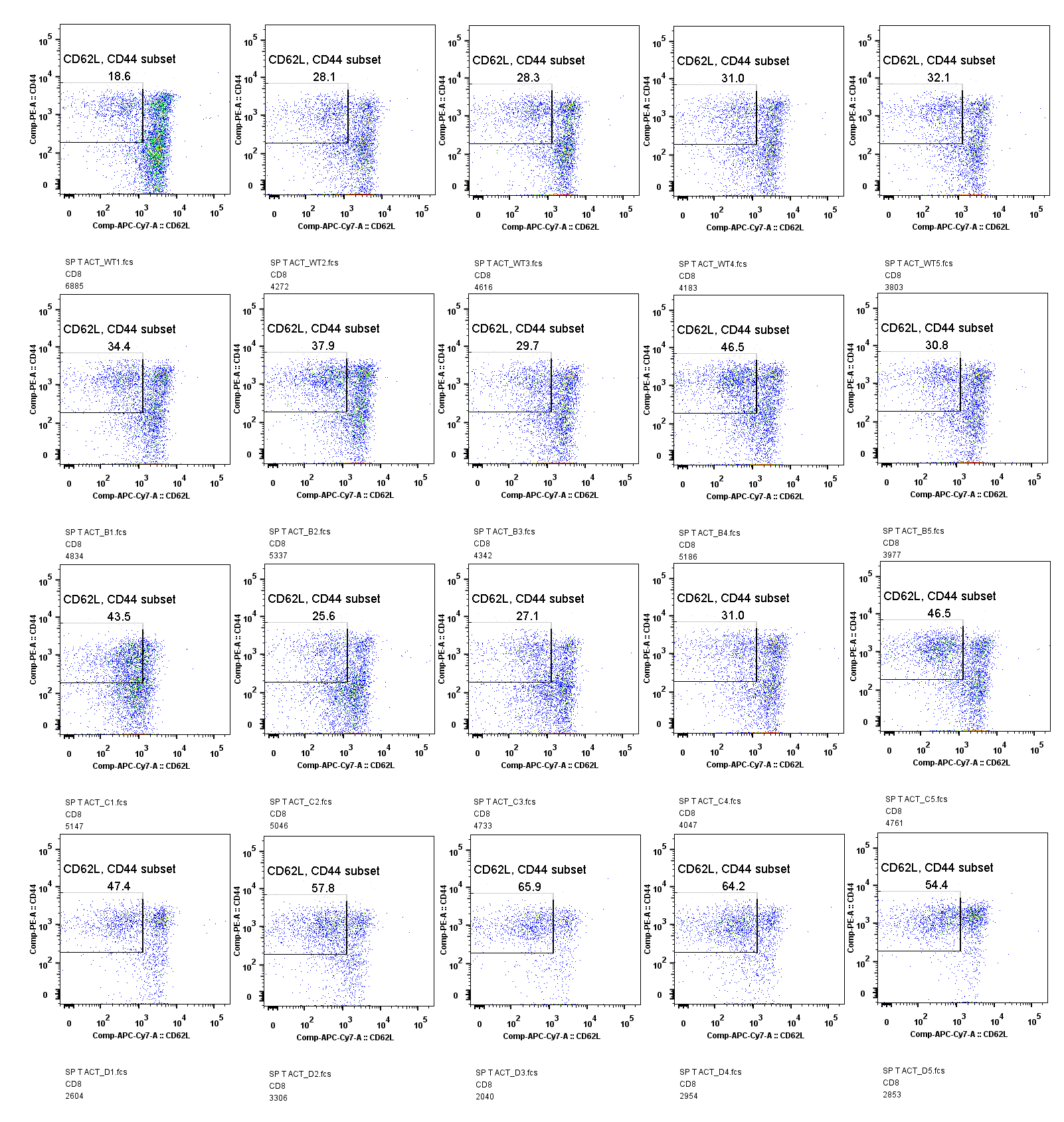


**Figure 4D**


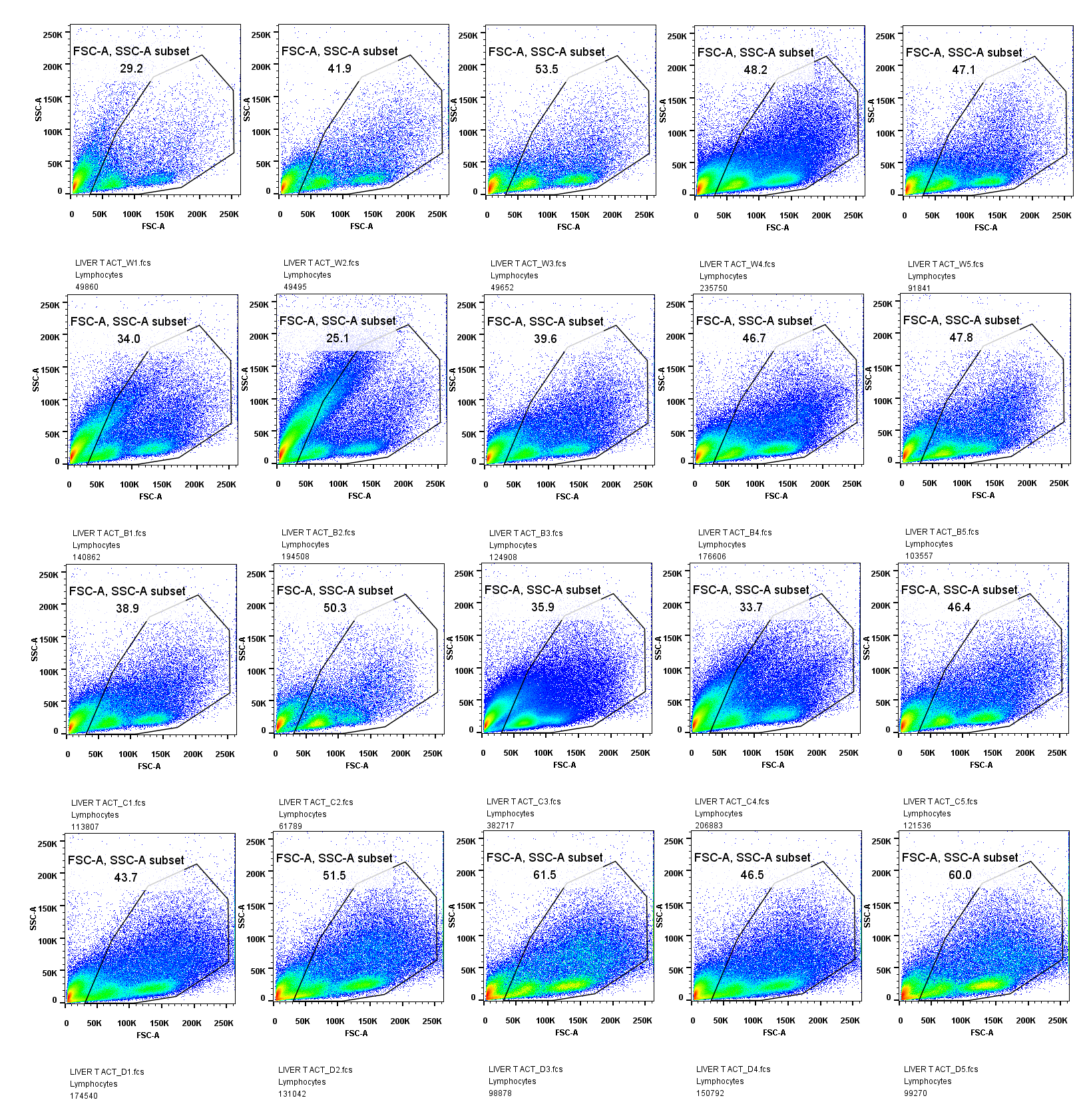


**Figure 4D**


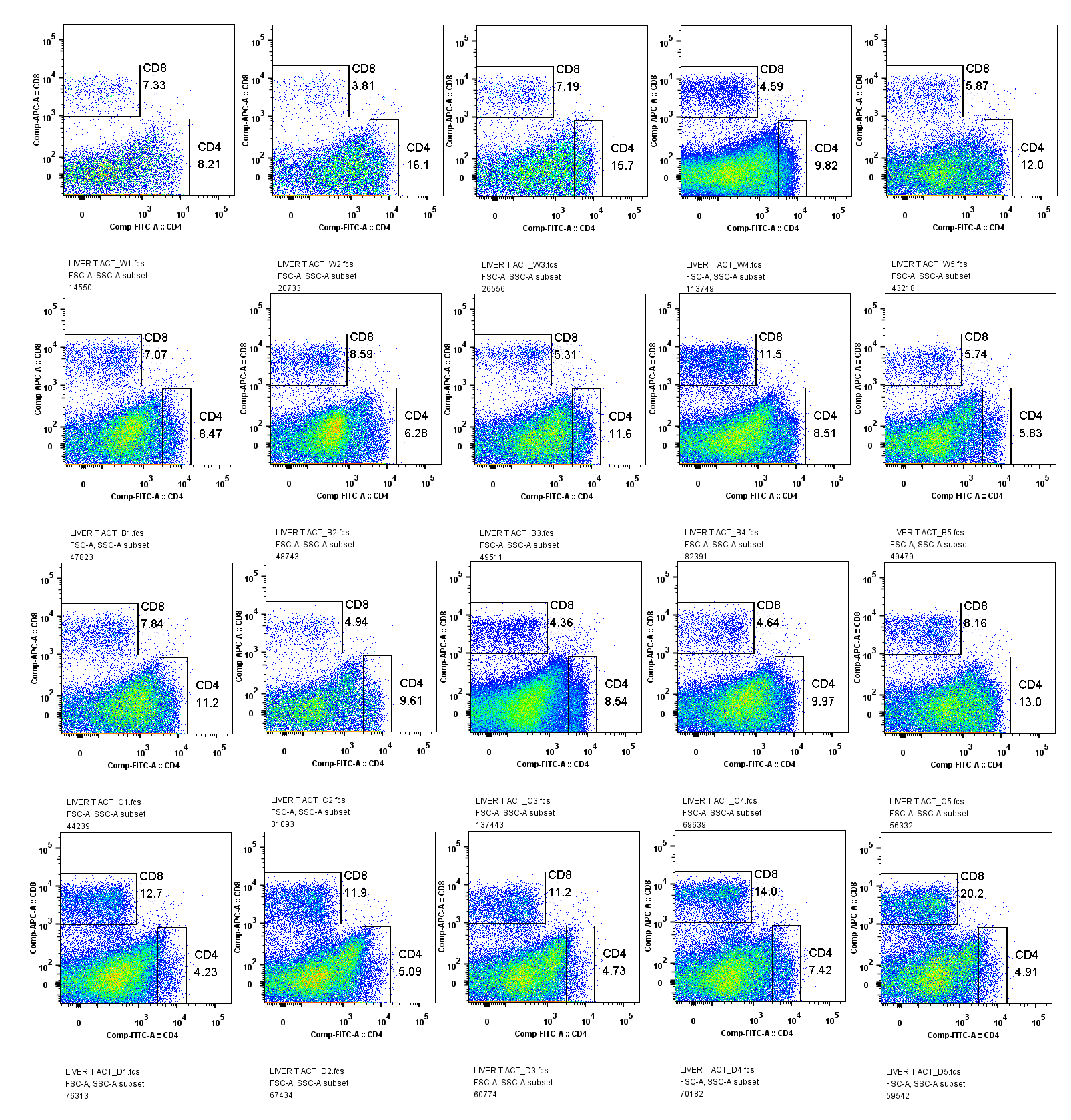


**Figure 4D**


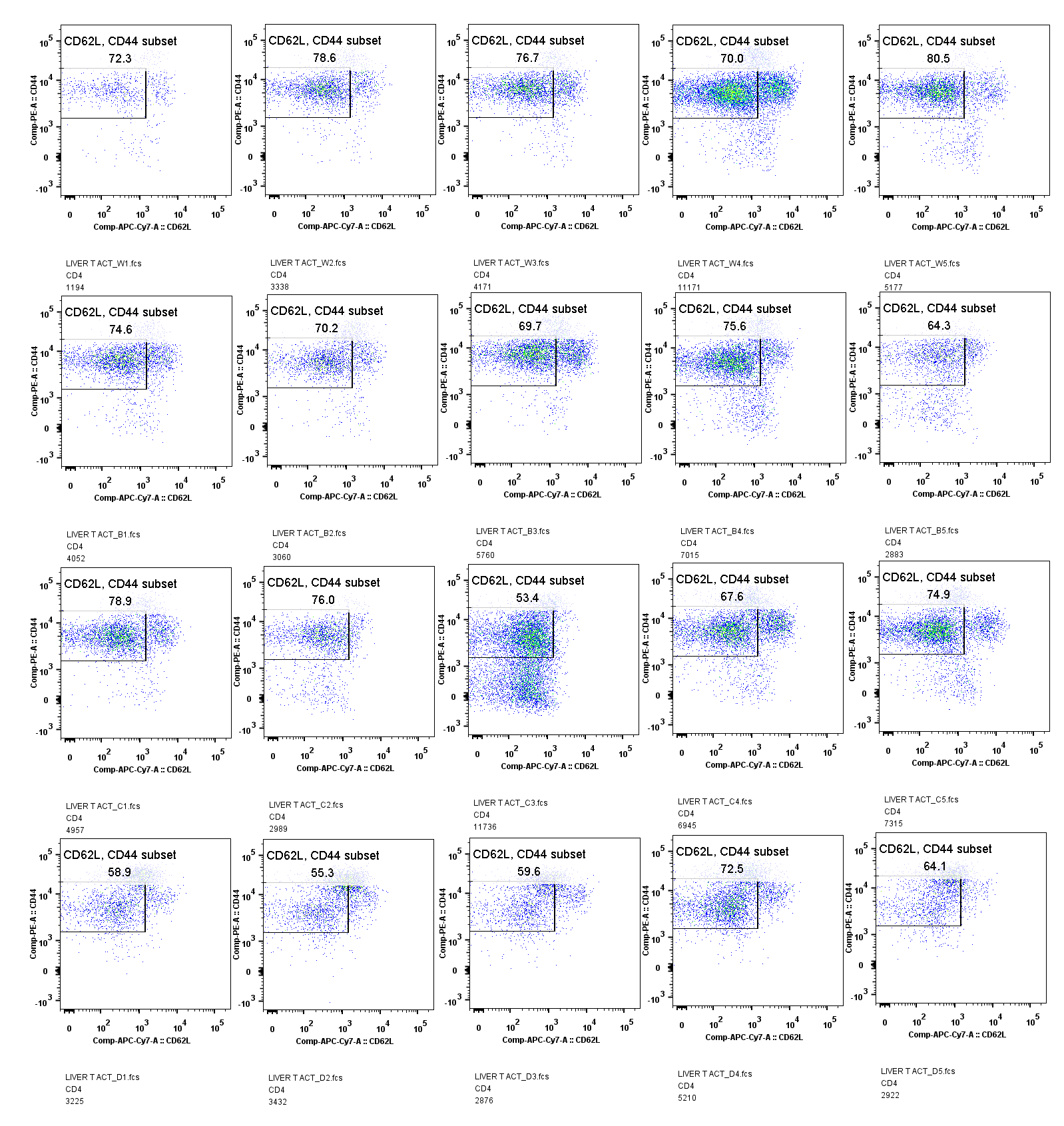


**Figure 4D**


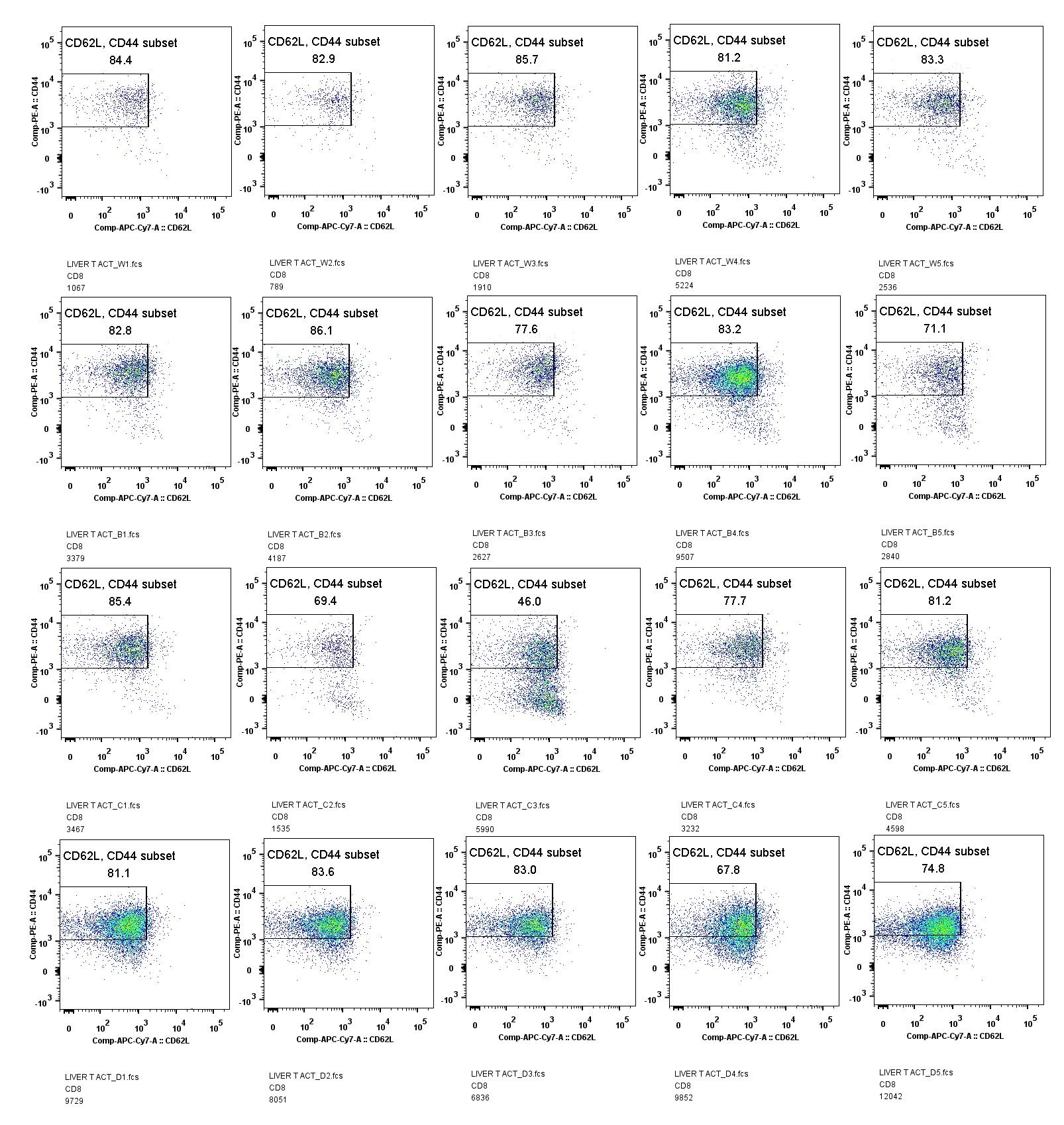


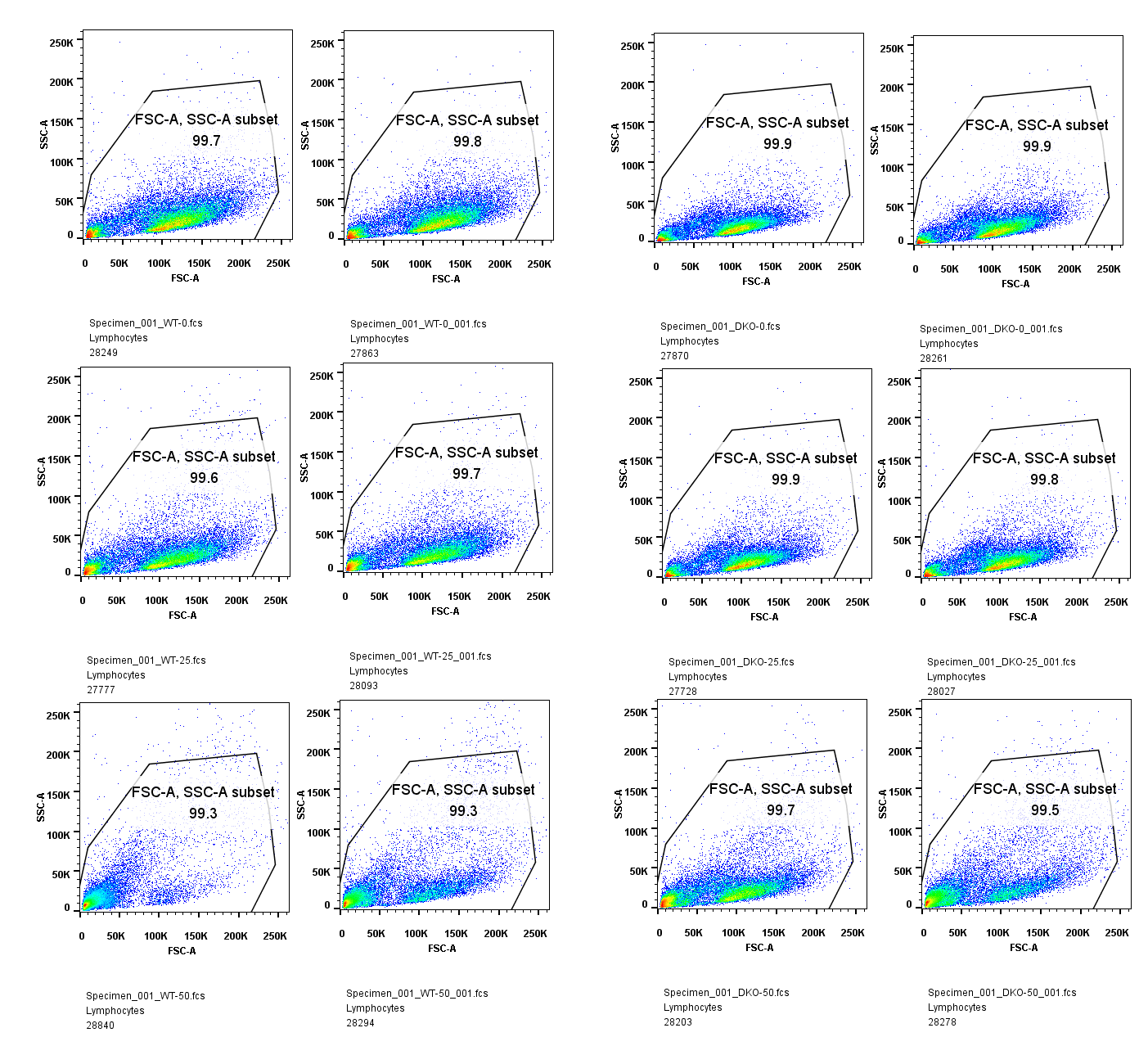
**Figure 5G**

**Figure 5G**


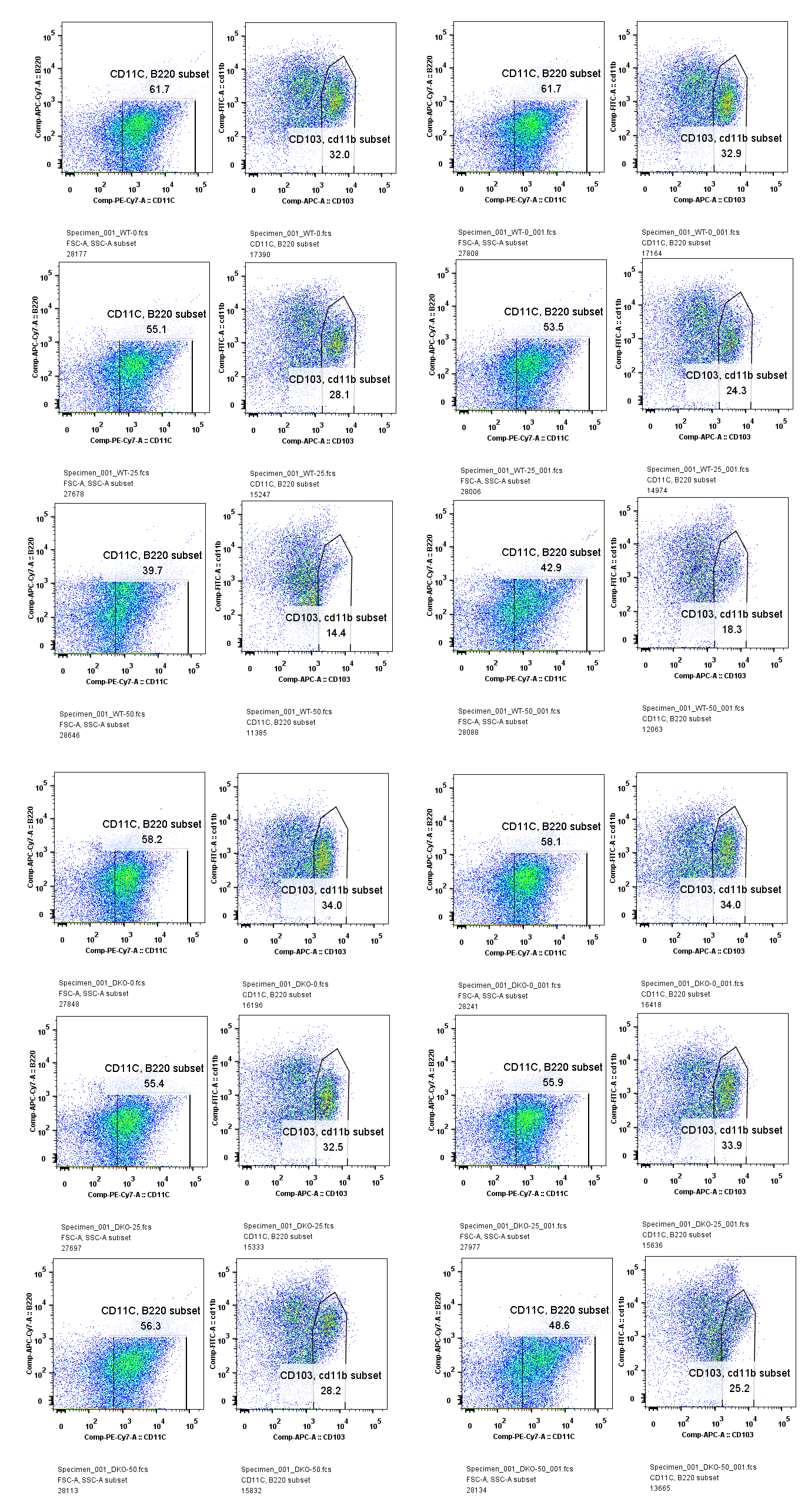


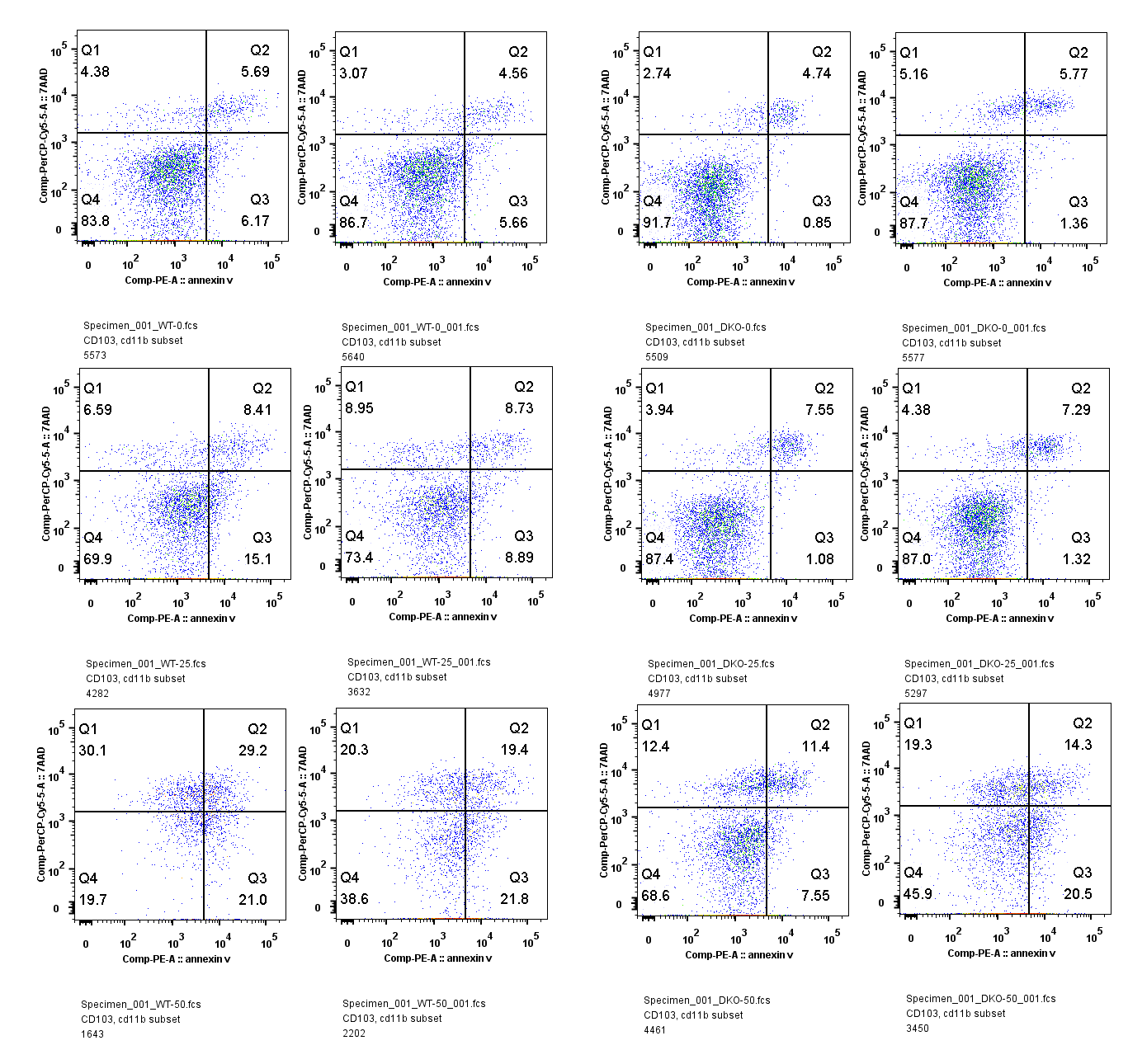
**Figure 5G**


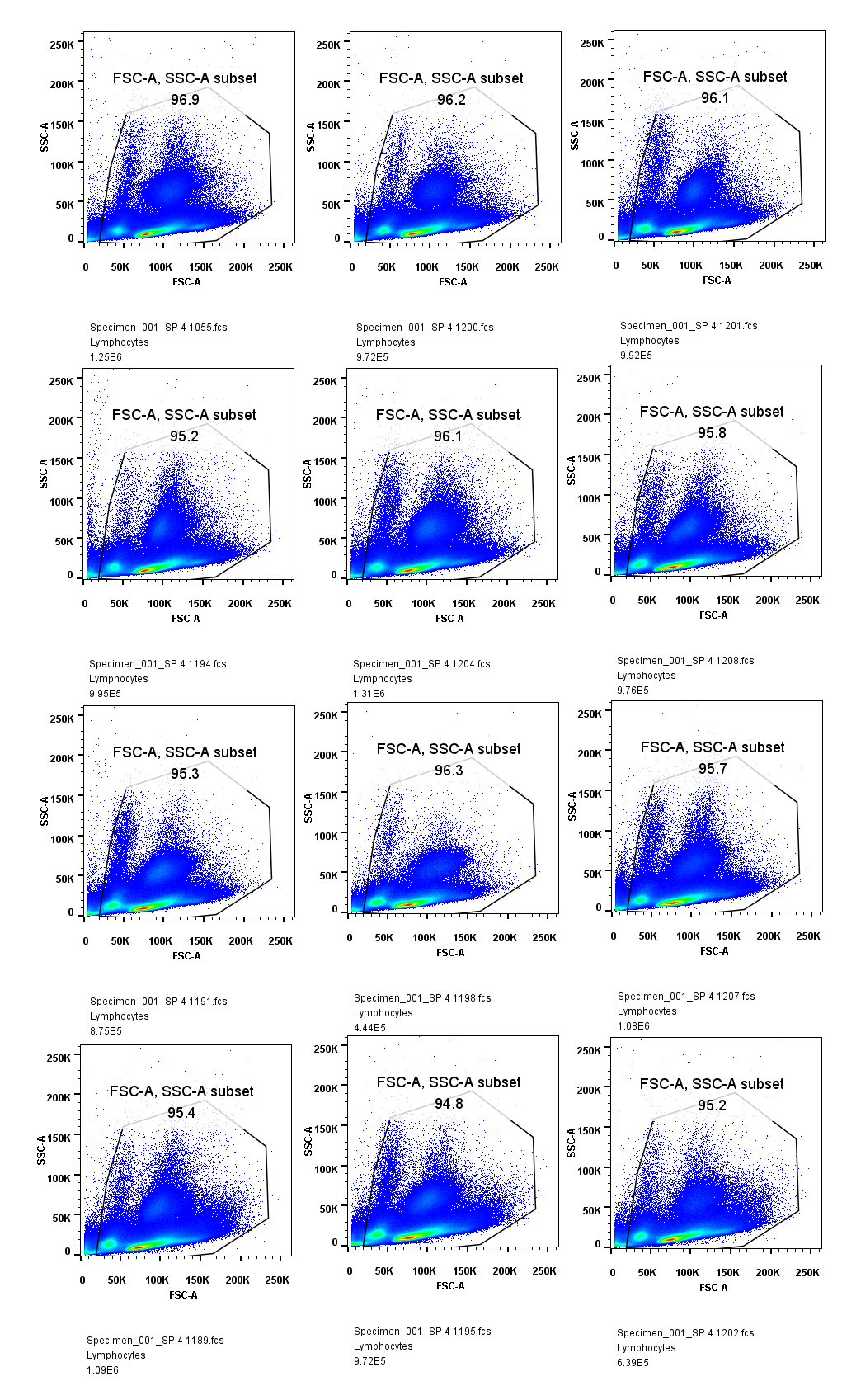
**Supplementary Figure 2A**


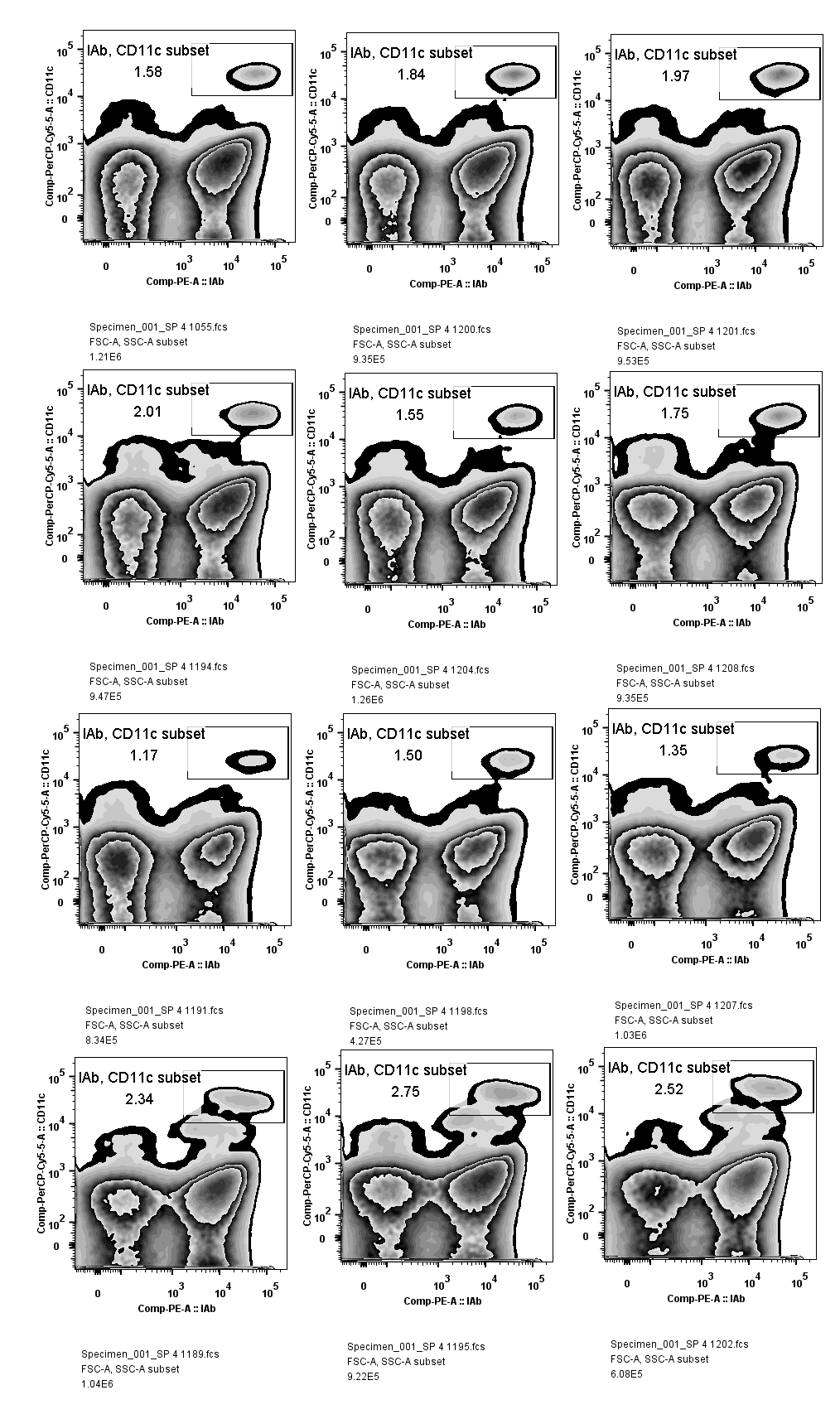
**Supplementary Figure 2A**


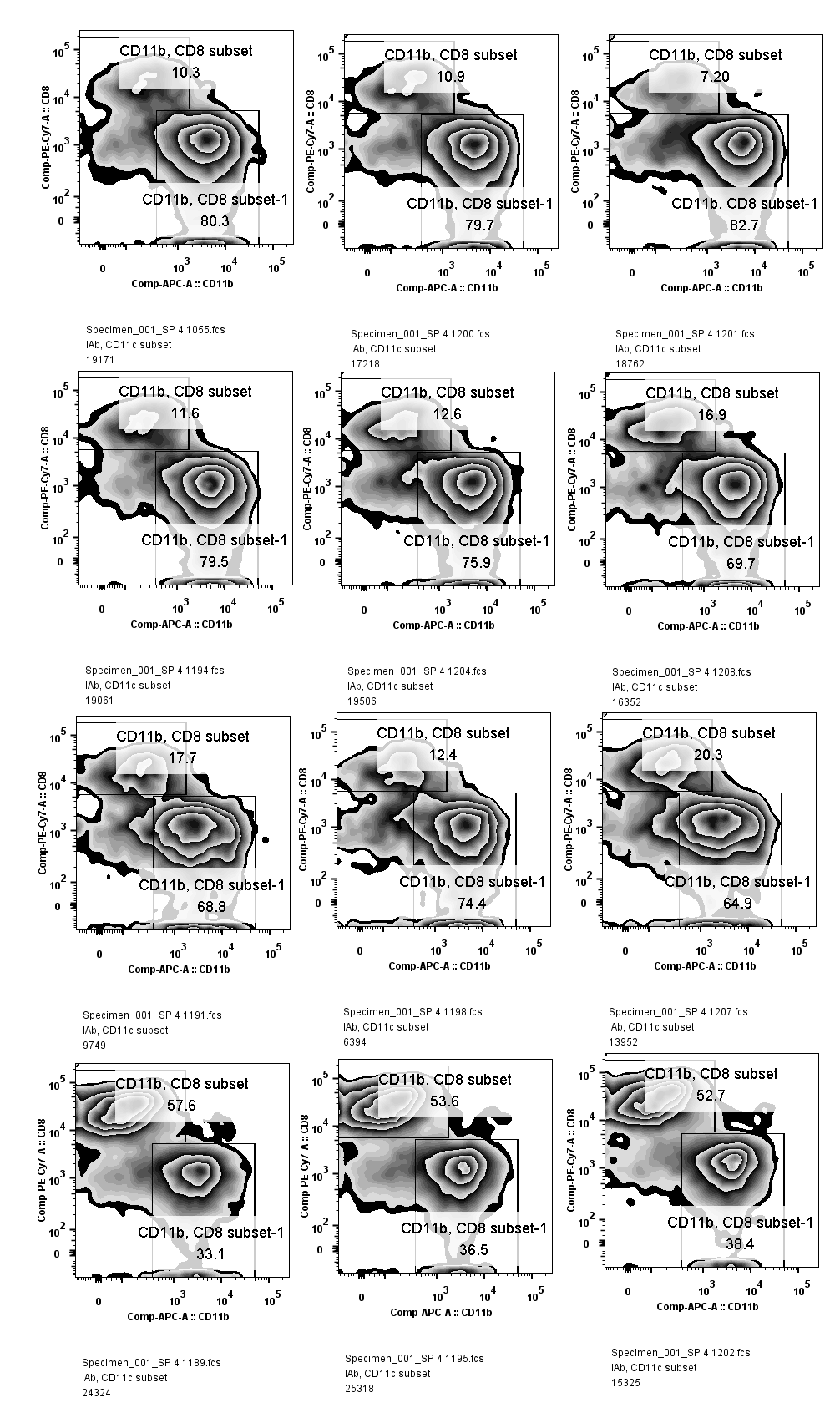
**Supplementary Figure 2A**

**Supplementary Figure 2A**

**WT 1055 1200 1201**

**Cbl-b KO 1194 1204 1208**

**c-Cbl cKO 1191 1198 1207**

**dKO 1189 1195 1202**


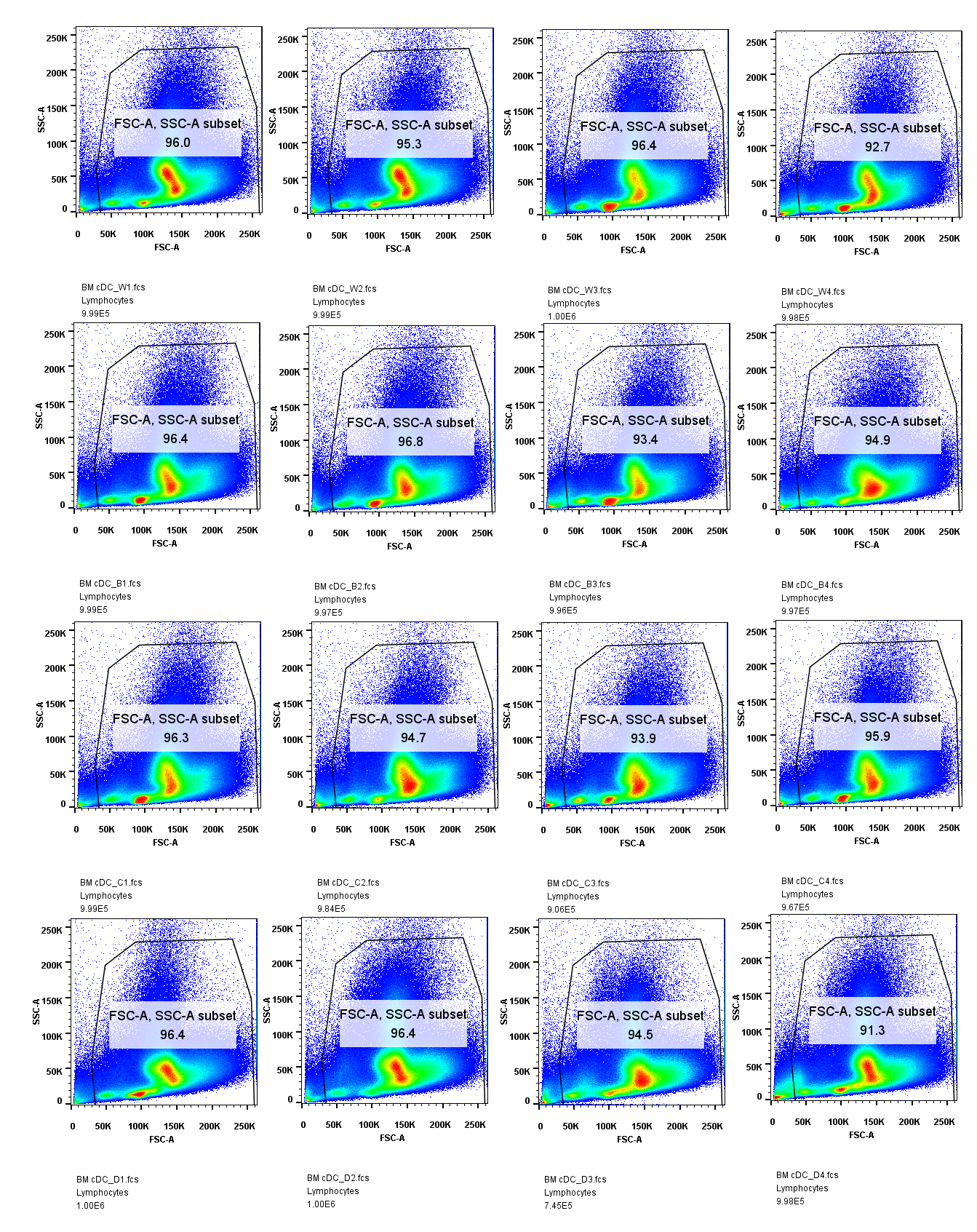
**Supplementary Figure 3A**


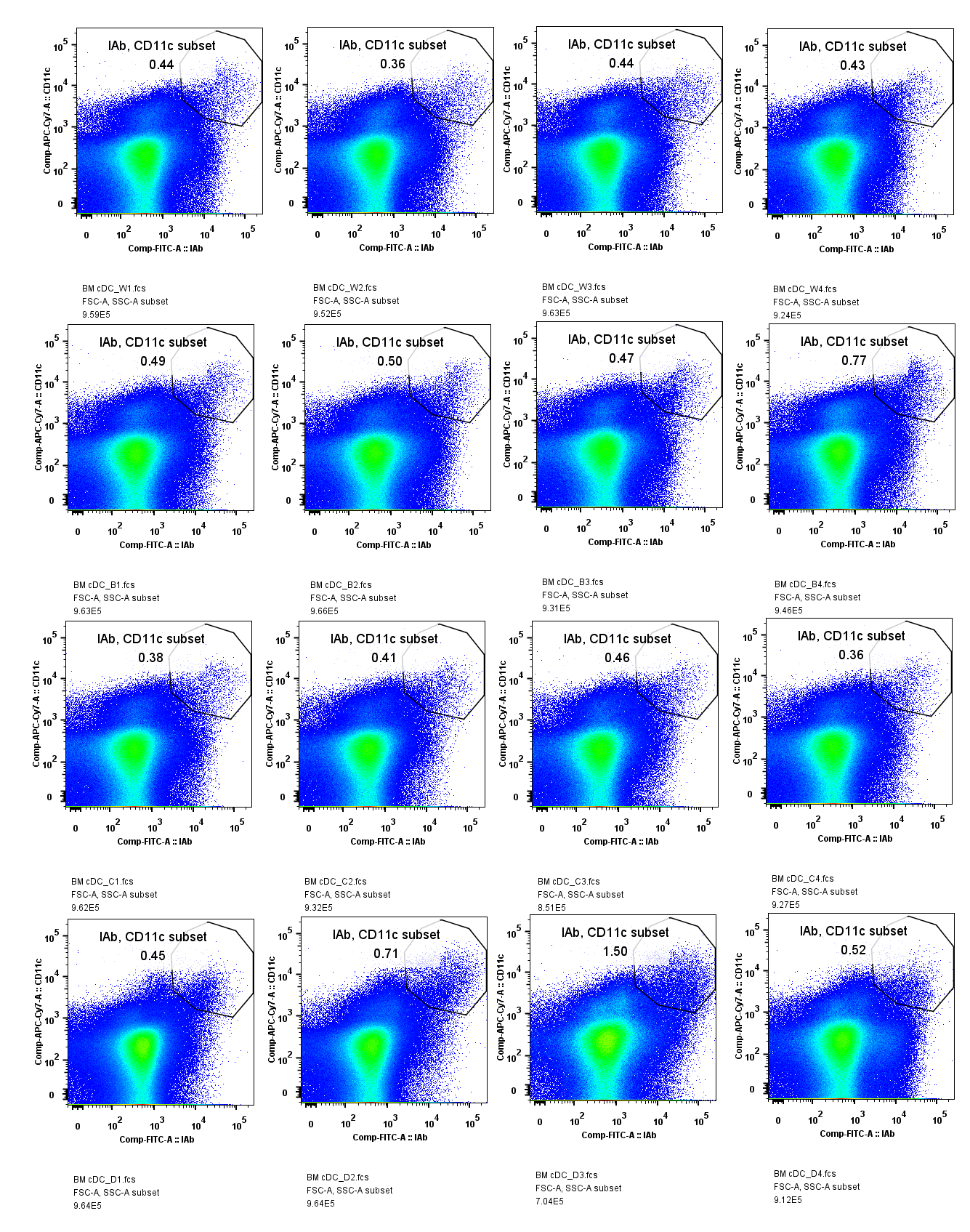
**Supplementary Figure 3A**


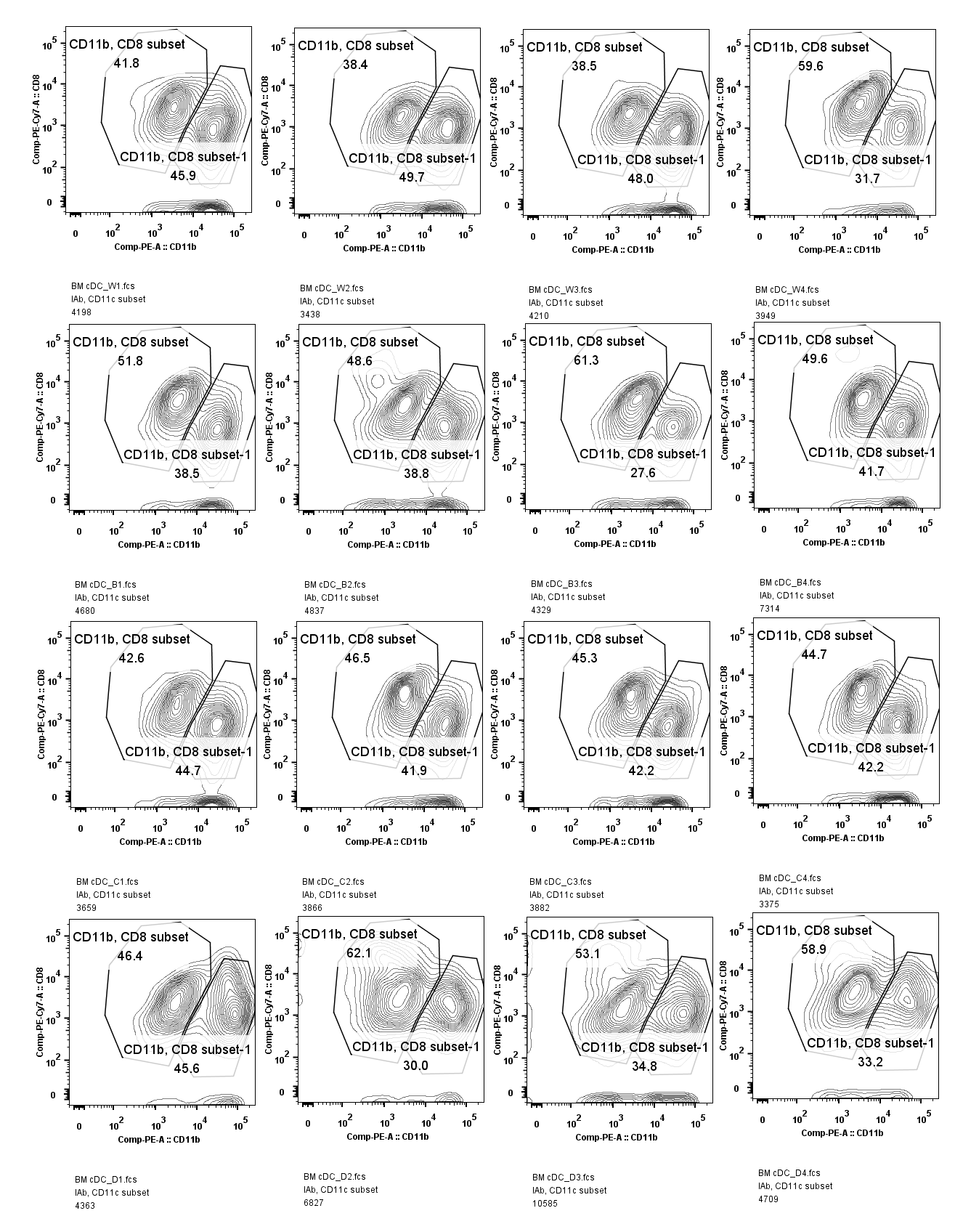
**Supplementary Figure 3A**


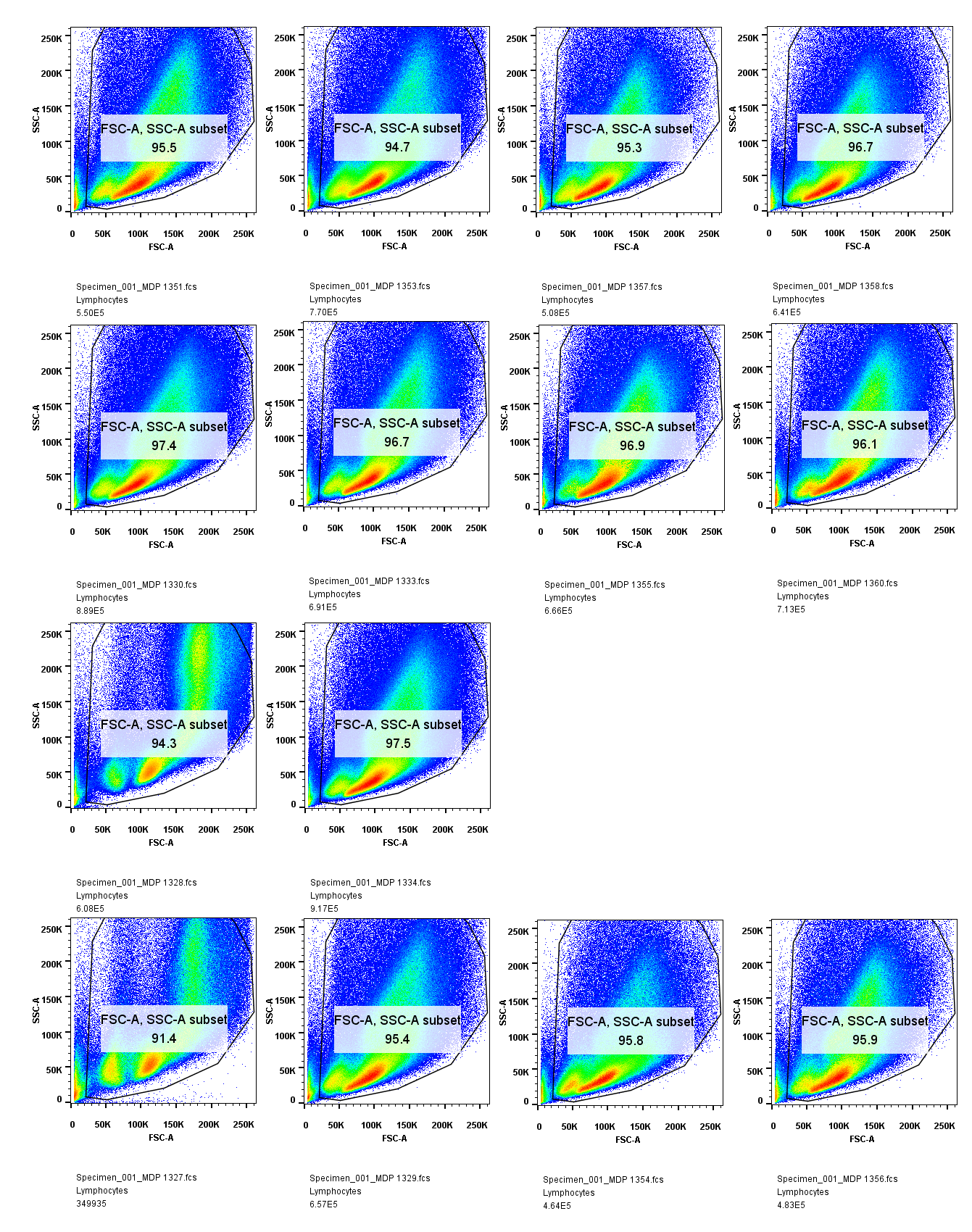
**Supplementary Figure 4A**


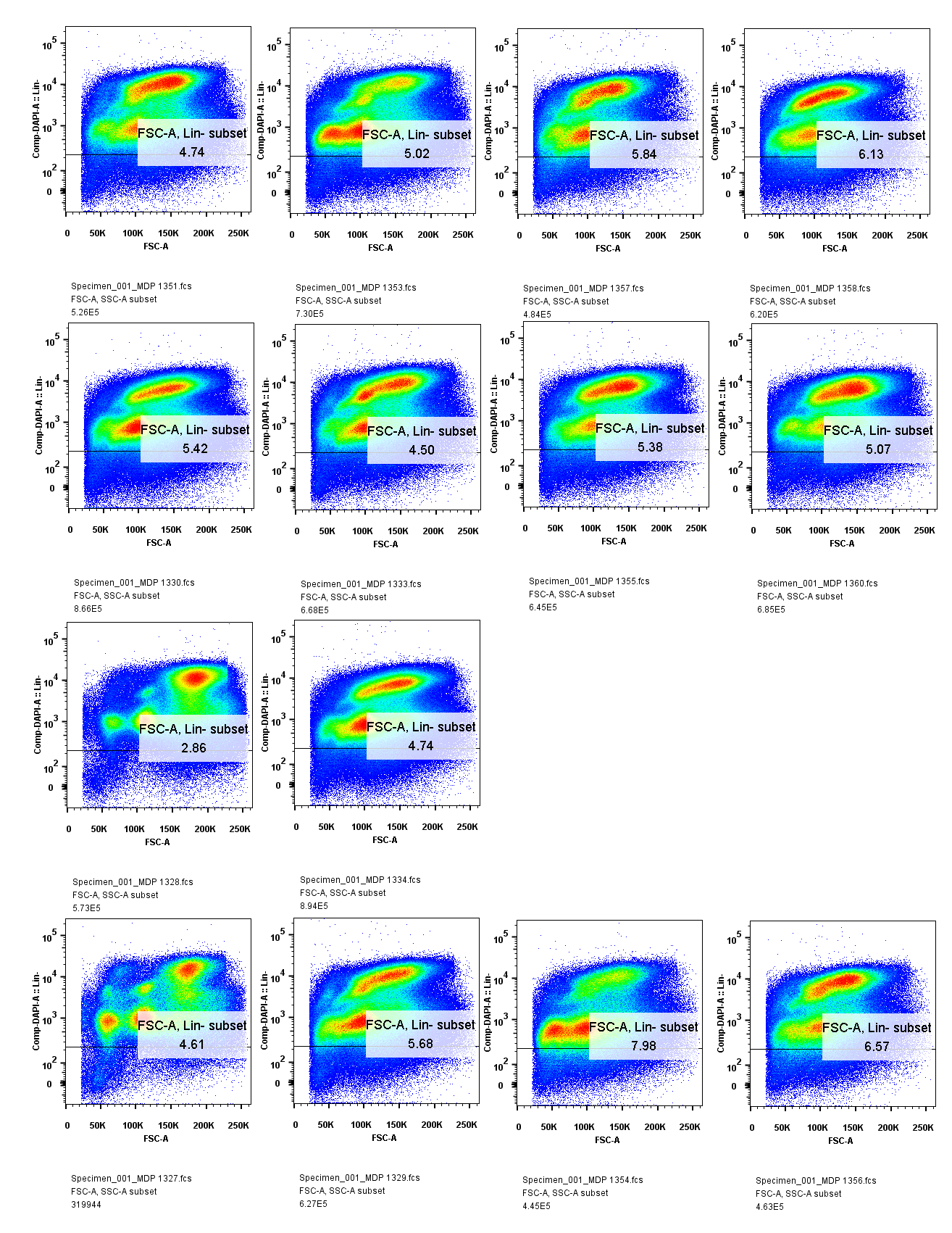
**Supplementary Figure 4A**


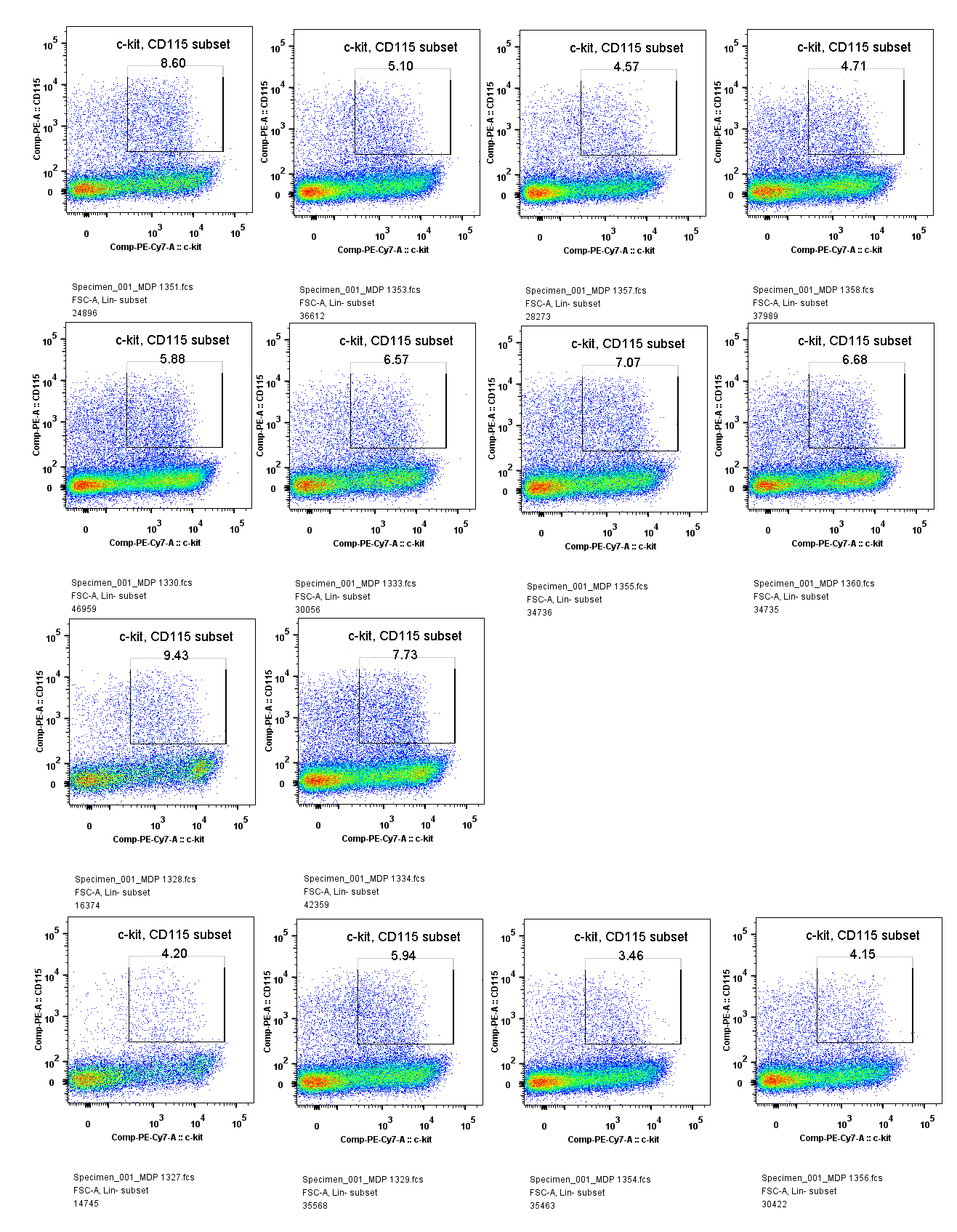
**Supplementary Figure 4A**


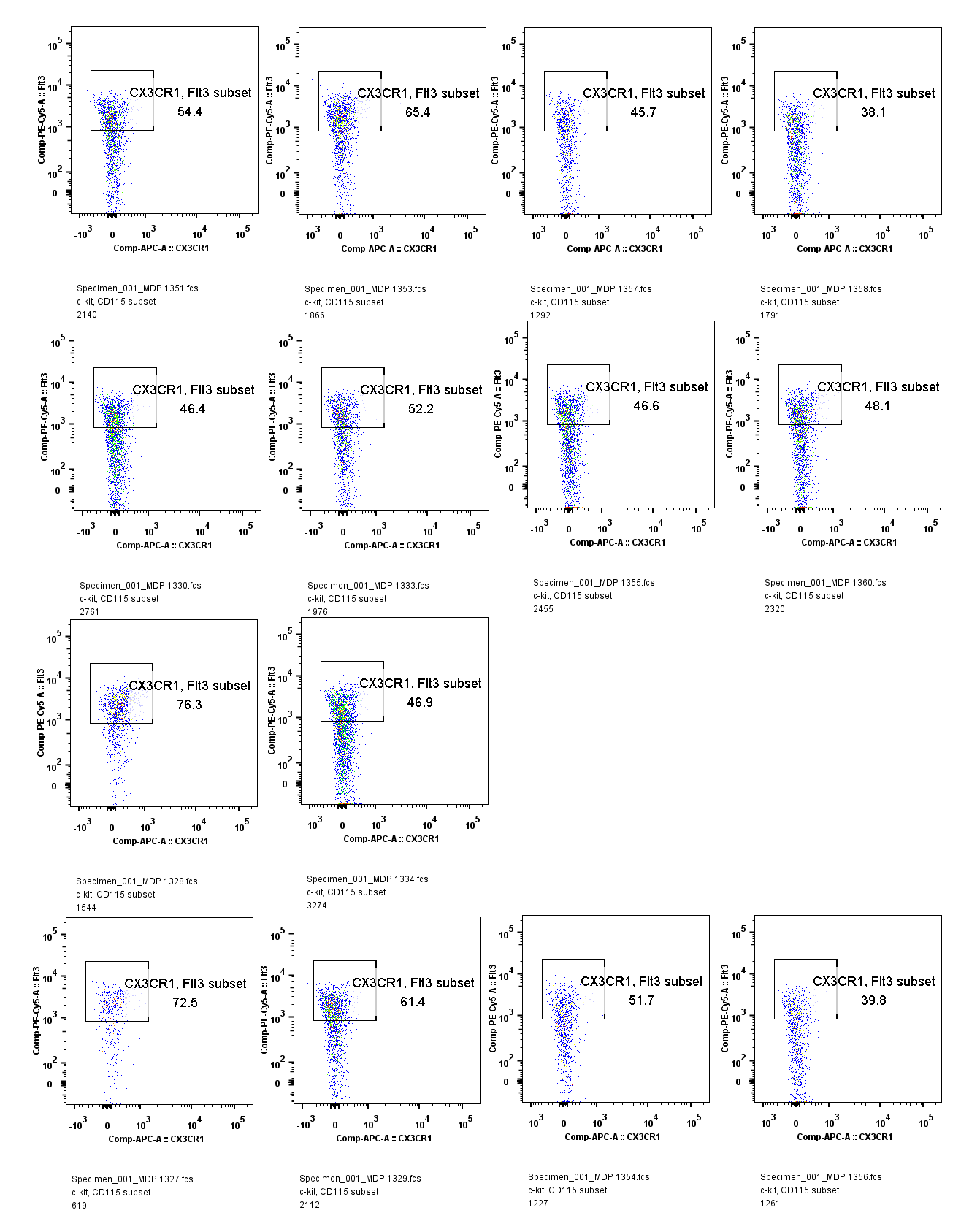
**Supplementary Figure 4A**

**WT 1351 1353 1357 1358**

**Cbl-b KO 1330 1333 1355 1360**

**c-Cbl cKO 1328 1334**

**dKO 1327 1329 1354 1356**


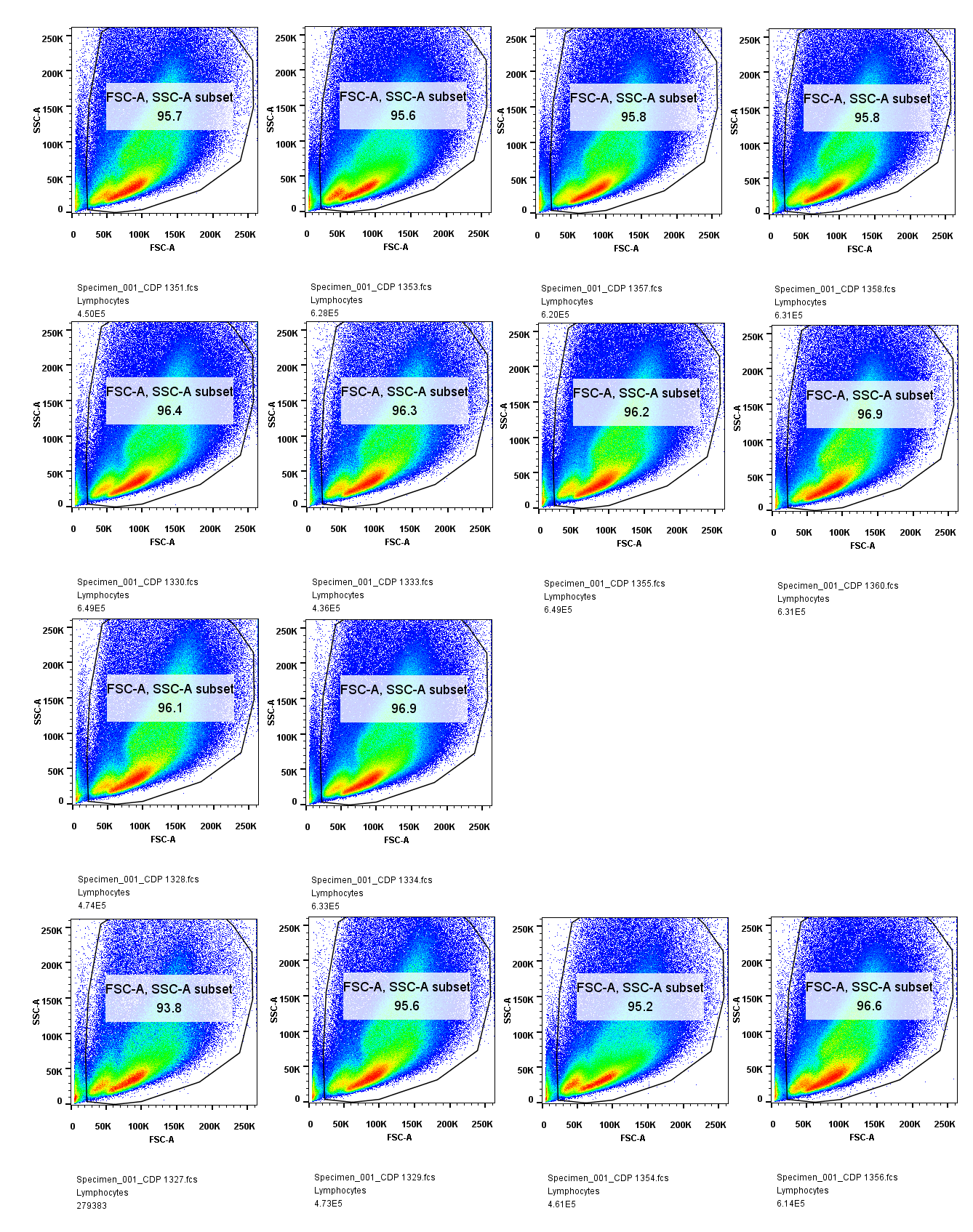
**Supplementary Figure 4C**


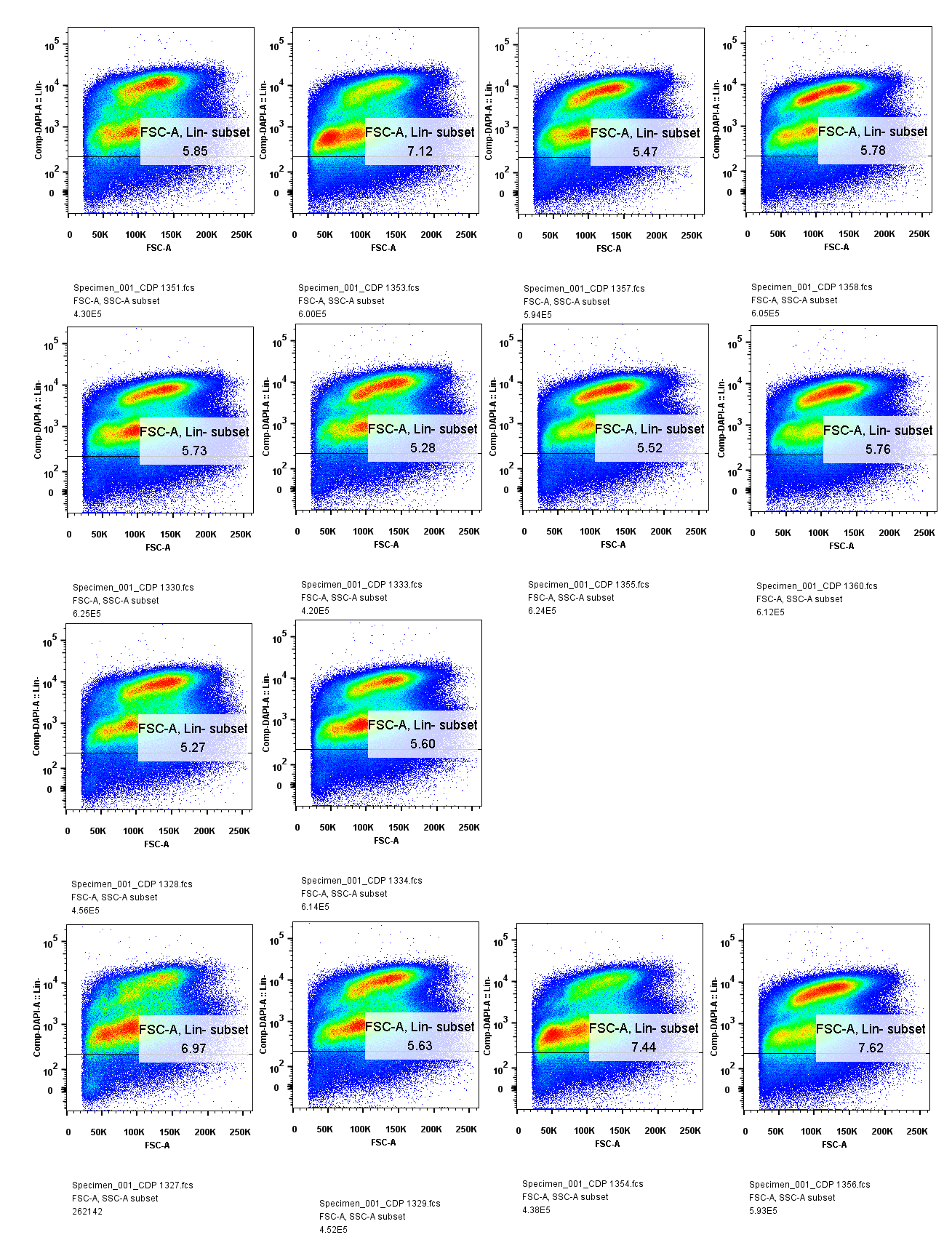
**Supplementary Figure 4C**


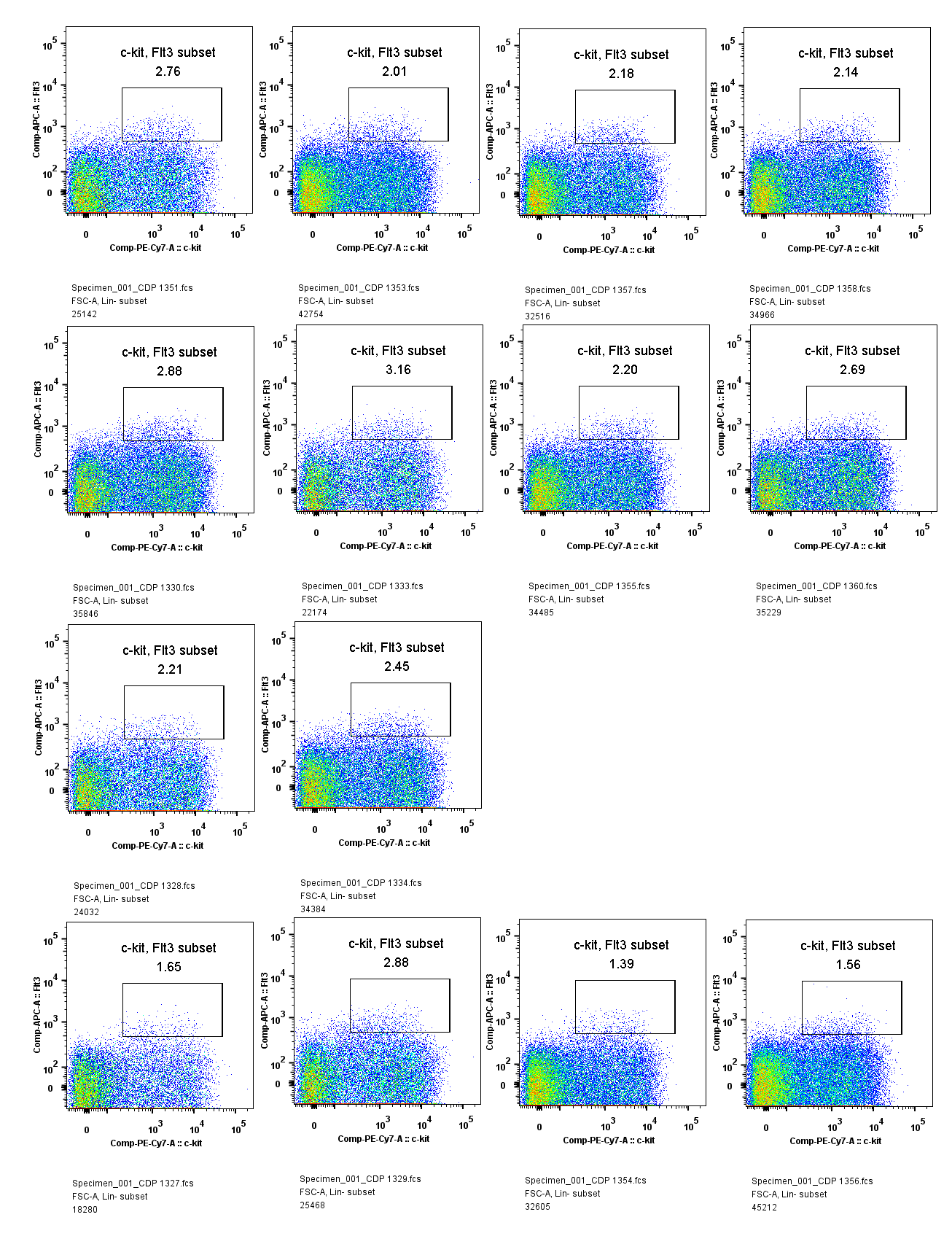
**Supplementary Figure 4C**


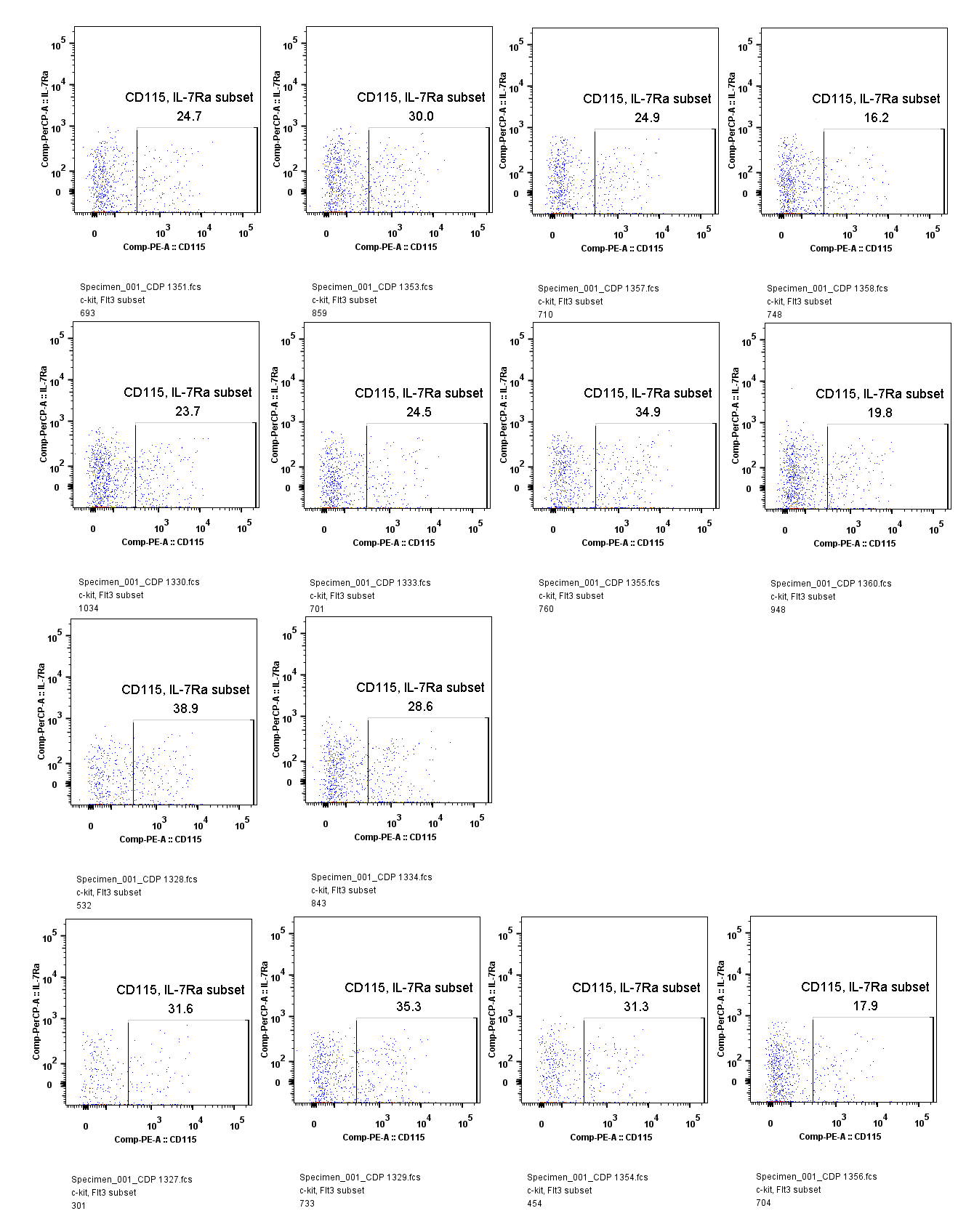
**Supplementary Figure 4C**

**WT 1351 1353 1357 1358**

**Cbl-b KO 1330 1333 1355 1360**

**c-Cbl cKO 1328 1334**

**dKO 1327 1329 1354 1356**


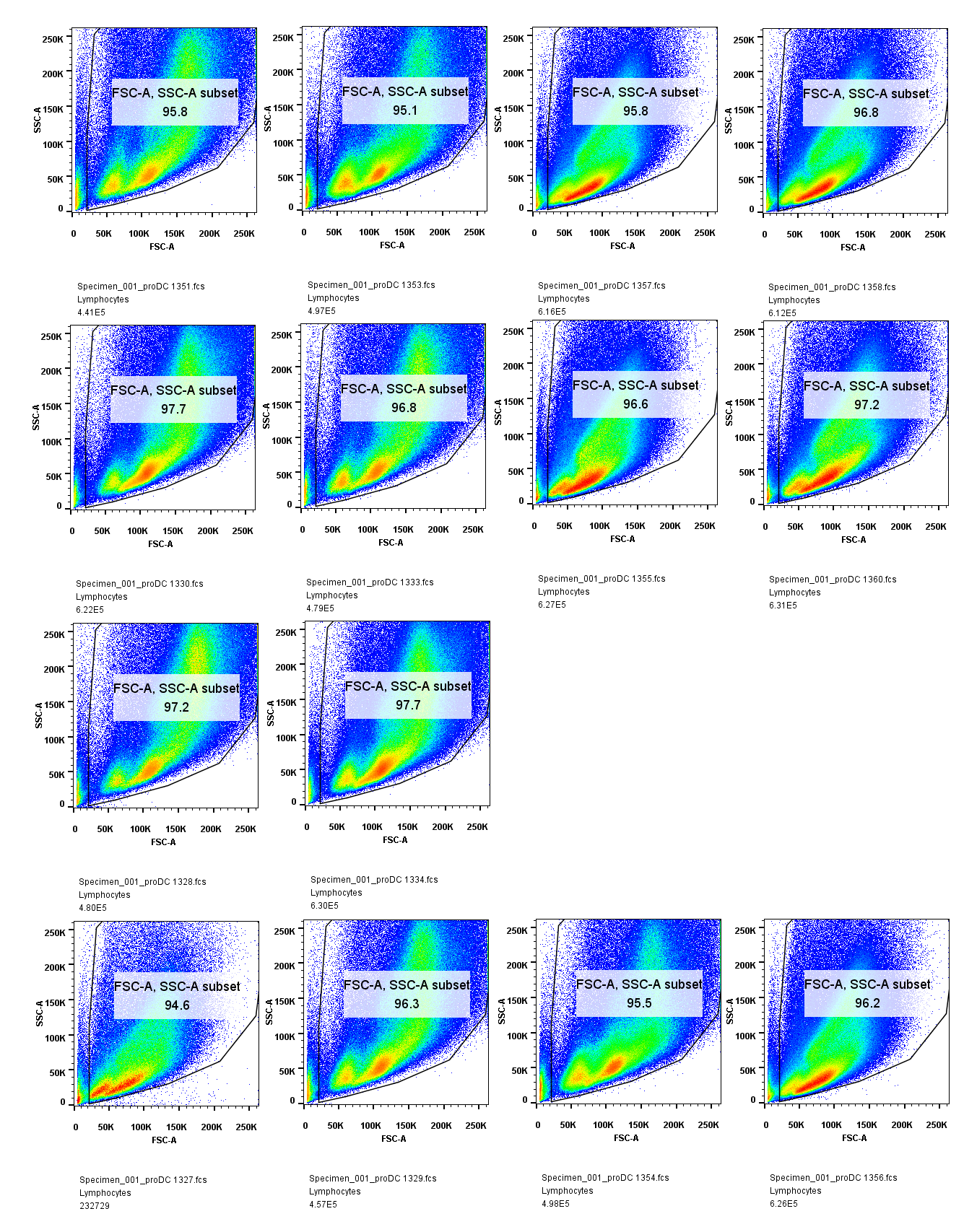
**Supplementary Figure 4E**


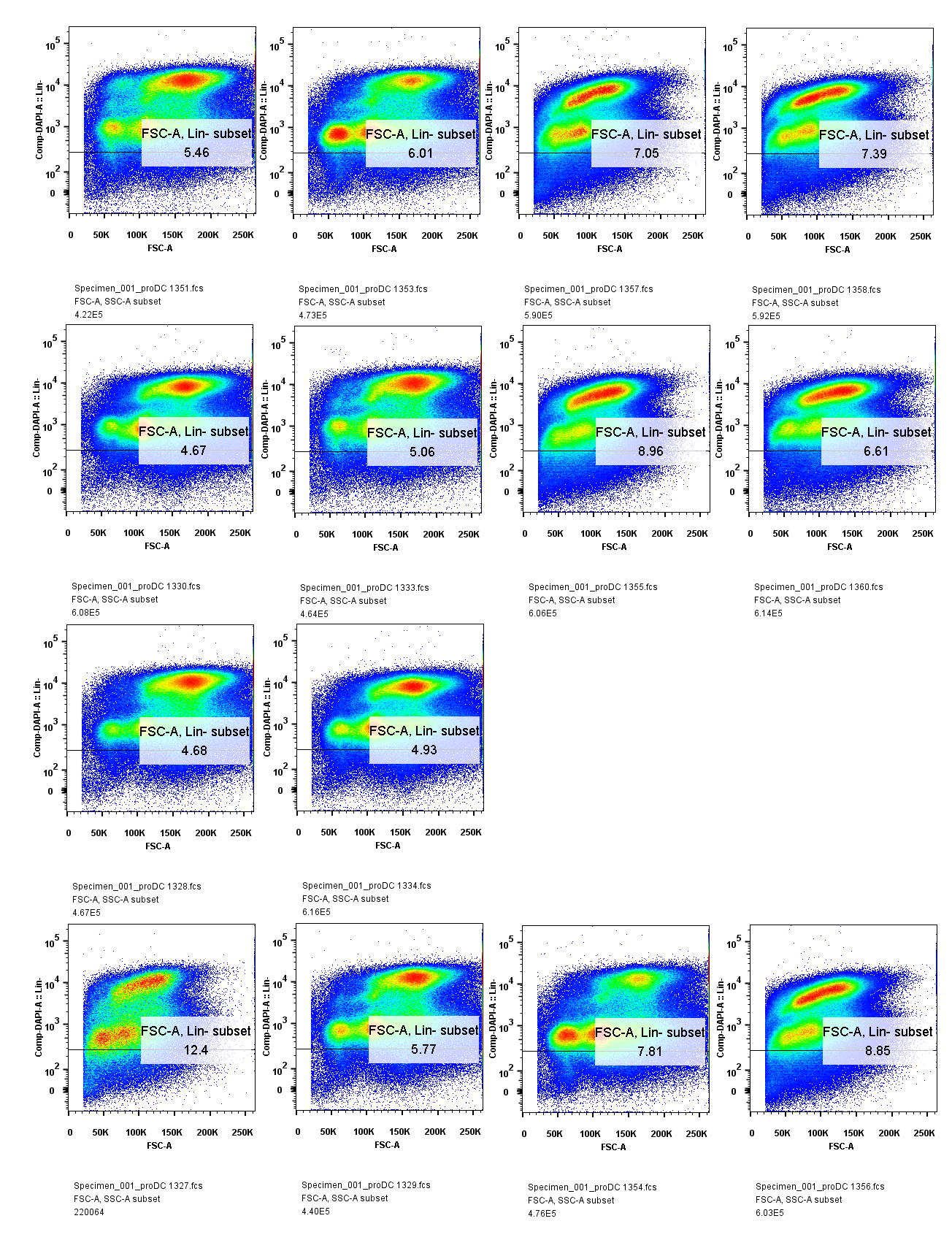
**Supplementary Figure 4E**


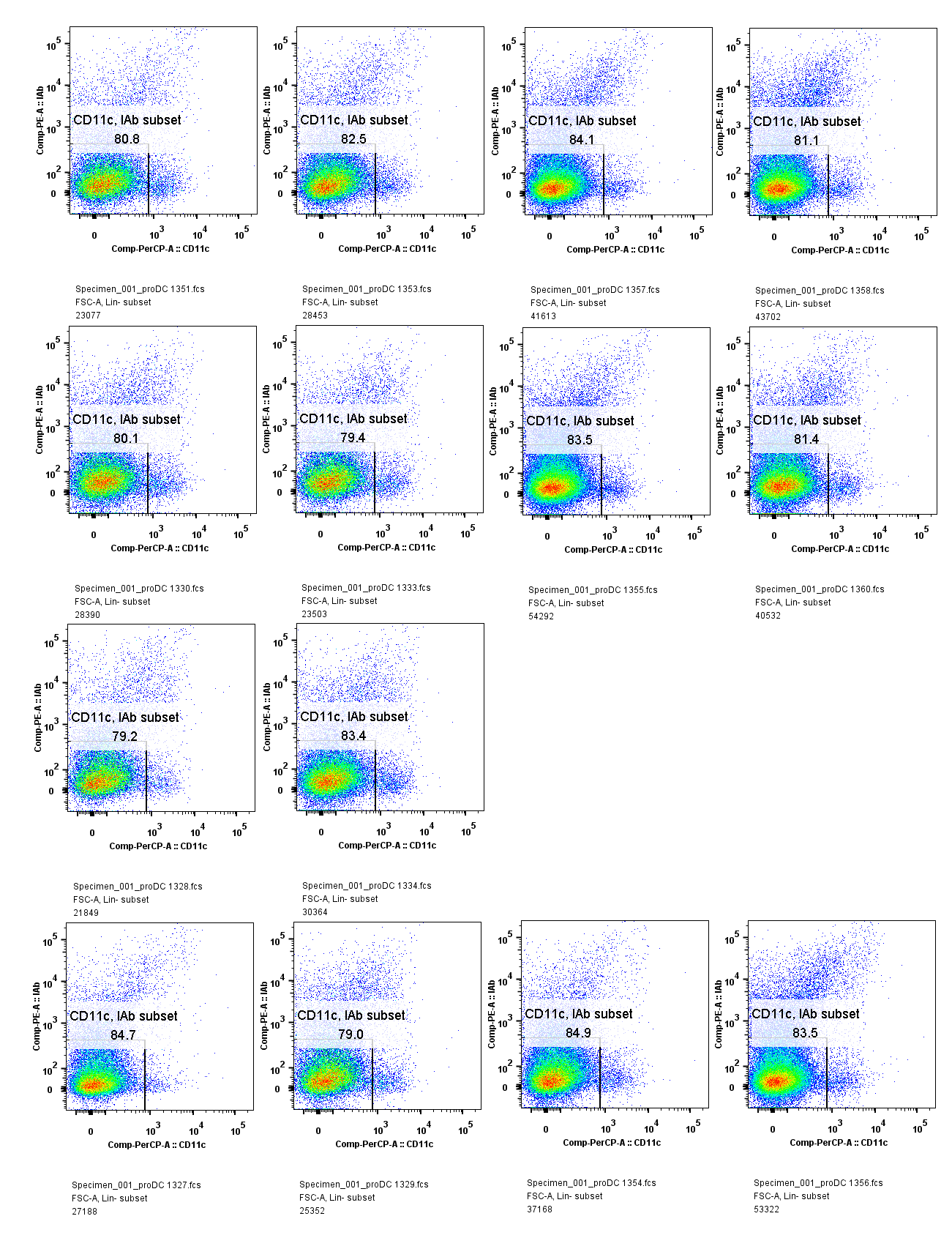
**Supplementary Figure 4E**


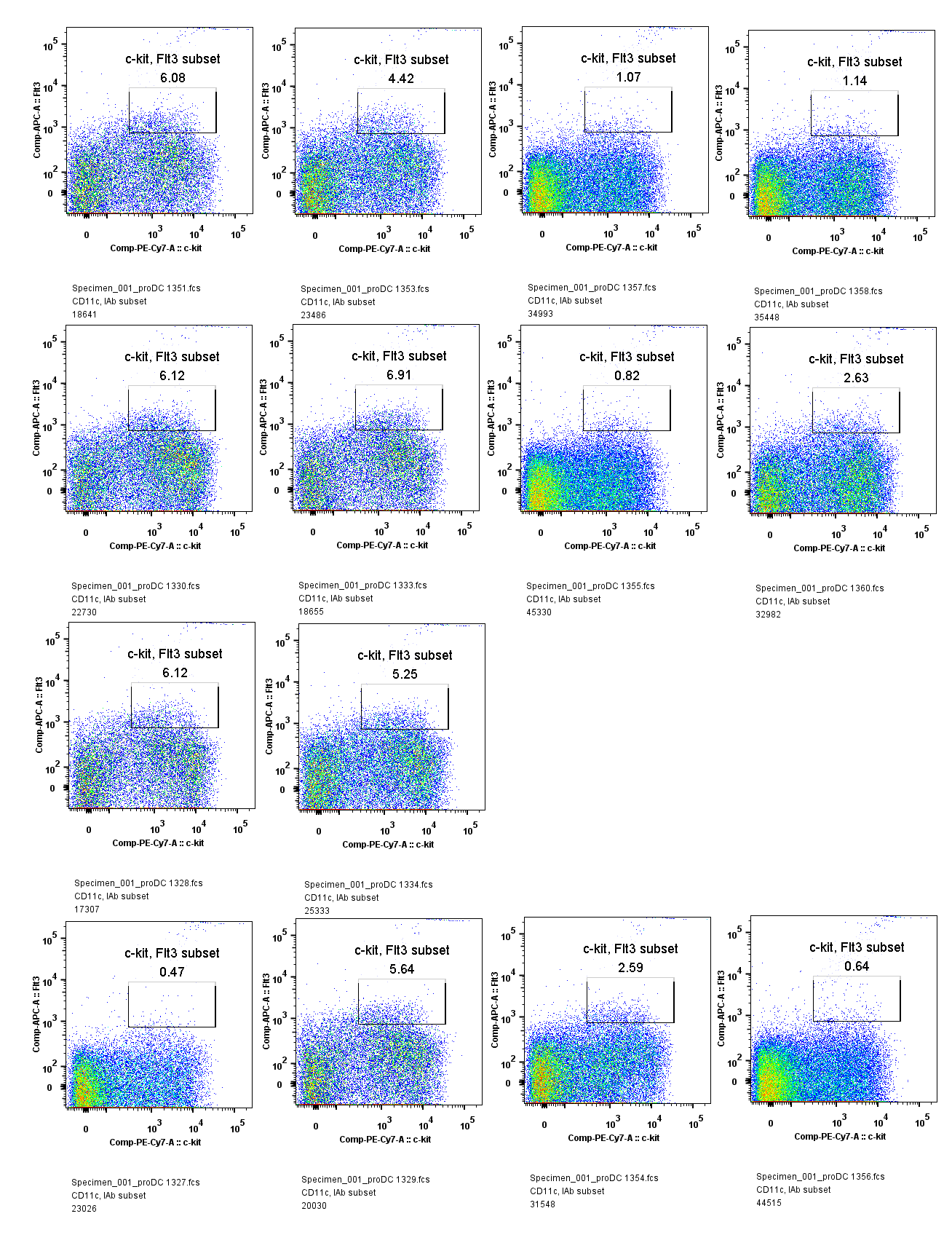
**Supplementary Figure 4E**

**WT 1351 1353 1357 1358**

**Cbl-b KO 1330 1333 1355 1360**

**c-Cbl cKO 1328 1334**

**dKO 1327 1329 1354 1356**


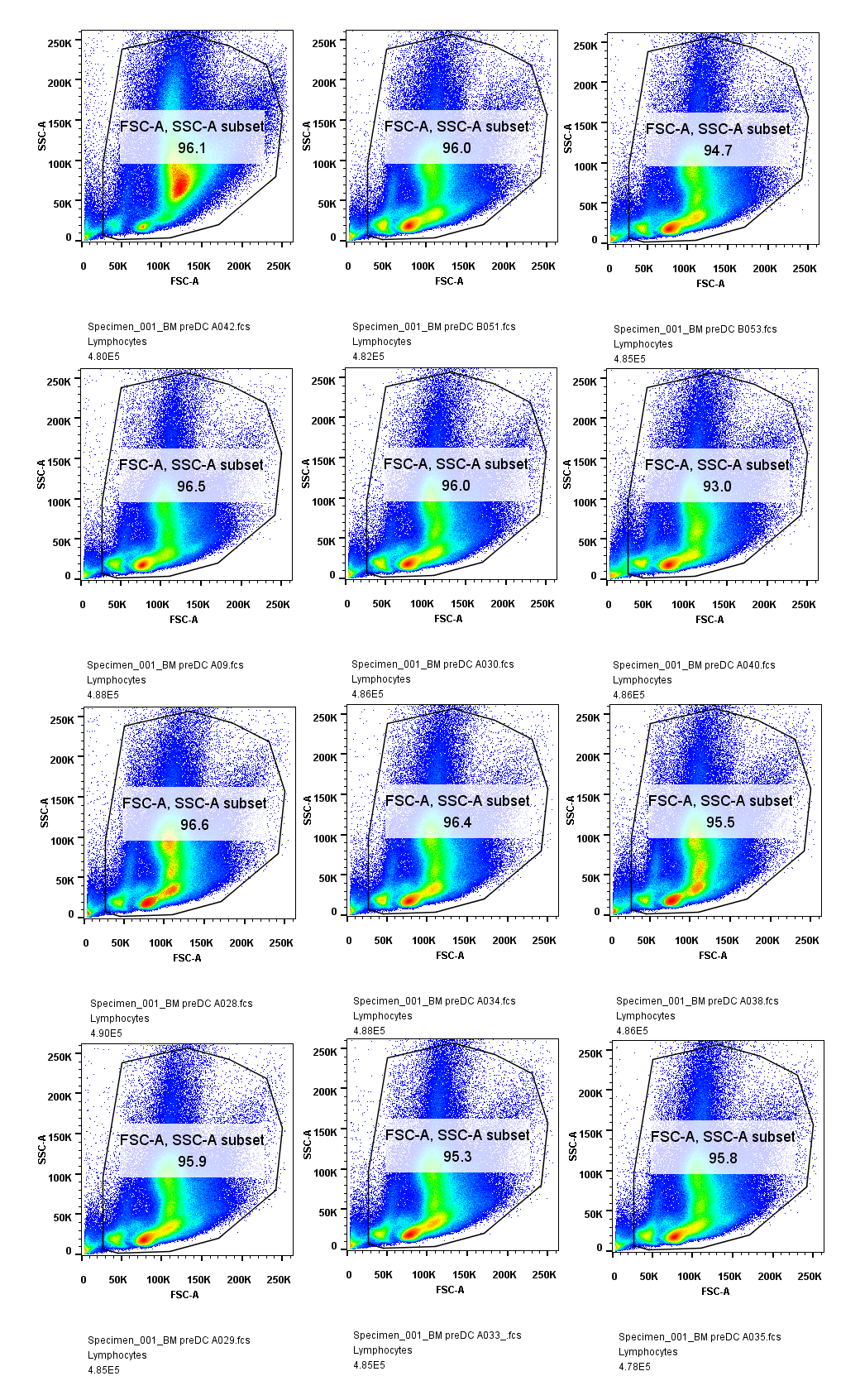
**Supplementary Figure 4G**


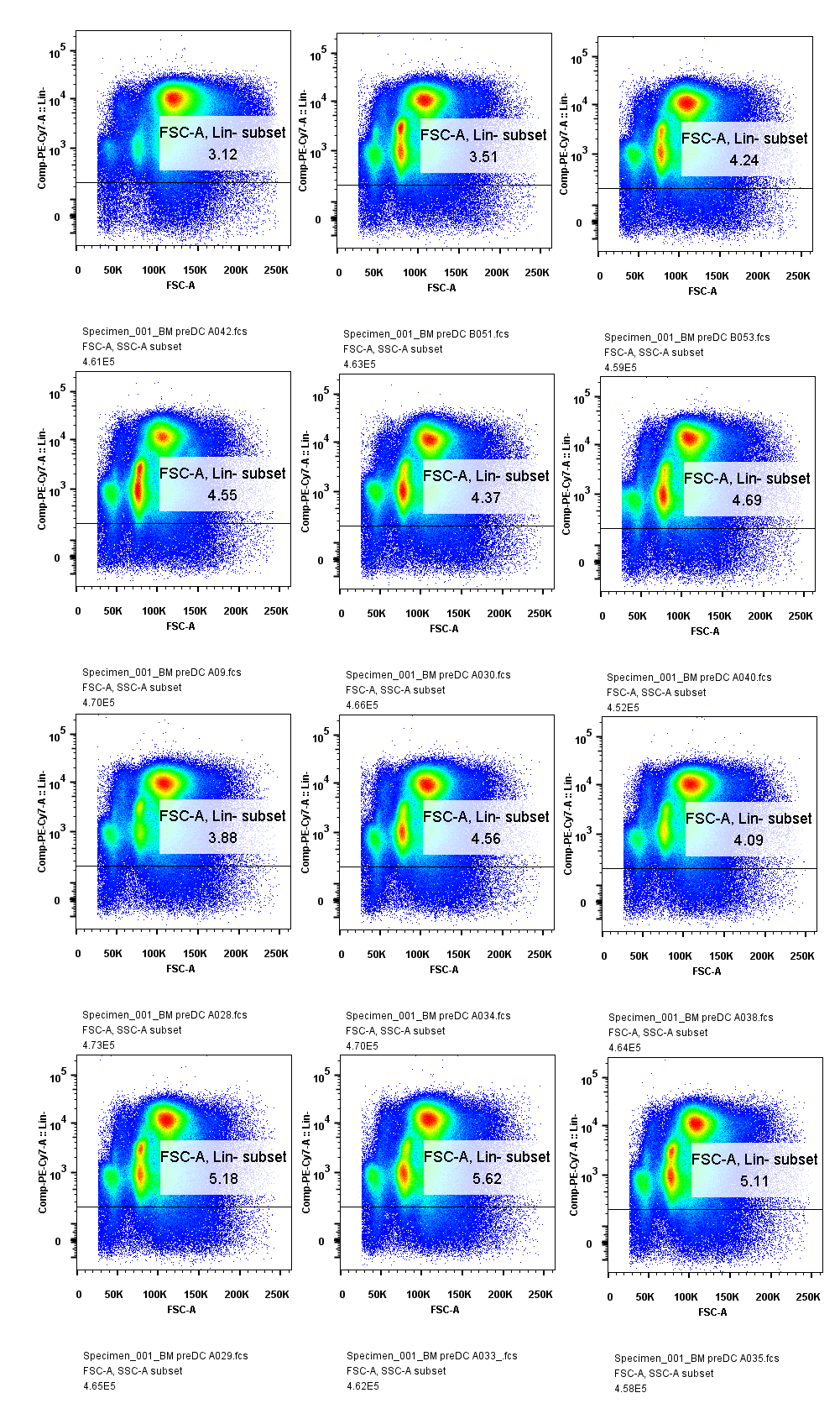
**Supplementary Figure 4G**

**Supplementary Figure 4G**


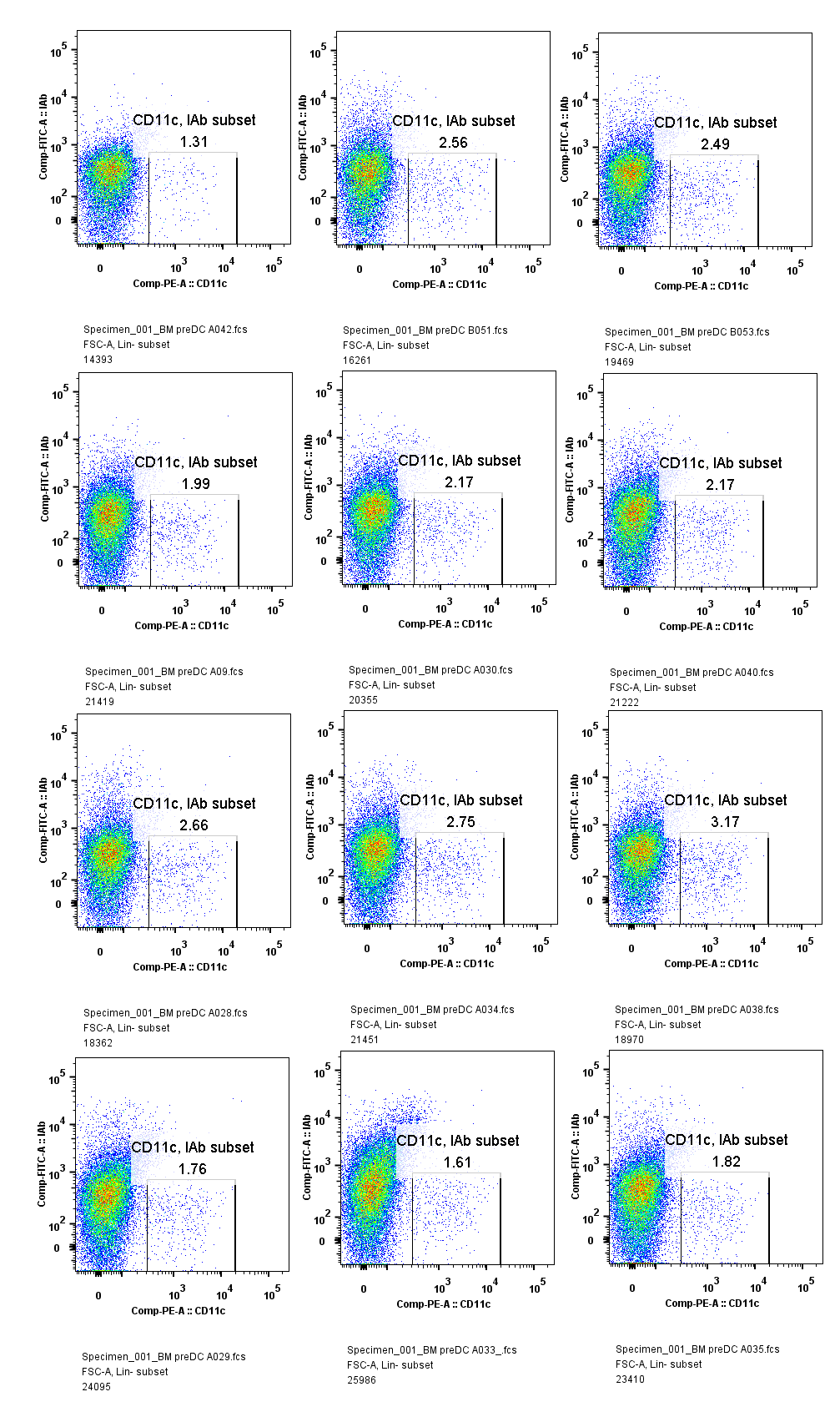


**Supplementary Figure 4G**


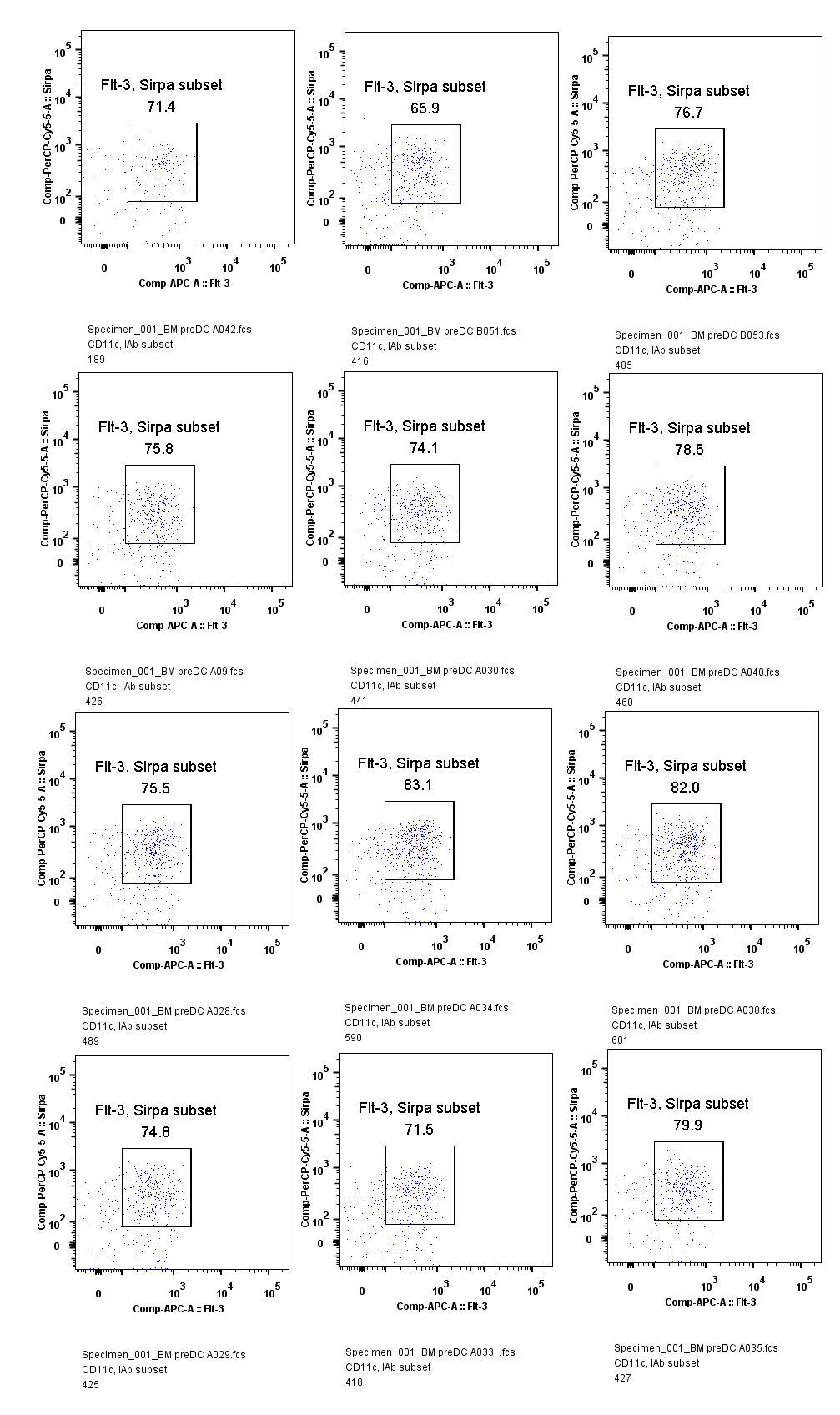


**Supplementary Figure 4G**

**WT A042 B051 B052**

**Cbl-b KO A09 A030 A040**

**c-Cbl cKO A028 A034 A038**

**dKO A029 A033 A035**


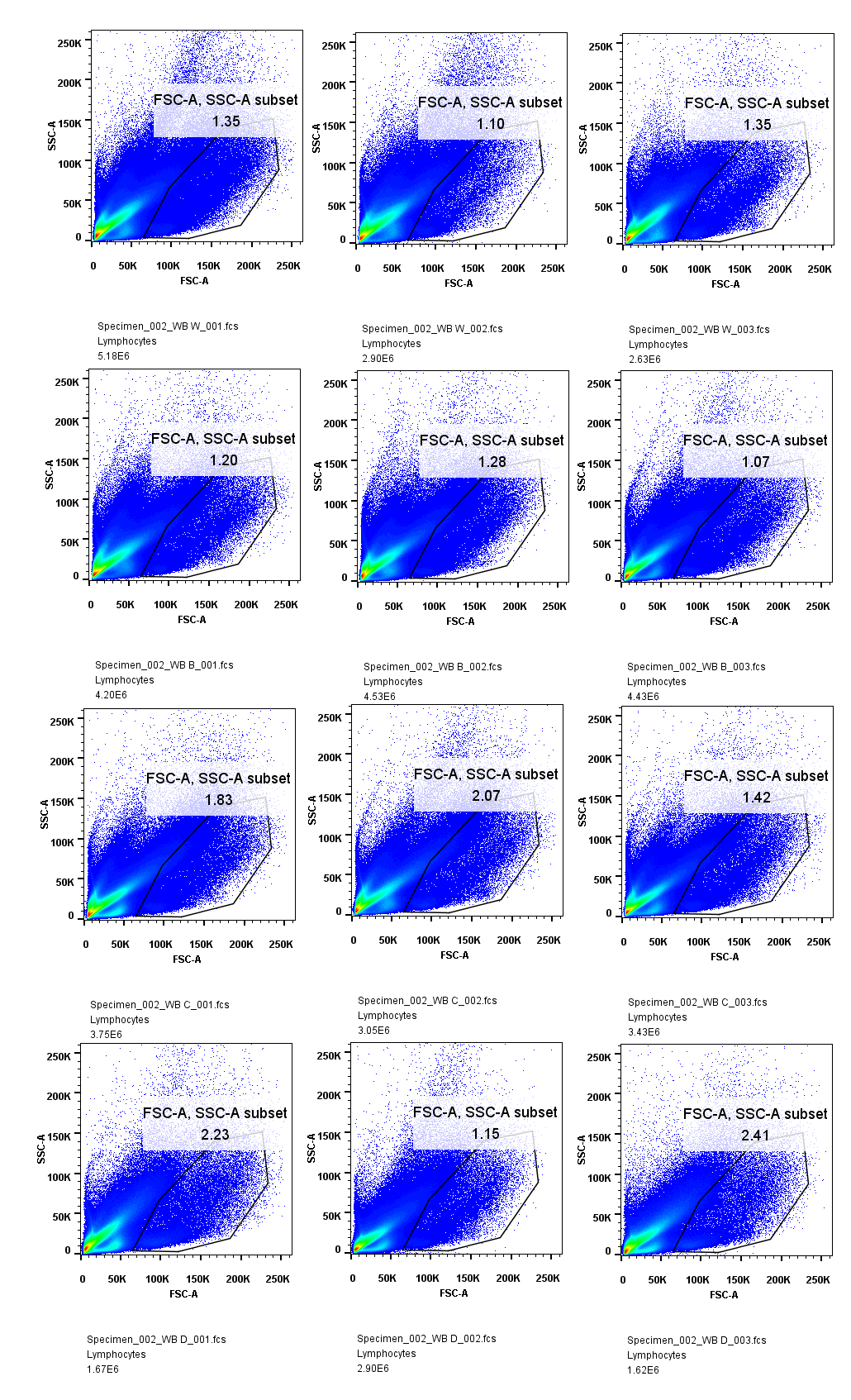
**Supplementary Figure 5A**


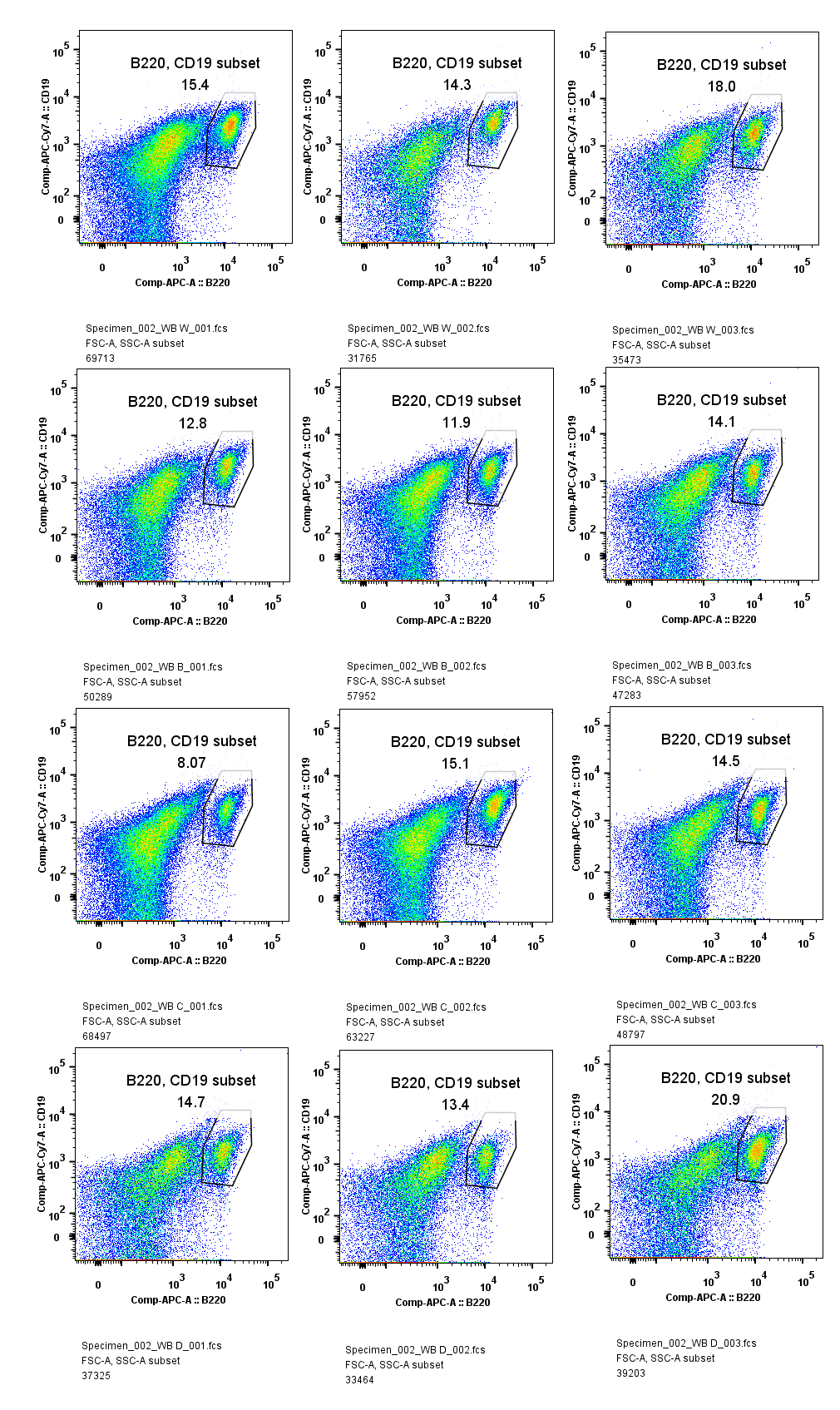
**Supplementary Figure 5A**


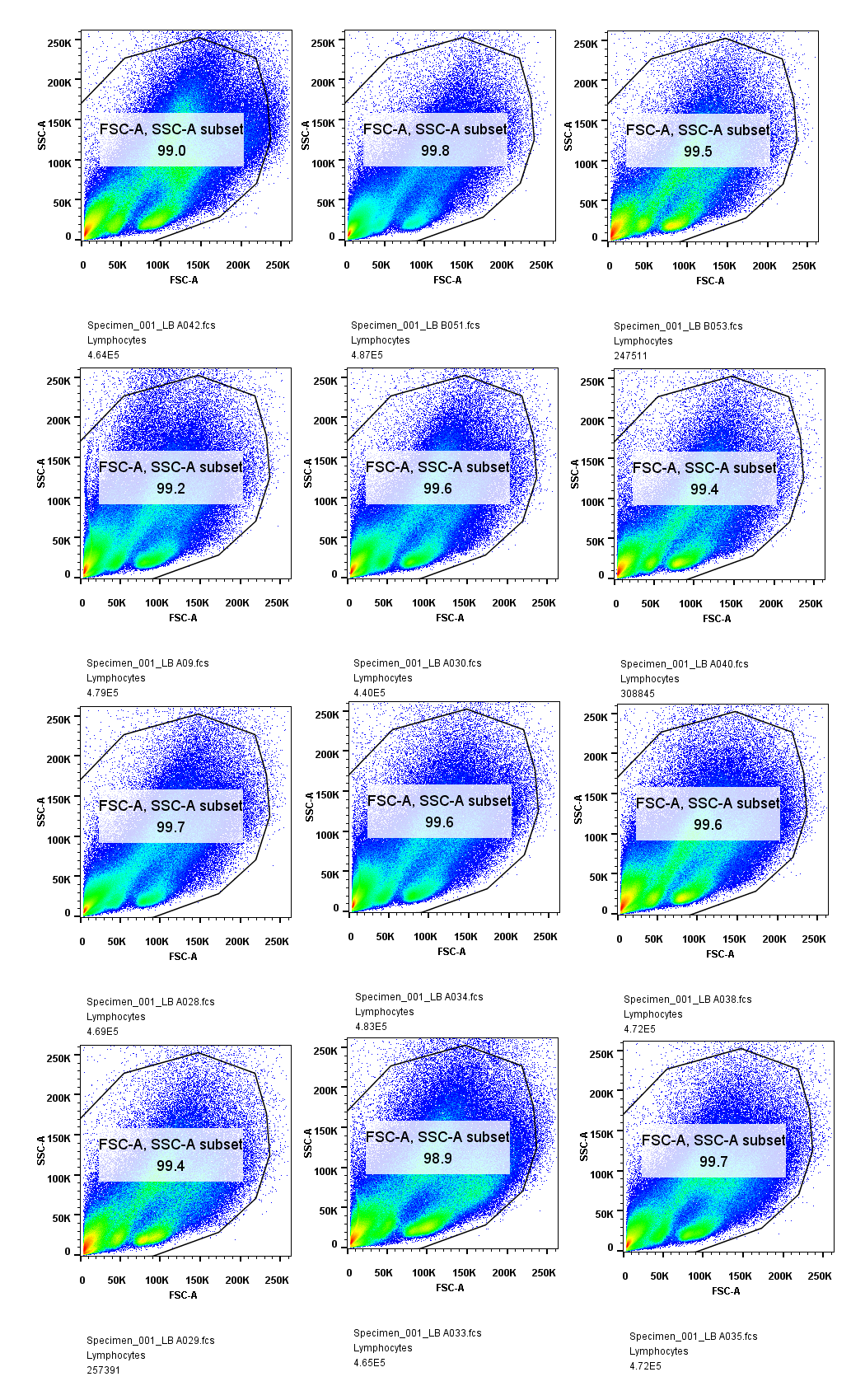
**Supplementary Figure 5C 5E**


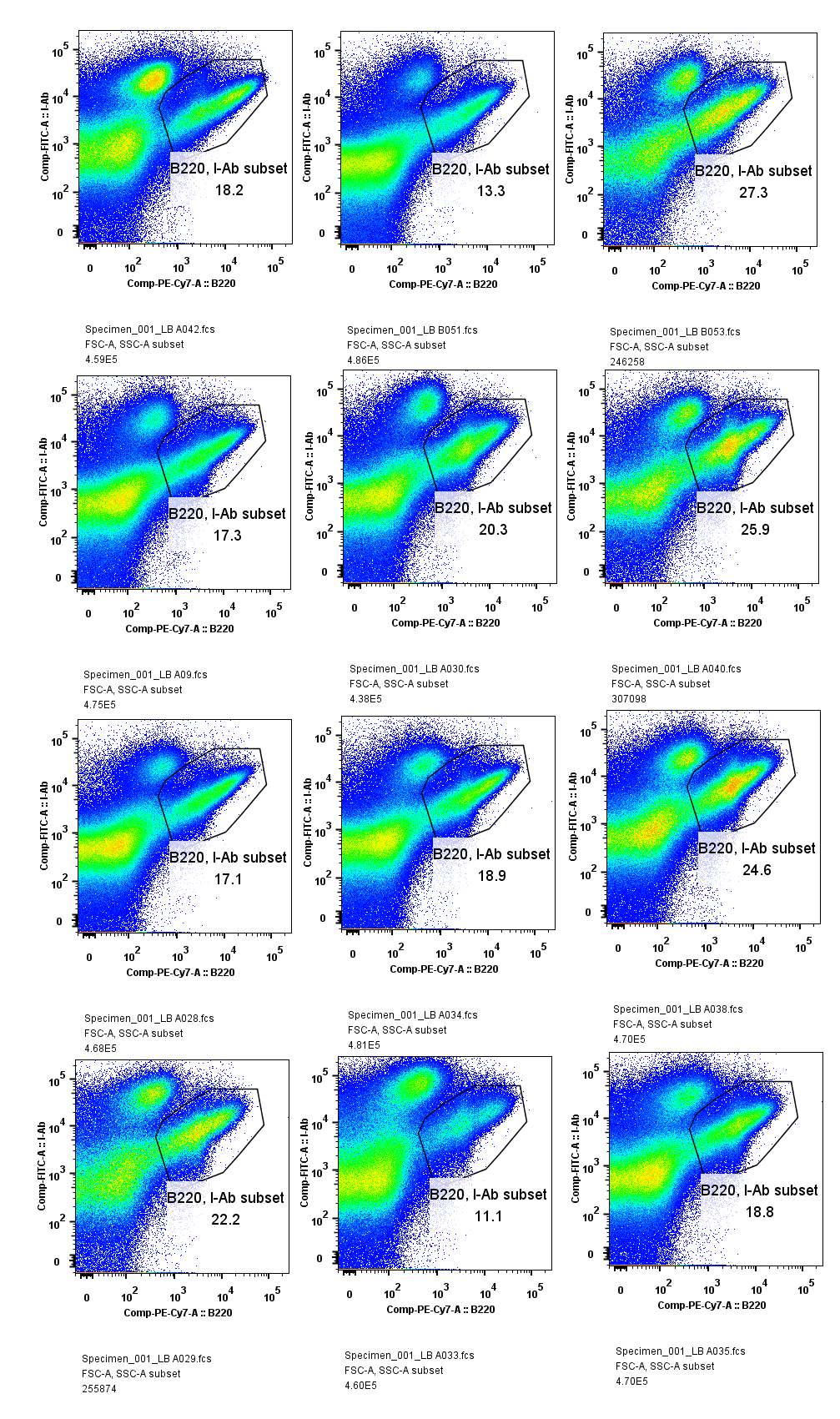
**Supplementary Figure 5C 5E**


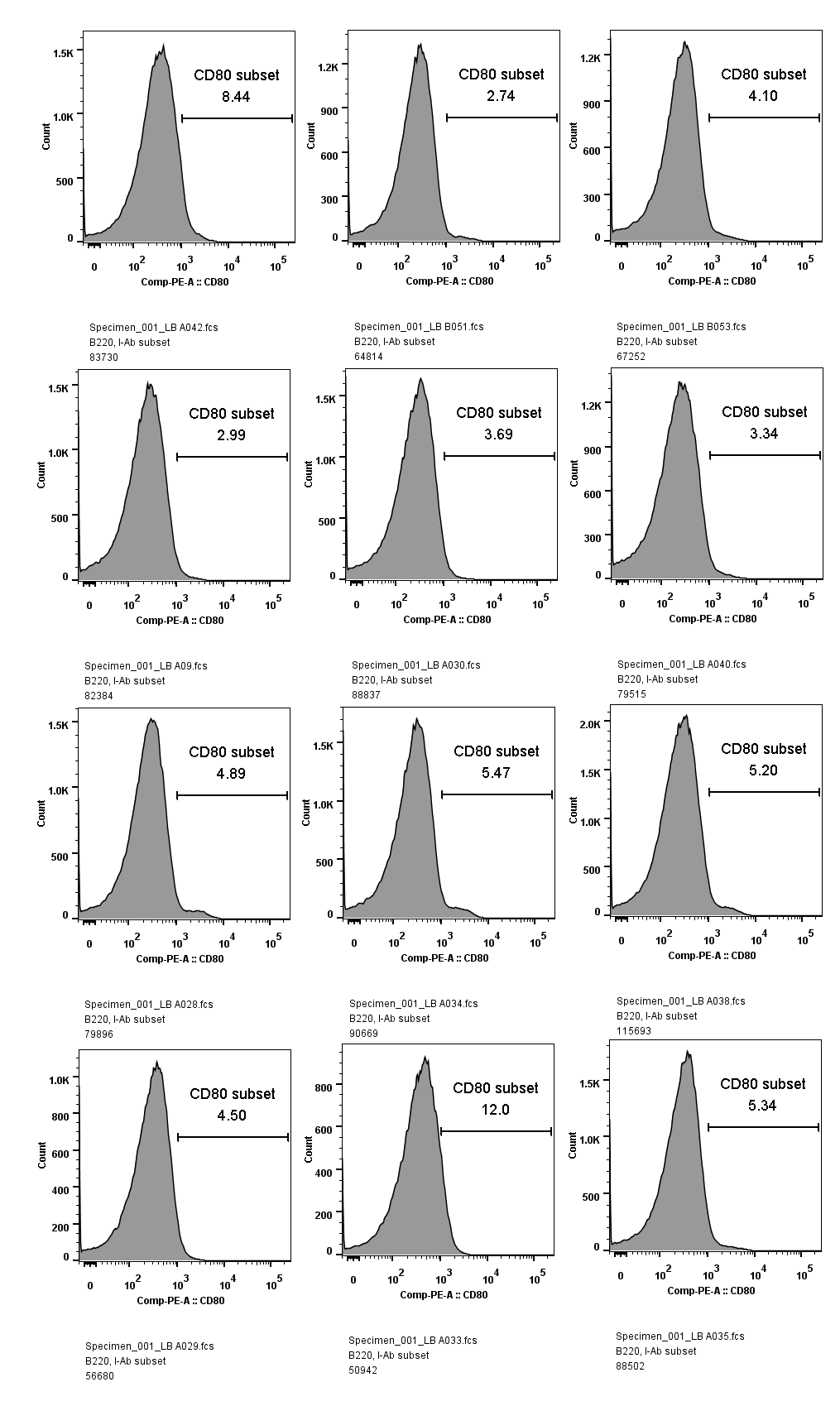
**Supplementary Figure 5C 5E**


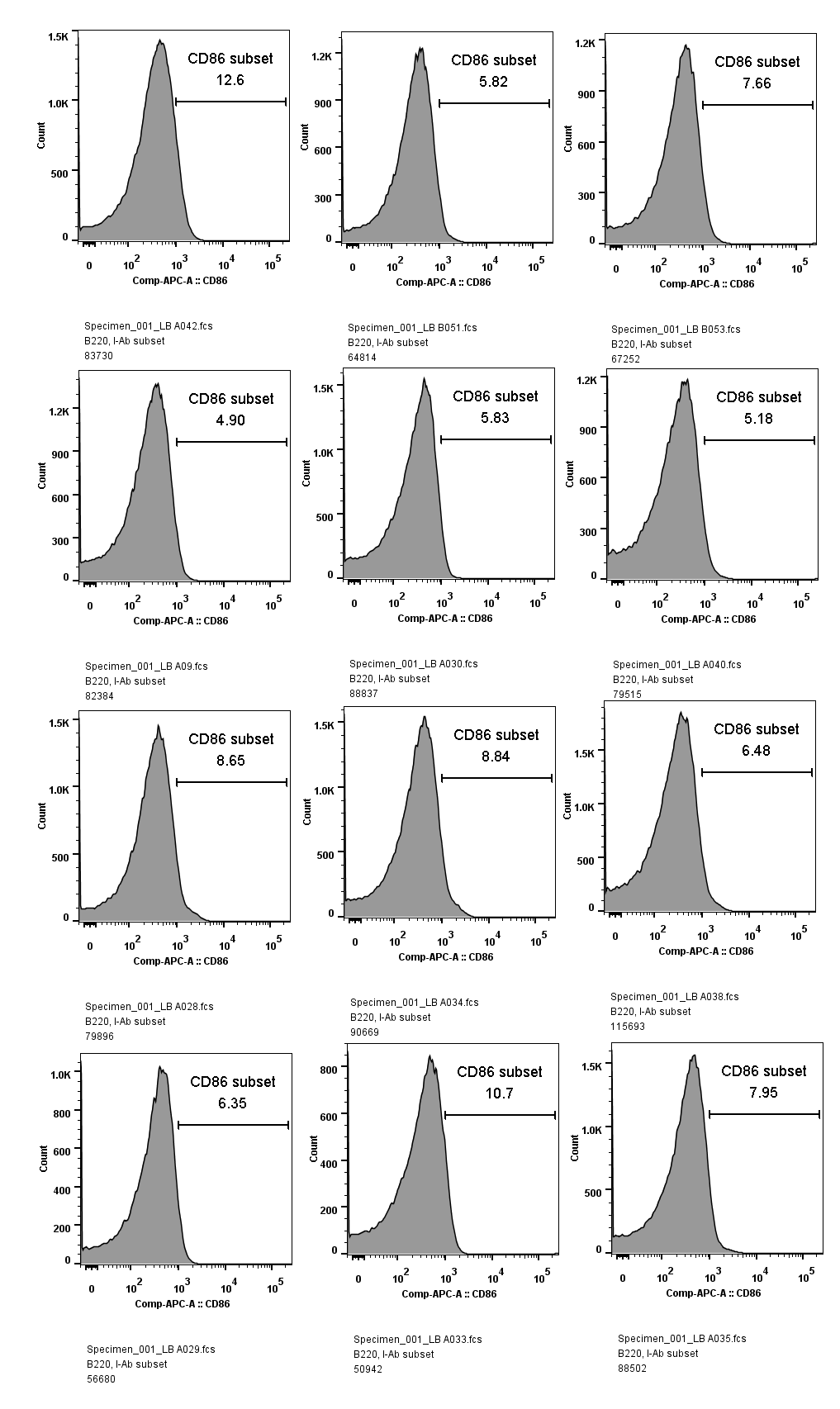
**Supplementary Figure 5C 5E**

**Supplementary Figure 5C 5E**

**WT A042 B051 B052**

**Cbl-b KO A09 A030 A040**

**c-Cbl cKO A028 A034 A038**

**dKO A029 A033 A035**
