# Supplementary material for: Ablation of Cbl-b and c-Cbl in dendritic cells causes spontaneous liver cirrhosis via altering multiple properties of CD103+ cDC1s
Source: Cell Death Discov. 2022 Mar 30;8:142. doi: 10.1038/s41420-022-00953-2 (PMC8967913; doi:10.1038/s41420-022-00953-2)

Figure 1 a

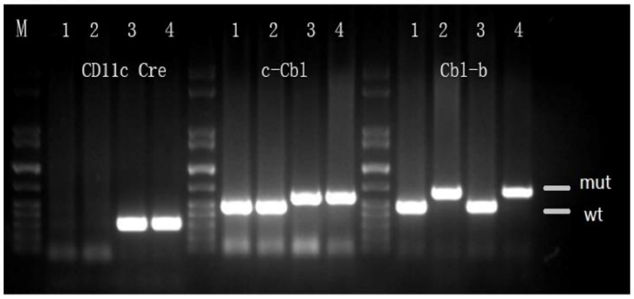

Figure 1b

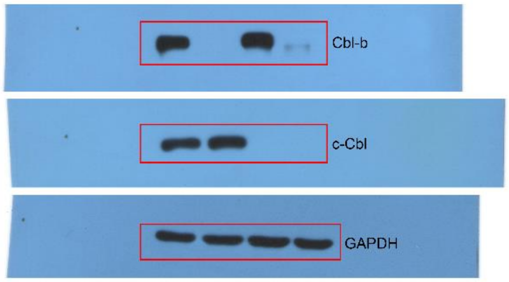

Figure 3 e

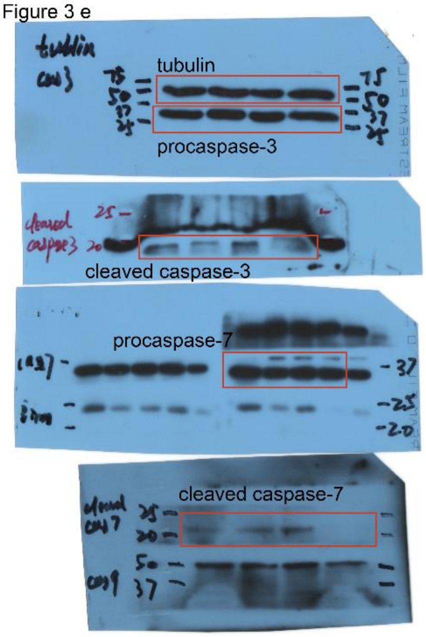

Figure 5 a

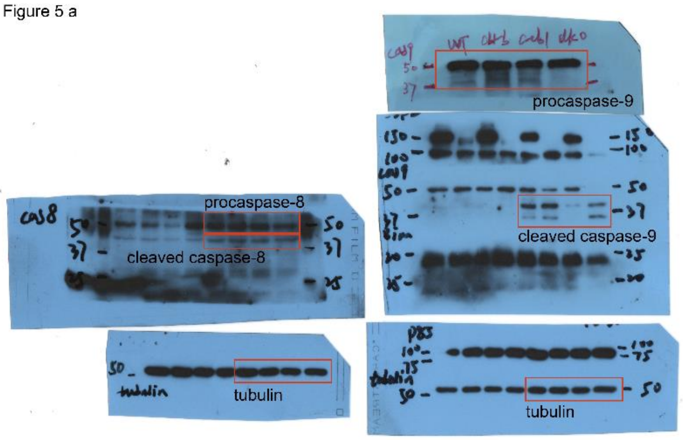

Figure 5 e

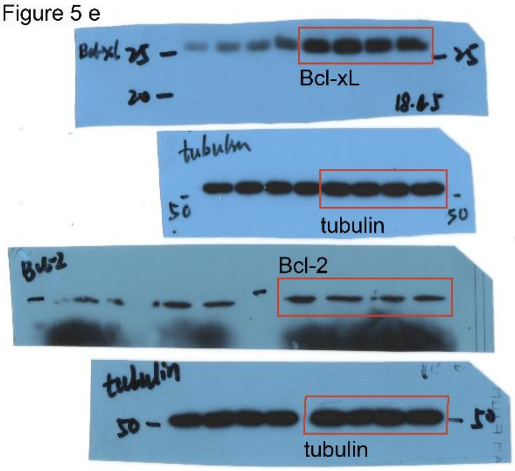

Figure 5 c

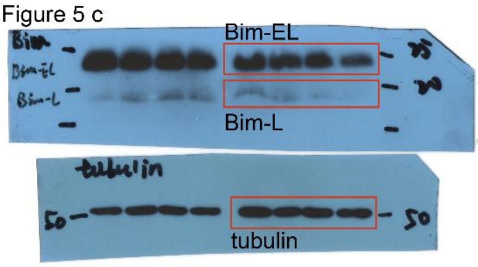

Figure 5 f

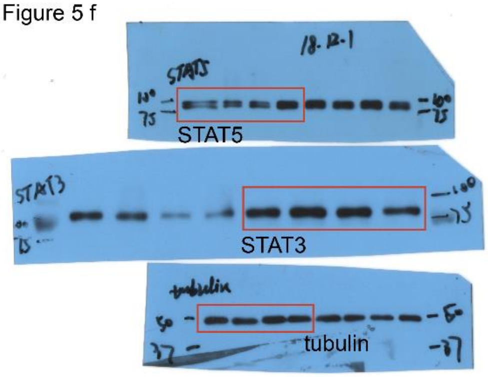

Figure 6 a

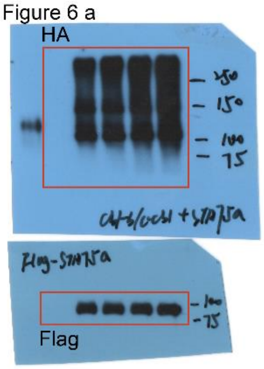

Figure 6 b

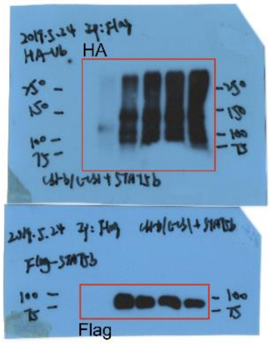

Figure 6 c

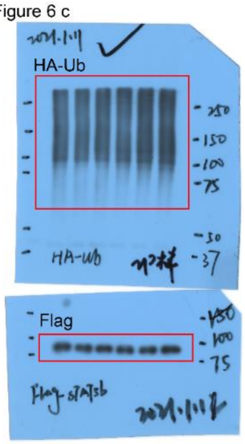

Figure 6 d

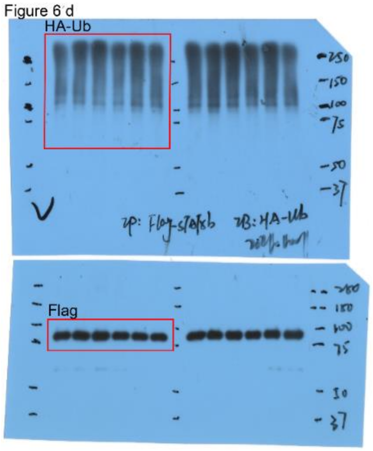

Figure 6 e (upper)

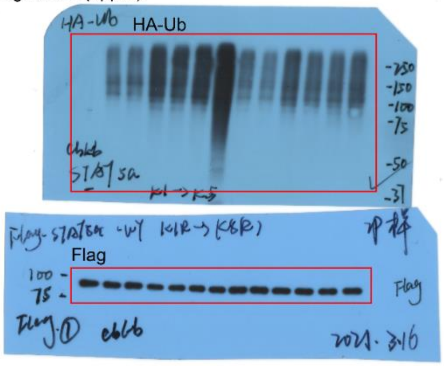

Figure 6 f (upper)

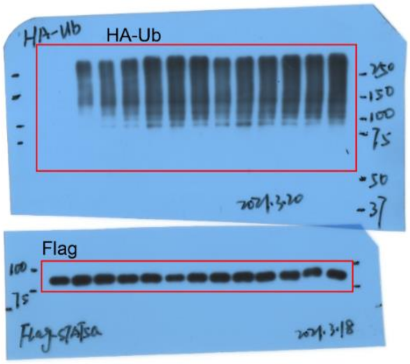

Figure 6 e (middle)

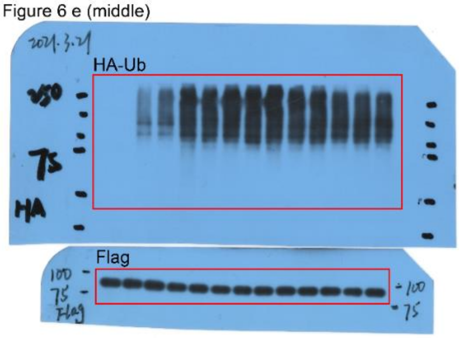

Figure 6 f (middle)

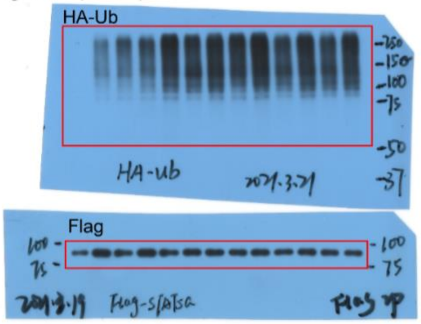

Figure 6 e (lower)

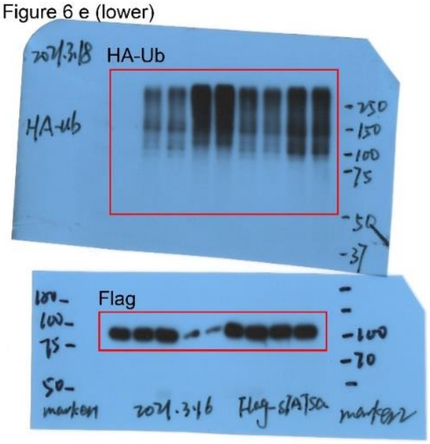

Figure 6 f (lower)

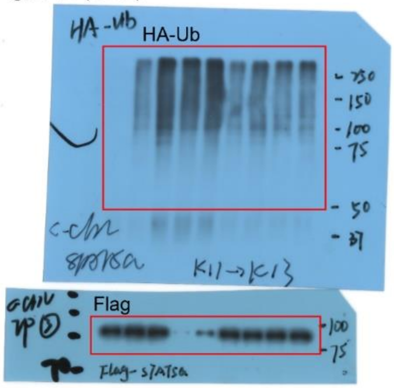

Figure 7 a

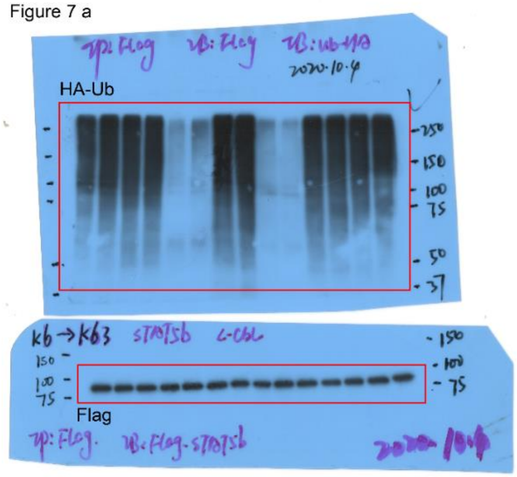

Figure 7 b

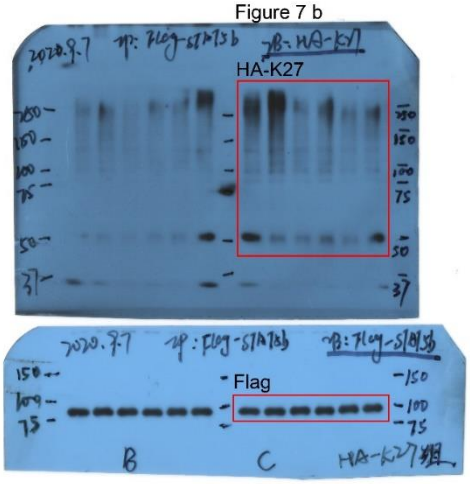

Figure 7 c

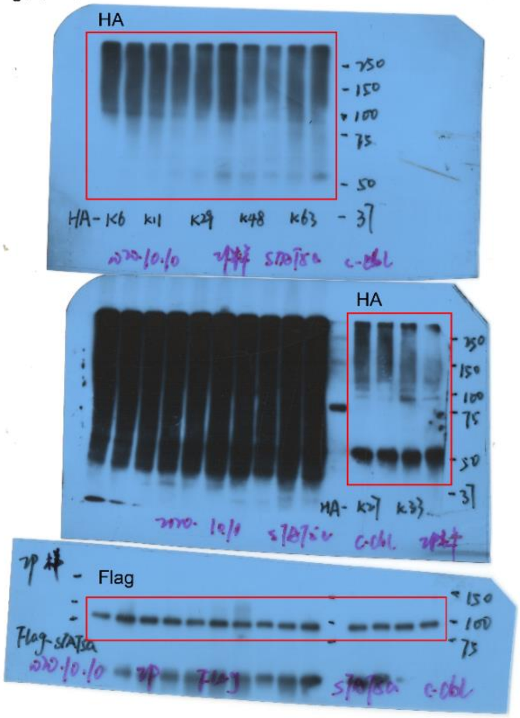

Figure 7 d

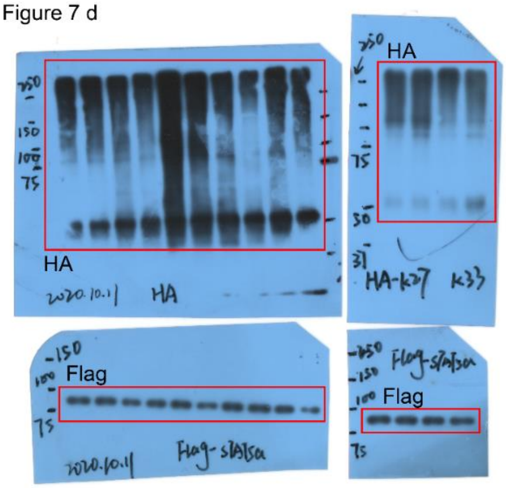

Supplement: Supplementary file 3 — Original Data File [file 41420_2022_953_MOESM3_ESM.pdf]
