# Supplementary figures and images for: Ablation of Cbl-b and c-Cbl in dendritic cells causes spontaneous liver cirrhosis via altering multiple properties of CD103+ cDC1s
Source: Cell Death Discov. 2022 Mar 30;8:142. doi: 10.1038/s41420-022-00953-2 (PMC8967913; doi:10.1038/s41420-022-00953-2)

**Figure 1E**

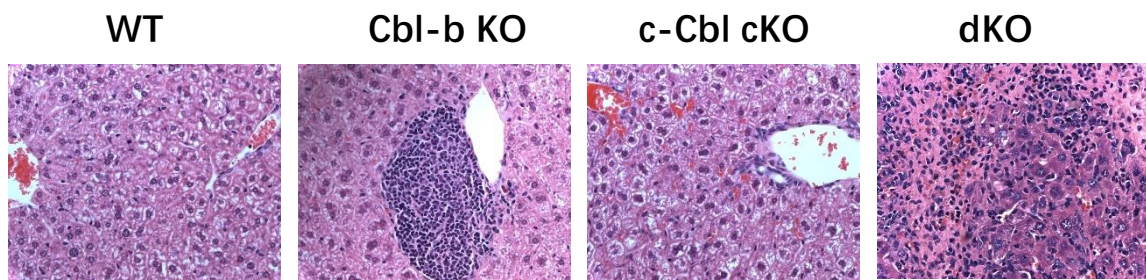

**Figure 1F**

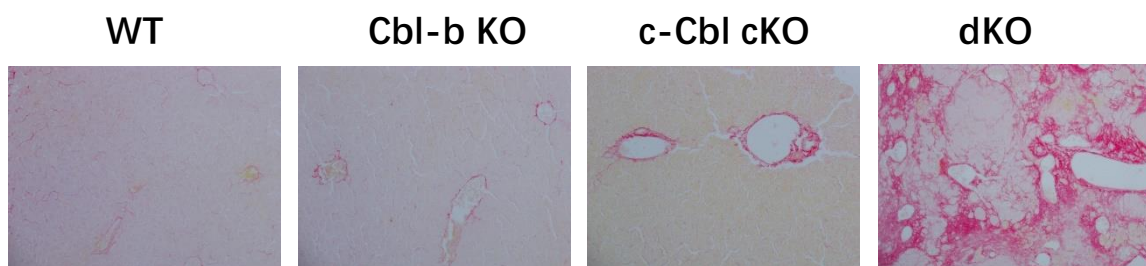

**Figure 4I**

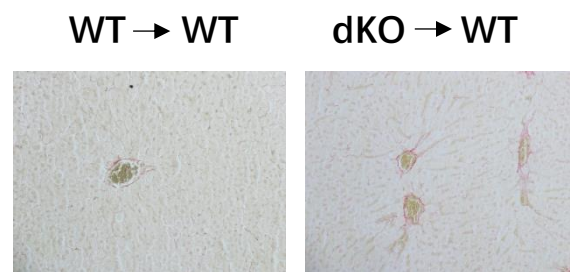

## Supplementary Figure1

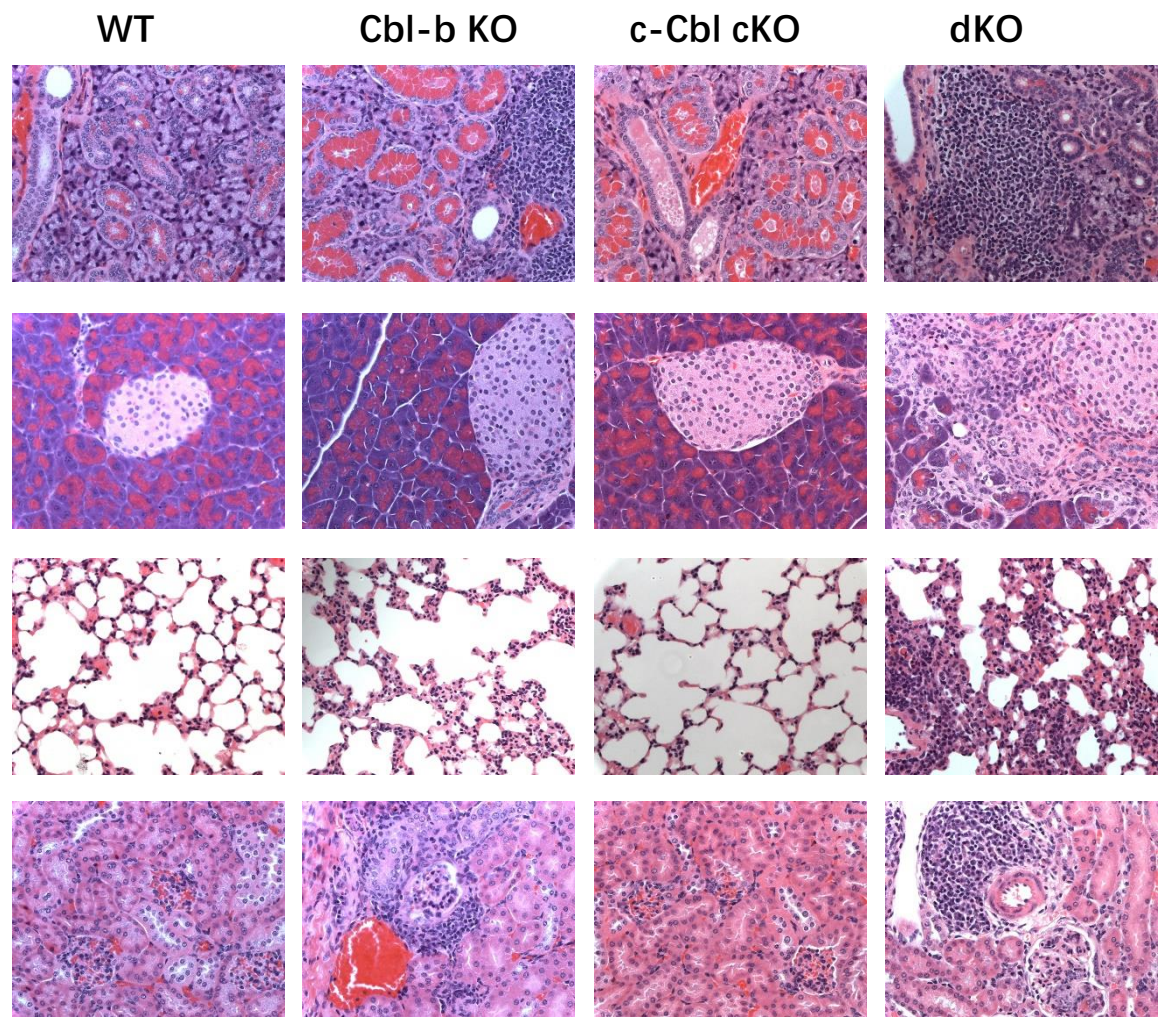

Supplement: Supplementary file 4 — Original Data File [file 41420_2022_953_MOESM4_ESM.pdf]
